# Anticancer Agents as Design Archetypes: Insights into the Structure–Property Relationships of Ionic Liquids with a Triarylmethyl Moiety

**DOI:** 10.1021/acsphyschemau.2c00048

**Published:** 2022-12-07

**Authors:** Grace I. Anderson, David Hardy, Patrick C. Hillesheim, Durgesh V. Wagle, Matthias Zeller, Gary A. Baker, Arsalan Mirjafari

**Affiliations:** † Department of Chemistry and Physics, Florida Gulf Coast University, Fort Myers, Florida 33965, United States; ‡ Department of Chemistry and Physics, Ave Maria University, Ave Maria, Florida 34142, United States; § Department of Chemistry, Purdue University, West Lafayette, Indiana 47907, United States; ∥ Department of Chemistry, University of Missouri-Columbia, Columbia, Missouri 65211, United States; ⊥ Department of Chemistry, State University of New York at Oswego, Oswego, New York 13126, United States

**Keywords:** functionalized ionic liquids, triarylmethyl moiety, structure−property relationships, Hirshfeld analysis, DFT simulations

## Abstract

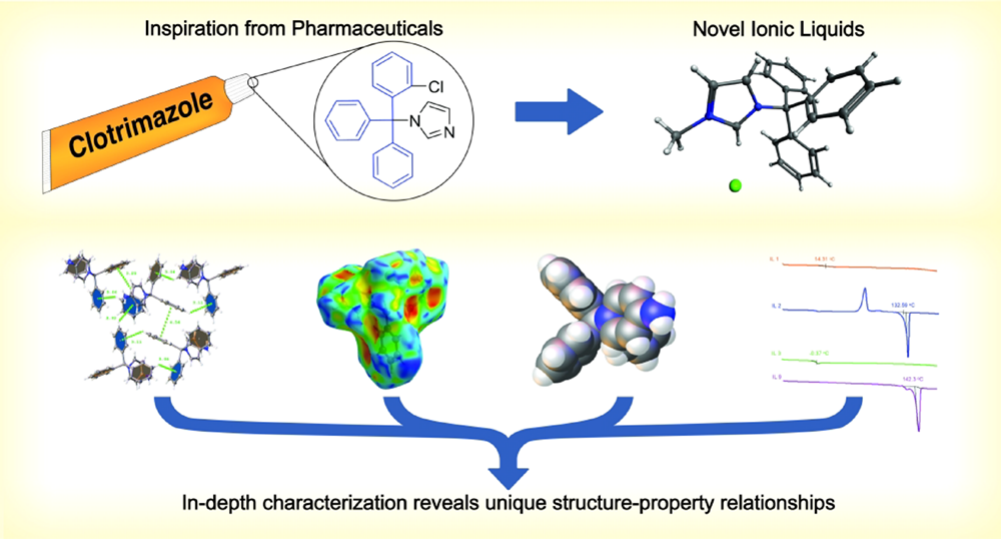

A fundamental challenge underlying the design principles of ionic liquids (ILs) entails a lack of understanding into how tailored properties arise from the molecular framework of the constituent ions. Herein, we present detailed analyses of novel functional ILs containing a triarylmethyl (trityl) motif. Combining an empirically driven molecular design, thermophysical analysis, X-ray crystallography, and computational modeling, we achieved an in-depth understanding of structure–property relationships, establishing a coherent correlation with distinct trends between the thermophysical properties and functional diversity of the compound library. We observe a coherent relationship between melting (*T*_m_) and glass transition (*T*_g_) temperatures and the location and type of chemical modification of the cation. Furthermore, there is an inverse correlation between the simulated dipole moment and the *T*_m_/*T*_g_ of the salts. Specifically, chlorination of the ILs both reduces and reorients the dipole moment, a key property controlling intermolecular interactions, thus allowing for control over *T*_m_/*T*_g_ values. The observed trends are particularly apparent when comparing the phase transitions and dipole moments, allowing for the development of predictive models. Ultimately, trends in structural features and characterized properties align with established studies in physicochemical relationships for ILs, underpinning the formation and stability of these new lipophilic, low-melting salts.

## 1 Introduction

**Figure 1:**
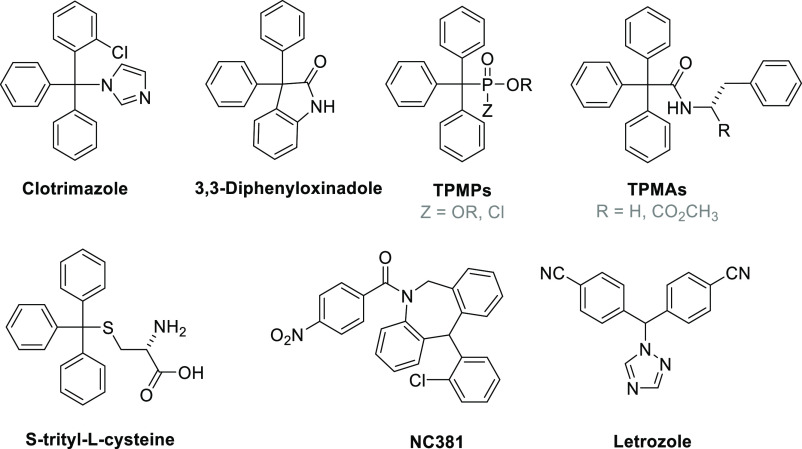
Triphenylmethane as a privileged structural motif with anticancer properties.

First reported by Lewis and Goland in 1953,^1^ the antitumor activity of organic dyes having a triarylmethyl (trityl) backbone was demonstrated by testing 235 commercially available compounds. In the period since, these triarylmethyl-bearing compounds have become well-established as anticancer agents owing, in part, to the unique structural arrangement and lipophilic nature of the trityl group.^2−7^ A canonical example of this class of compounds is Clotrimazole, which was originally developed as an antifungal agent for treating fungal skin infections such as athlete’s foot and ring worm. Recently, Clotrimazole has also been shown to exhibit considerable inhibitory effects on both cancer cell proliferation and tumor growth (Figure 1).^8^ The triarylmethyl moiety was uniquely identified for its anticancer activity through systematic elimination of other imidazole-based antimitotics. Further evidence was established in which the isolated moiety in its triarylmethanol form also displayed anticancer activity.^8^ As such, many other triarylmethyl-containing compounds, including *S*-trityl-*L*-cysteine as well as various triphenylmethylamides and triphenylmethylphosphonates, also exhibit anticancer properties through mechanisms, which arrest cancerous cell growth at various cell cycle phases or by inducing apoptosis.^7^ The primary limitation of Clotrimazole and its analogues toward cancer therapeutics stems from the incredibly poor bioavailability arising from the lipophilic nature of the triarylmethyl moiety.^9^ Beyond anticancer and antifungal pharmaceuticals, triarylmethyl-based compounds have found further applications for synthetic dyes,^10−12^ catalysis,^13,14^ and coordination chemistry, conferring a broad relevance of the triarylmethyl group to several disciplines.^15^

The molecular and material discovery of ILs has been extensively researched due to their structural diversity and their appealing and tunable characteristics (e.g., vanishingly low vapor pressure and high thermal and chemical stability). ILs have found broad appeal across numerous fields of study as both innovative materials and solvents for academic research and industrial applications alike.^16−18^ Notwithstanding the ubiquity of publications and patents, the broader IL field is still considered a young discipline and offers enormous potential for the design and construction of new and emergent materials having superlative properties and function. Although ILs have already helped to usher in new technologies,^17^ critical issues remain, especially biocompatibility, purity, stability, and cost.^19^ In fact, the modern IL field is characterized (theoretically and experimentally) by an increasing emphasis on the identification of specific design elements that are critical to performance.^20^ There is also a growing appreciation for the need to integrate molecular design principles based on insights gleaned from adjacent and remote fields of chemistry. For example, lessons learned from medicinal chemistry offer prospects for constructing novel, task-specific ILs incorporating diverse functionality leading to improved biocompatibility, stealth properties, and target specificity.

Our goal of unraveling the relatively unexplored and inextricable tangle of structure–property–function relationships within ILs coincides with a parallel interest in the triarylmethyl moiety as a component for unique structural diversity and biocompatibility. As such, we have initiated an exploration into the correlations between the structures and properties of triarylmethyl-functionalized ILs bearing a hydrophobic motif that does not incite toxicity while delivering low melting points. Given its prevalence in the field, Clotrimazole was identified as a model compound for this endeavor, as it contains both an imidazole headgroup and a triarylmethyl moiety.

The triarylmethyl moiety is a sterically hindered and nonpolar motif, creating a large hydrophobic/nonpolar pocket within the molecule that results in extremely low aqueous solubility.^21^ This ideally positions Clotrimazole-inspired ILs to possess a desired balance between biocompatibility and solubility. In light of these considerations, the development of ILs containing various triarylmethyl moieties emerges as a significant strategy for widening the catalog of available ILs, while also establishing key structural diversity rubrics for cation design. It was our hypothesis that these therapeutics will prove to be versatile and convenient “tutors” of IL cation design, providing valuable insights into structural elements that simultaneously produce ILs with high fluidity (at ambient or physiological temperatures) and high lipophilicity. Within this work, the structure–property relationships of a suite of a dozen trityl-functionalized ILs were evaluated using thermophysical methods, [(differential scanning calorimetry (DSC) and thermogravimetric analysis (TGA)], single crystal X-ray diffraction (SC-XRD), and computational analysis.

## 2 Results and Discussion

### 2.1 Ionic Liquid Design Rationale

**Figure 2:**
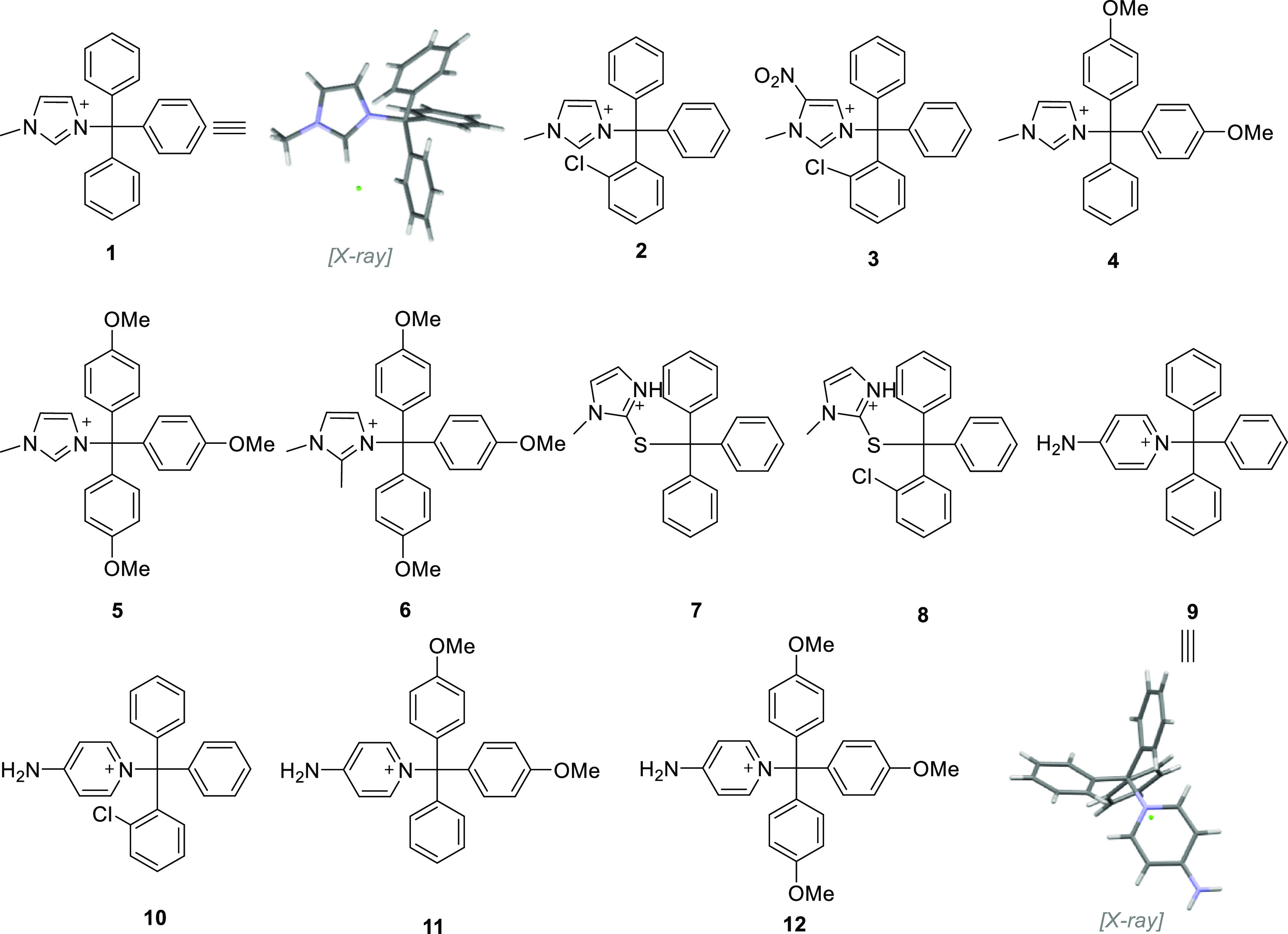
Structures of new triarylmethyl-bearing ILs **1–12**, using diverse *N*- and *S*-heterocycles as synthetic precursors. The chloride counter anions are omitted for clarity. The numbering corresponds to the entities specified throughout the text.

We synthesized 12 new ionic compounds bearing a triarylmethyl motif (Figure 2) via a one-step substitution reaction to form a cationic moiety, following established synthetic strategies. Several key design elements were carefully considered as we selected compounds for synthesis and subsequent analysis. First, we sought to systematically vary the aromatic heterocycle serving as the cation backbone, primarily via imidazole or pyridine derivatives. Second, functionalization of the triarylmethyl moiety was achieved through various derivatives with 2-chloro- and 4-methoxy-substituents. Finally, we elected to employ active pharmaceutical ingredients (APIs) and their precursors to simultaneously introduce structural diversity into the ILs in *a priori* anticipation of reduced toxicities. The API precursors used in this endeavor include 5-nitroimidazole (**3**), the backbone of various antimicrobial compounds,^22^ methimazole (**7**, **8**), commonly used to treat hyperthyroidism,^23^ and dalfampridine (**9**–**12**), a potassium channel blocker used to treat neurological disorders such as multiple sclerosis.^24^ The choice to retain chloride as the anion was made to maintain low toxicity and promote other desirable ionic interactions; this limitation was deemed acceptable within the context of formulating drug-based ILs. This pairing with a hydrophilic anion enhances interactions with water, thus manifesting reduced toxicity compared to lipid-inspired ILs comprising the bistriflimide ([(CF_3_SO_2_)_2_N]^−^) anion.^25,26^

Synthetically, the general reaction proceeds as a quaternization reaction between triarylmethyl chloride and an aromatic *N*- or *S*-heterocyclic headgroup at ambient temperature (except for compound **2**) per Scheme S1. Due to the low solubility of the starting materials, heating at 50 °C for 3 days was required for compound **2**, after which significant precipitation was observed. The workup process was minimal, generally requiring only simple filtration or solvent rotary evaporation. Of note, these salts are prone to hydrolysis under wet conditions, producing the hydrochloride salt of the heterocyclic headgroup while liberating triarylmethyl alcohol (e.g., compound **2a**). However, this water-induced bond cleavage appears to be potentially advantageous from the standpoint of drug delivery, assuming that a regulated rate of hydrolysis can be realized. As such, the mechanism of this hydrolysis was further examined using data gathered from SC-XRD and computational analysis (vide infra).

### 2.2 Structure–Physicochemical Property Relationships

The new ILs were evaluated by DSC, and the resulting thermophysical data, including melting point (*T*_m_) and glass transition temperature (*T*_g_) values, are compiled in Table 1. Given the high viscosity of these ILs, slow ramp rates and iterative heating/cooling cycles were necessary to accurately establish the thermal phase transitions for these compounds. The complete DSC traces are provided in the Supporting Information. Overall, these ILs exhibit controllable values of *T*_m_ and *T*_g_. Specifically, the addition, deletion, or combination of dipolar modules on the triarylmethyl motif allows for fine-tuning of the phase transitions. To explain these observations, the ILs were studied by molecular simulation using the M06-2X functional with a 6-31++G(d,p) basis set. The combination of the M06-2X/6-31++G(d,p) theory and basis set has been successfully applied to predict structure and the electronic and thermodynamic properties of ILs^27^ as well as similar systems containing ionic species.^28^ The simulations allowed us to rationalize the observed changes of *T*_m_/*T_g_* via tailoring of both the structure and dipole moment of the studied ILs.

**Table 1:** Experimental Melting Points, Glass Transition Temperatures, Thermal Stabilities, and Calculated Dipole Moment Data at the M06-2x/6-31++G(d,p) Theory and Basis Set for Triarylmethyl-Derived Salts **1**–**12**^a^

IL	*T*_m_ (°C)	*T*_g_ (°C)	*T*_onset5%_ (°C)	dipole moment (*D*)
**1**		14.3	142.6	9.43
**2**	129.7		162.3	11.55
**3**	68.9		143.5	13.53
**4**		29.4	150.5	9.50
**5**		35.5	143.8	8.11
**6**		55.9	151.9	8.16
**7**			152.5	10.49
**8**	87.9		166.7	10.43
**9**	142.3		169.1	8.68
**10**	155.7		168.8	11.62
**11**		66.8	213.1	6.26
**12**		67.4	227.2	7.15

a The data were calculated as the standard deviation of the mean of three trials with standard uncertainty of ±0.3 °C or less for each reported data point.

Nine of the salts display values of *T*_m_/*T_g_* below the arbitrary but popular benchmark for ILs of 100 °C. Compounds **2**, **9**, and **10** formed mesothermal ILs, a term coined by Davis and coworkers,^29^ and are associated with *T*_m_ values of 129.7, 142.3, and 155.7 °C, respectively (Table 1). The remaining products exhibited broad endothermic peaks indicative of glass transitions rather than well-defined melting points. This suggests a propensity of triarylmethyl-based compounds to form amorphous solids and glassy liquids. The compounds exhibiting only glass transitions have solid states in which the structural features of the molecule preclude the formation of maximized interionic interactions to form crystalline solids.

The nature of the substituents on the triarylmethyl motif profoundly influences the fluidity and liquefaction behavior of the ILs. Previously, Rabideau et al. demonstrated that for ILs, there is a direct correlation between the *T*_m_ depression and incorporation of an electronegative element into a phenyl moiety of the cation. A notable exception, however, was found wherein modification at the tucked *ortho* position resulted in *increased T*_m_.^30^ This trend is observed in the present set of compounds with seemingly subtle structural modifications dramatically modulating the thermal phase transitions of the resulting mesothermal salts (i.e., **1** → **2** and **9** → **10**). For instance, IL **1** bears an unsubstituted triarylmethane group and features *T*_g_ ≈ 14 °C. Monochlorination of the ring (**2**) generates an IL with a distinct melting transition at 129.7 °C. A parallel trend can be seen comparing **9** and **10**, in which chlorination markedly increases *T*_m_ by 13.4 °C.

Rabideau et al. noted that an increase in the cation’s dipole moment (*D*) due to substitution of hydrogen with an electron-withdrawing group such as fluorine at *ortho* positions of the trityl cation tends to result in a decrease in *T*_m_ through the reduction of liquid phase enthalpy.^31^ This trend is in accordance with observations for the ILs studied herein (see Table 1 for the dipole moment values of the ILs). A more nuanced modification concerns the substitution of hydrogen with chlorine at the *ortho* position. Strikingly, while this increases the *D* values, suggesting that one might observe a decrease in *T*_m_, the bulkier electron-donating nature of the chloride group paired with its “tucked” *ortho* position prevents extended dipole–dipole interactions and ultimately provides the opposite effect of increasing the *T*_m_ concomitant with an increased dipole moment. This notable exception to the observed trend was also identified by Rabideau et al.,^31^ and our current observations into the effect of dipole moment orientation further elucidate this anomaly. Comparatively, the established correlational patterns between dipole moment and *T*_m_ can be observed with the introduction of the electron-withdrawing nitro group (**2** → **3**) into the imidazolium ring, which enhances the positive charge in the ring significantly and lowers *T*_m_ with increase in the dipole moment (Table 1).

A further observation is that the presence of methoxy groups at the *para* positions of the triarylmethyl moiety (**4**–**6**, **11**, and **12**) consistently result in ILs having subambient glass transitions and the absence of distinct melting points. This indicates that this substitution strongly contributes to the formation of amorphous solids over ordered packing, further supported by the inability to successfully grow single crystals of the indicated salts. Due to the electron-donating nature of the *para*-substituted methoxy groups, we suspect that the charge separation/polarity is likely mitigated through resonance, resulting in a *T*_g_ decrease. Furthermore, the presence of the methoxy groups increases the bulk and asymmetry of the cation, potentially frustrating packing and increasing the entropy of the resulting salts. Collectively, the previous data indicate that there is considerable structural latitude possible when designing highly lipophilic ILs that exhibit profitably low values of *T*_m_/*T*_g_. Learning from the present examples, the inclusion of polar substitutions on the aromatic rings appears to be a powerful downward driver of *T*_m_ and *T*_g_.

Established correlations between the dipole moment and *T*_m_ are illustrated by installation of an electron-withdrawing nitro group (**2** → **3**) onto the imidazolium ring. Indeed, inclusion of the nitro moiety enhances the positive charge in the ring, significantly lowering the *T*_m_ value concomitant with an increased dipole moment. Specifically, **3** has a lower *T*_m_ compared to **2** by nearly 61 °C, demonstrating that the polar nitro group is an effective depressor of *T*_m_. Conversely, methylation at the C-2 position of imidazolium brings about an increase in *T_g_* (by ∼20 °C proceeding from **5** to **6**) driven by a loss of entropy, following thermophysical trends established earlier for 2-methylimidazolium-based ILs.^32^

As noted, our earlier studies provided compelling evidence that incorporation of a thioether chain introduces a major structural disruption to the packing efficiency of an IL, yielding a substantial drop in *T*_m_ when compared to all-carbon analogues.^33^ In line with this, the appended sulfur module at the C-2 position of the imidazolium cation in **7** is likely responsible for the 30 °C drop in *T*_m_ relative to **2**. Even more dramatic are the Δ*T*_m_ values of 66.0 °C between compounds **2** vs **10**. Changing from an imidazolium to a pyridinium cation generally raises *T*_g_/*T*_m_ and, as expected, it was found that a protic amino group pendant on the pyridinium cation brings about a dramatic increase in *T*_m_.^34^ We note that hydrogen bonding is not *singularly* responsible for **9** and **10** having higher *T*_m_ values than **1** and **2**, but rather the observed Δ*T*_m_ likely reflects a net effect accounting also for the impact of increased cation symmetry.

**Figure 3:**
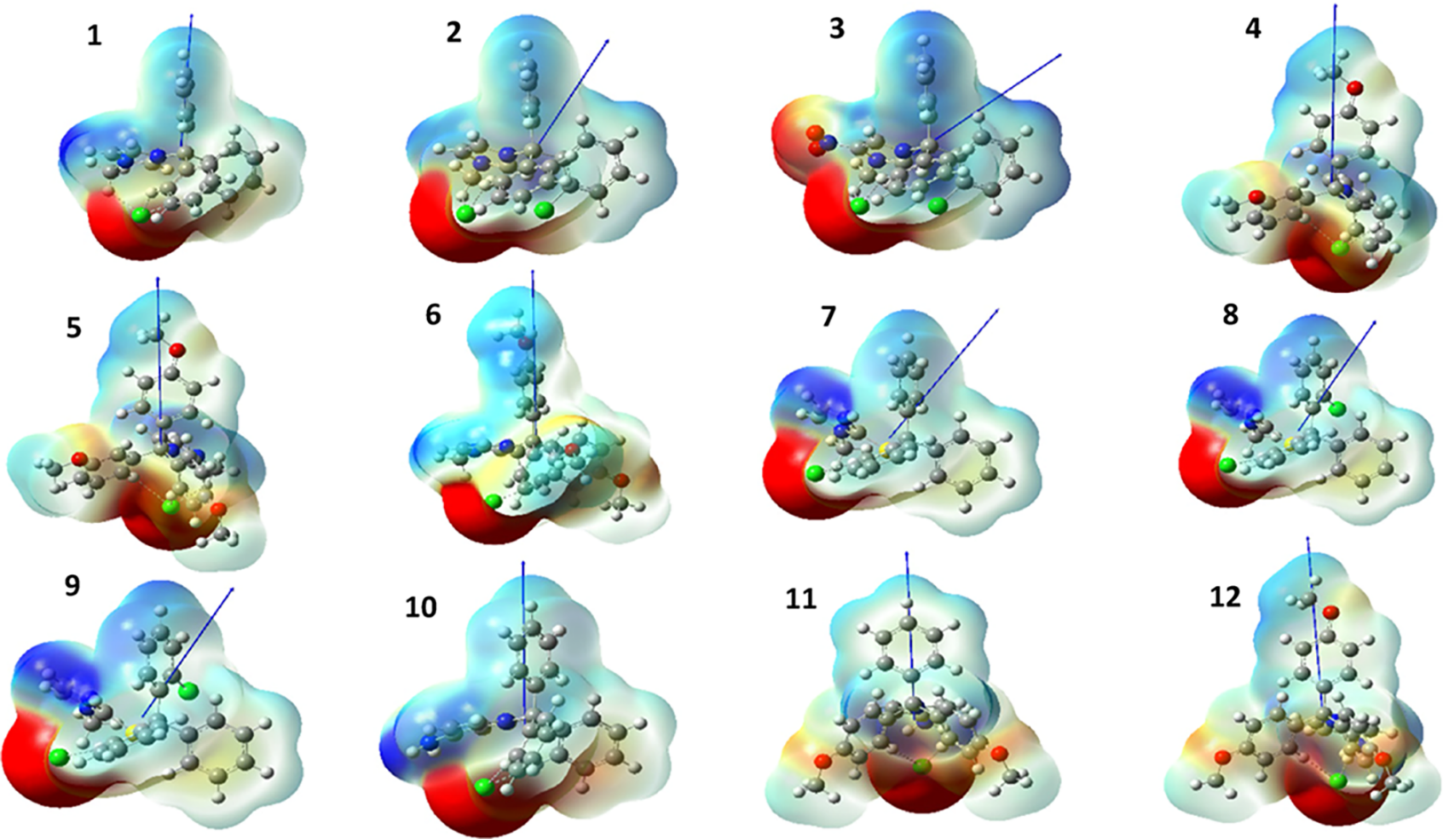
Optimized geometry, dipole moment (blue arrow), and electrostatic potential surface for ILs **1**–**12** calculated at M06-2X/6-31g++G(d,p) theory and basis set. The red regions indicate higher electron density, whereas the dark blue regions denote low electron density.

Following on previous studies evaluating the impact of the dipole on physicochemical properties of the ILs, we performed quantum chemical simulations in order to evaluate the electronic environment associated with the different substitutions on the rings. As expected, the electron density mapping obtained using electrostatic potential (ESP) shows that electrons are more concentrated on the chloride anion (red coloring), whereas the cation ring bears the least electrons (blue coloring) (Figure 3). In contrast, the trityl moiety bears intermediate electron density, as indicated by light blue and yellow regions. The ESP mapping clearly depicts the change in the electron density and the corresponding change in the orientation of the dipole caused by the varying substitutions on either the heterocycle or the trityl moiety (Figure 3).

**Figure 4:**
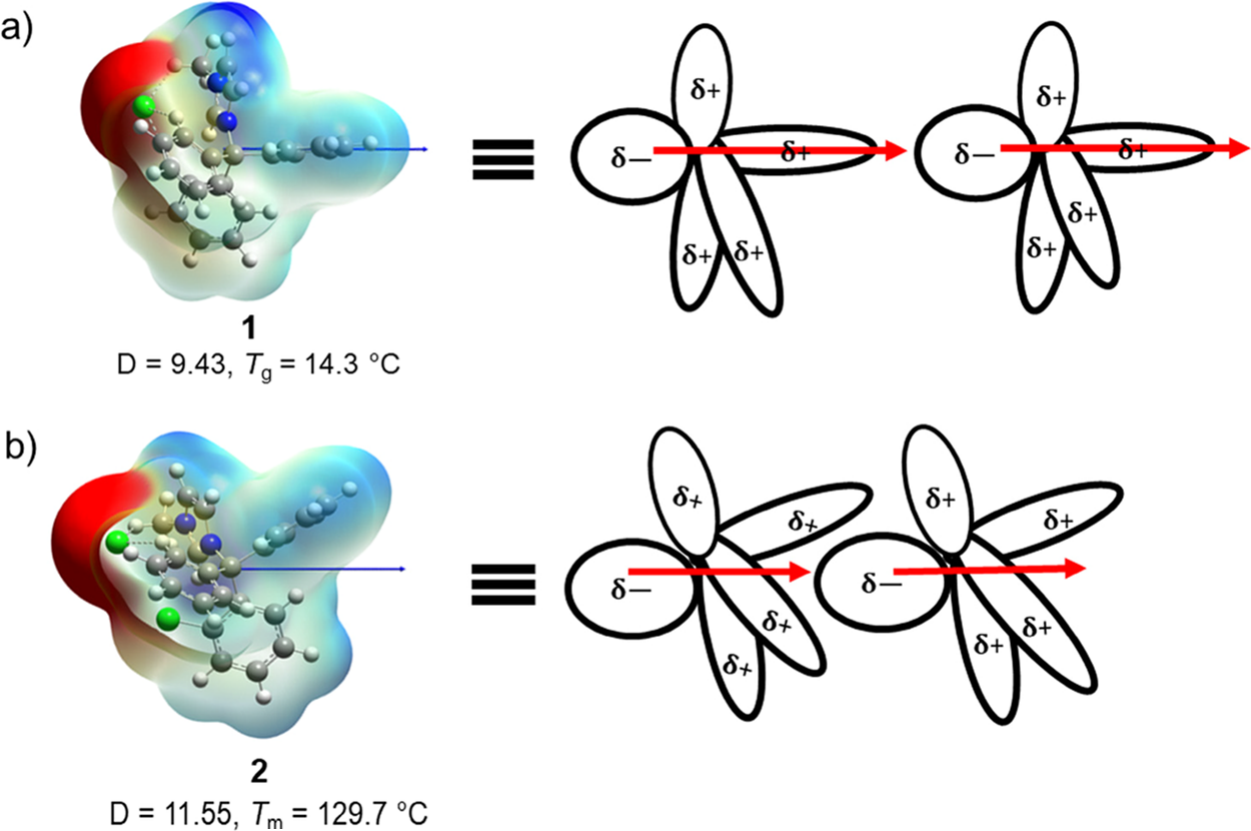
Proposed scheme displaying the influence of dipole moment orientation on the intermolecular interaction and its relationship to observed *T*_m_ and *T*_g_ for the salts.

For instance, in **1**, the direction of the dipole is aligned along the principal axis of the phenyl ring of the trityl moiety, whereas in **2**, introduction of the chlorine at the *ortho* position leads to the dipole to reverse orientation, aligning between phenyl rings. Notably, when the orientation of the dipole passes through the plane of one of the phenyl rings on the trityl moiety, the compound displays a glass transition (see Figure 3 and Table 1). In contrast, in compounds where the dipole is oriented between the phenyl rings, a distinct *T*_m_ is observed, except for **10** (Figure 4). The relationships between the orientation of the dipole in ILs and its influence on their *T*_m_/*T*_g_ can be best rationalized by using a proposed intermolecular interaction between IL salts, as described in Figure 4. When the orientation of the dipole passes through the plane of a phenyl ring of the trityl moiety, it results in a linear end-to-end dipole–dipole interaction between IL salts causing minimal contact that leads to a weak intermolecular interaction, and as a result, the IL species exhibit a *T*_g_ (Figure 4a). However, when the dipole vector aligns between the phenyl rings of the trityl moiety, the linear end-to-end dipole–dipole interaction allows for one IL salt to wedge between the phenyl rings of the trityl group of second IL salt (Figure 4b). This “wedging” allows for ion pairs to come closer together, resulting in better intermolecular contacts, thus leading to stronger interactions. This is a clear indication that the electronic perturbation caused by substitution reorients the dipole, which can be experimentally observed through changes in the *T*_m_/*T*_g_ of the molecule. This means that it is possible (at least in these examples) to take a basic cation framework and temper the impact that one modification has on a fundamental physical property (here, a putative “baseline” *T*_m_/*T*_g_) by making a simultaneous modification of opposing effect to the same ion.

We used TGA to gain insight into the thermal stability of the compounds, specifically by analyzing the temperature at 5% mass loss (*T*_onset5%_), and the resulting data are compiled in Table 1 as well. The temperature for this initial decomposition step of the ILs was observed to fall in the range of 142.6–227.2 °C. All the compounds display a complex decomposition profile, wherein multiple weight loss steps are observed (see the Supporting Information for complete TGA traces). For example, compounds **1** and **2** share a nearly identical decomposition profile, wherein a single, large weight loss event is observed. However, examining the derivative thermogravimetry curve reveals a secondary step within this event. Given the similarity in the temperatures for these events, we can conclude that while addition of the chlorine moiety influences the phase transitions, there is no influence on the thermal stability of the compounds. Of particular interest is the first decomposition step for each compound occurring between 140 and 220 °C. Given the similarity of these initial steps, it is likely that this first step is identical for each compound. It could be reasonably assumed, given the other data presented herein, that this primary decomposition arises from C–N bond cleavage, separating the heterocyclic moiety from the trityl group. In this upper range of thermal stability, we see the 4-aminopyridinium-type salts **9**–**12**, indicating that an increase in structural symmetry and a stronger quaternary N–C bond provides higher thermal stability.^29^

## 2.3 X-ray and Computational Analyses

To better understand the observed thermal trends noted in the ILs, we employed the single crystal X-ray crystallographic analysis to gain an insight into the intermolecular interactions and the spatial arrangement of the atoms. The majority of these triarylmethane-based salts were waxy or gel-like solids, thus making the process of growing suitable single crystals a challenging task. Another issue hindering crystal growth is the presence of the sterically demanding trityl groups introducing strain in the N–C bond, making the compounds susceptible to hydrolysis in the timescale necessary for crystal growth. However, crystals of **1** and **9** were successfully grown and analyzed. In addition, the crystallographic data of (2-chlorophenyl)diphenylmethanol (**2a**), the hydrolysis product of **2**, were included. Compound **2a** provides an important insight into the intra and intermolecular interactions present in the triphenylaryl moiety, allowing for a better understanding of the structure of the bound group in the ILs.

**Figure 5:**
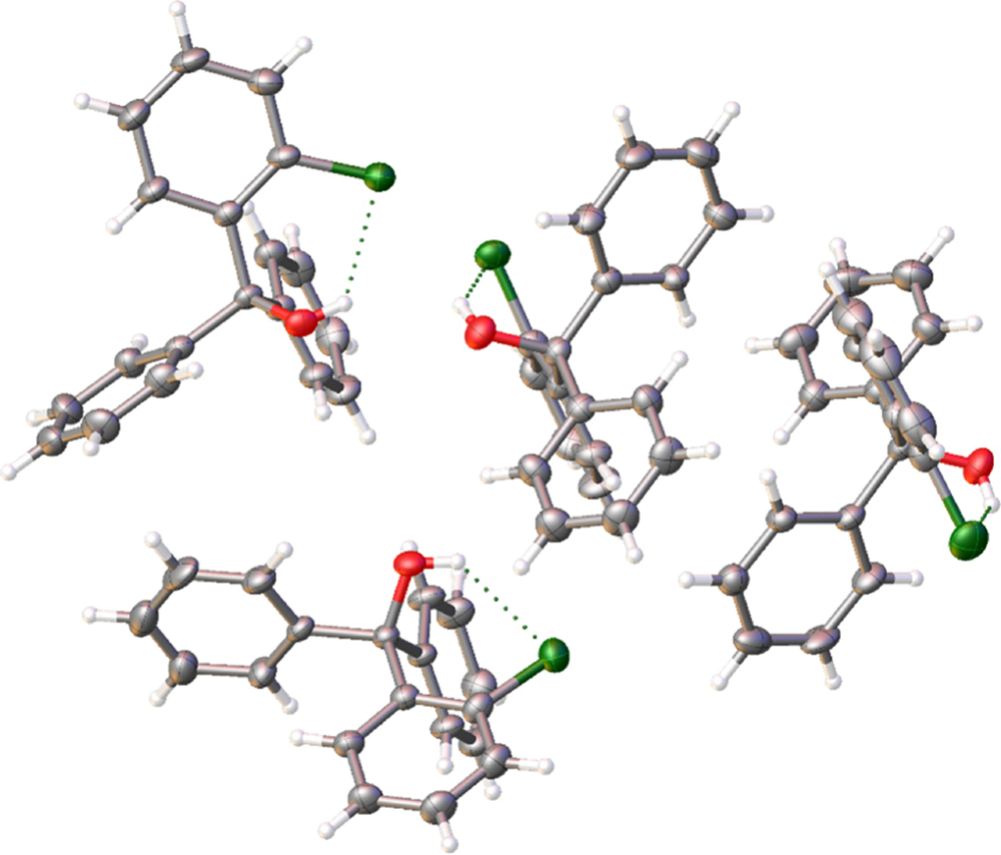
Asymmetric unit of (2-chlorophenyl)diphenylmethanol (**2a**), shown with 50% probability ellipsoids. Intramolecular hydrogen bonding is observed between the central hydroxyl group and the *ortho*- chlorine atom (carbon: gray; hydrogen: white; oxygen: red; chlorine: green).

Compound **2a** crystallizes in the *P*2_1_2_1_2_1_ space group with four individual moieties in the asymmetric unit (Figure 5). While the four individual moieties are crystallographically unique, all four display similar bond distances, lengths, and plane angles. Thus, for the purposes of simplifying the discussion, only moiety “*C*” will be used in the analysis unless otherwise specifically noted. Figure S1 shows the four fingerprint plots for all four moieties.

**Figure 6:**
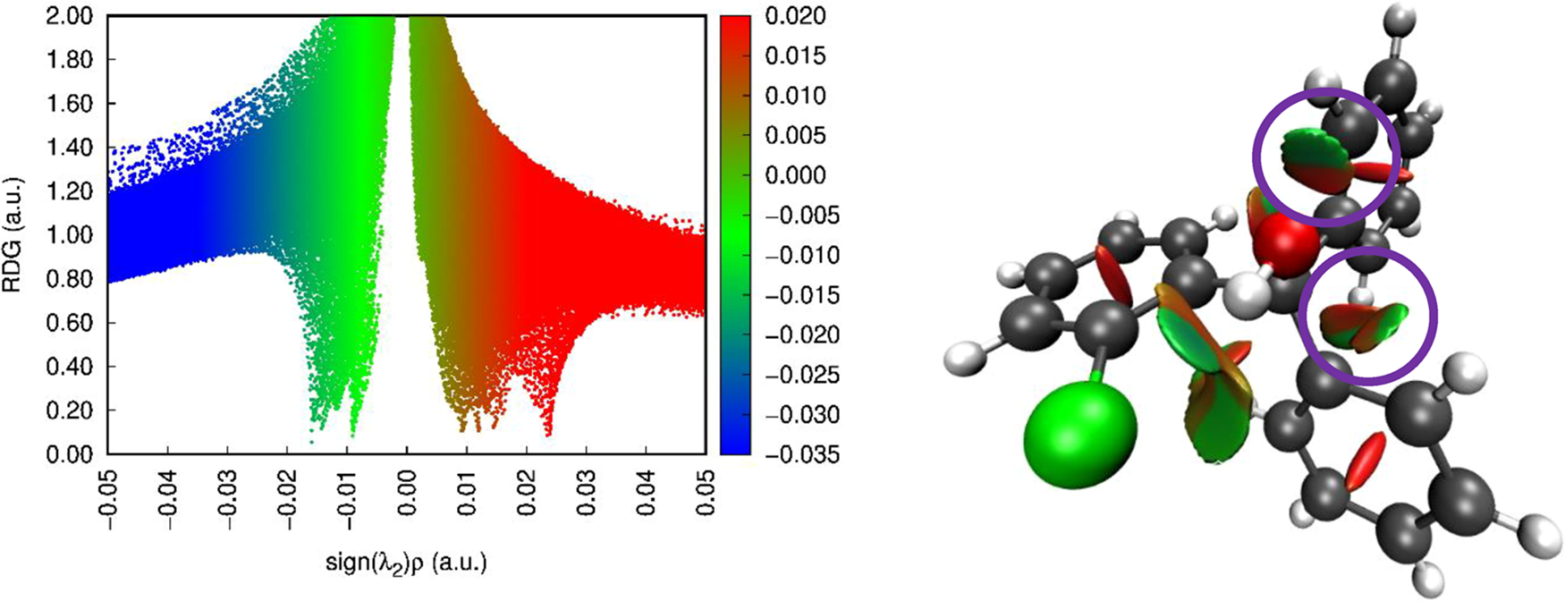
Left: Scatter plot of the reduced density gradient versus sign(λ_2_)*ρ* for moiety C of structure **2a**. Right: Reduced density isosurface for moiety C. Interactions between *ortho* positions in the triarylmethane group are visualized as the green/brown flat surfaces indicated by purple circles.

In an effort to draw out significant intramolecular interactions within the triarylmethane moiety, we completed reduced density gradient (RDG) analysis^35^ on moiety “*C*” of the asymmetric unit. The plots of the RDG vs sign(λ_2_)ρ and the corresponding isosurface are shown in Figure 6. The coloring scheme used denotes the nature of these interactions, wherein blue is indicative of strong noncovalent interactions (e.g., H-bonding), green/tan represents weak van der Waals interactions, and red denotes steric repulsions.^36^

Several salient features are observed in the two images. First, the hydrogen interaction with the chloride atom is seen as the light green/blue surface between the two atoms, indicative of a hydrogen bond. Specifically, there is an O–H···Cl distance of 1.732(6)Å *d*(H···Cl) and an O–H···Cl angle of 134.2°. This H···Cl interaction is observed in all four moieties in the asymmetric unit. Further, Takemura et al. examined the closely related 2-fluoro derivative, which was found to exhibit intramolecular hydrogen O–H···F interactions both in solution and in the crystalline state and believed to orient the angles of the rings of the triarylmethyl scaffold.^37^ Thus, while within the ILs discussed herein the central OH moiety is absent, we can logically infer that the chlorine moiety on the trityl moiety will influence the orientation of the phenyl rings in the bound cationic system.

Second, there are interactions between all the *ortho* hydrogens and the adjacent rings. Both sets of *ortho* hydrogens are seen interacting with adjacent moieties, either the central hydroxyl group or the adjacent aromatic ring. These interactions are visualized as the brown/green surfaces near the hydrogen atoms. Based on the observations with the crystal structure of **1** and from previous studies involving the rotations of substituted triarylmethane groups,^38^ it is likely that the triarylmethane groups attached to the ILs is a rigid group with little rotation in the aromatic rings themselves. Interactions with groups in the ortho positions of the triarylmethane group will have a significant steric impact on the structure by slowing or preventing rotation of the aryl rings.

**Figure 7:**
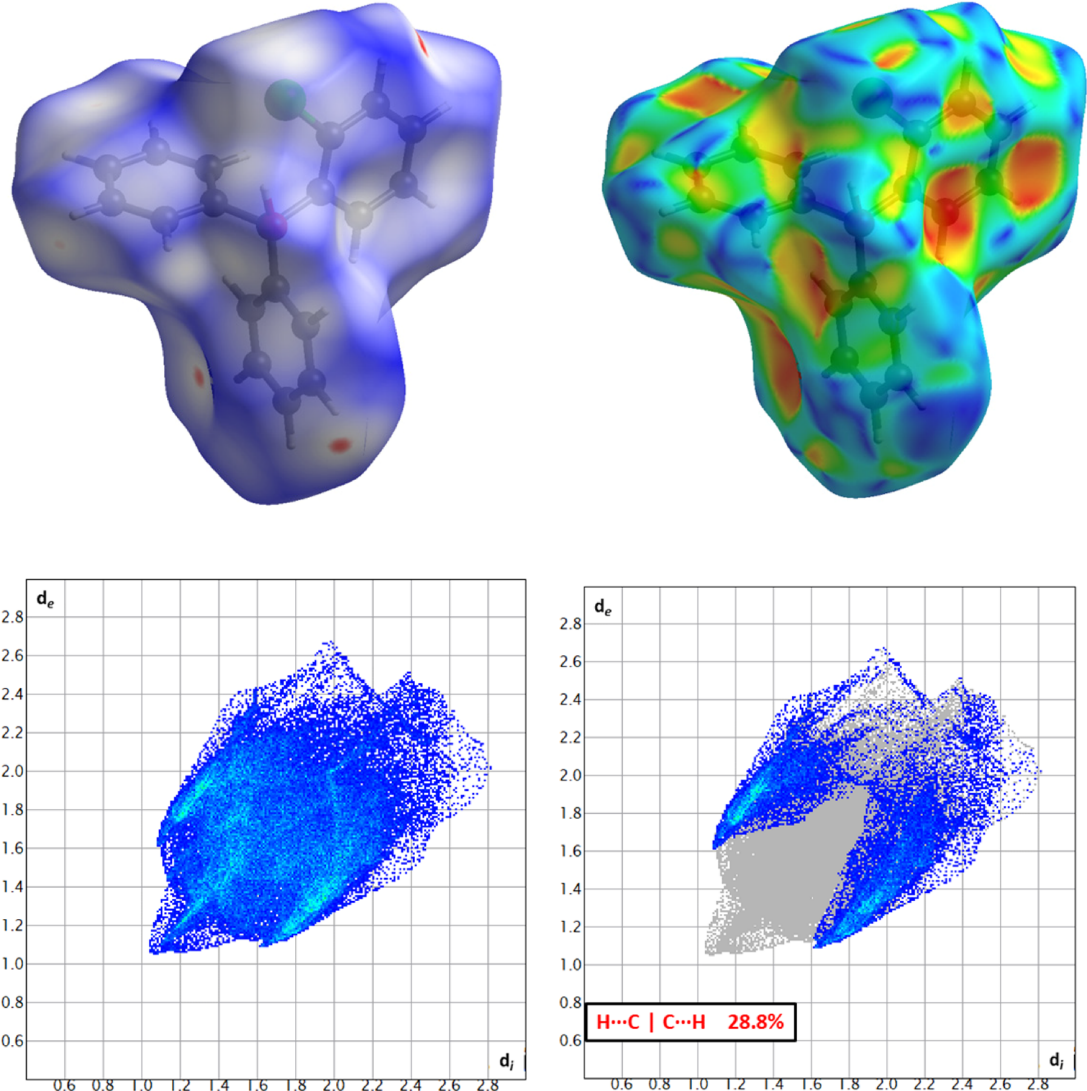
Top left: Moiety C of structure **2a** with the Hirshfeld surfaced mapped with the *d*_norm_ function. Top right: Moiety C of structure **2a** with the Hirshfeld surfaced mapped with the shape index. Bottom left: Complete fingerprint plot for moiety C. Bottom right: Fingerprint plot of moiety C with only the π interactions (H···C|C···H) depicted.

To further examine the impact the triarylmethane groups on intermolecular interactions, the Hirshfeld surface was calculated and analyzed^39−41^ on the individual moieties in the asymmetric unit. Considering that the Hirshfeld surface analysis is a relatively new tool in the structural analysis of low melting organic salts,^42^ we are expanding this concept to our ILs in order to provide a computationally efficient approach to analyze molecular packing, close contact points, molecule shape, and interionic interactions. The calculated surfaces and the corresponding interaction fingerprint^43^ are shown in Figure 7. The fingerprint for **2a** shows several characteristic features indicative of specific noncovalent interactions. For example, on the periphery of the interactions (*d*_i_ ≈ 1.1 Å, *d*_e_ ≈ 1.6 Å, and reciprocal distances) exist two “wing”-type features seen as the areas indicated in Figure 7. These wings are characteristic of interactions with π systems.^44^ For **2a**, these interactions predominantly exist as interactions between aromatic hydrogens and the π clouds of adjacent moieties.

Several of these interactions are shorter in distance when compared with other similar interactions.^45,46^ Of note, these π interactions are seen as the bright red spots on the *d*_norm_ surface over the meta hydrogen on the rings, indicative of interactions shorter than the sum of the van der Waals radii of the atoms. For example, the aromatic hydrogen H16C is interacting with moiety B in the asymmetric unit at a distance of 3.12 Å to the ring center [*d*(H···π)]. These interactions with the π cloud of the aromatic rings are more evident when examining the shape index over the region surrounding the rings, seen as the red concave regions near the center of the rings. While all three rings on moiety “C” display some degree of π interactions, the nature of these interactions is unique for the individual rings. However, given the presence and high percentage (28.8%) of the reciprocal H···C|C···H interactions, it is clear that π–π stacking interactions play an important role in the formation of the long range ordering of the solid-state for the triarylmethane moiety.^47^ Given that these π interactions have been observed in other ILs bearing triphenyl moieties,^48^ it is highly probable that the other compounds herein, **1–12**, exhibit these interactions as well.

**Figure 8:**
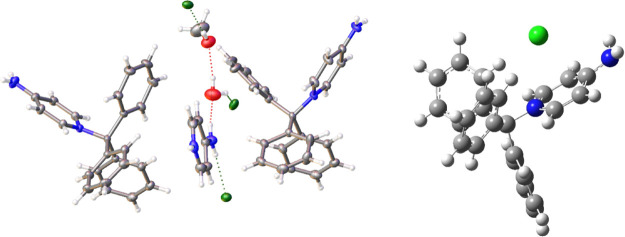
Left: Asymmetric unit of compound **9** shown with 50% probability ellipsoids. Disordered anions and solvate molecules are omitted for clarity. Right: Optimized geometry at M06-2x/6-31++G(d,p) level of theory and basis set (carbon: gray; hydrogen: white; oxygen: red; nitrogen: blue; chlorine: green).

A crystal of the partially hydrolyzed compound **9** was successfully grown (Figure 8). Strikingly, the crystal structure has a protonated 4-aminopyridnium moiety in a layer between two of the IL moieties. Methanol and water molecules are also present in the asymmetric unit. These units, along with the chloride anions, are all linked together through extensive hydrogen bonding. Two discrete moieties of not hydrolized **9** are also present in the asymmetric unit, offering an opportunity to examine the exact nature of the solid-state structure of this novel class of functional IL.

**Figure 9:**
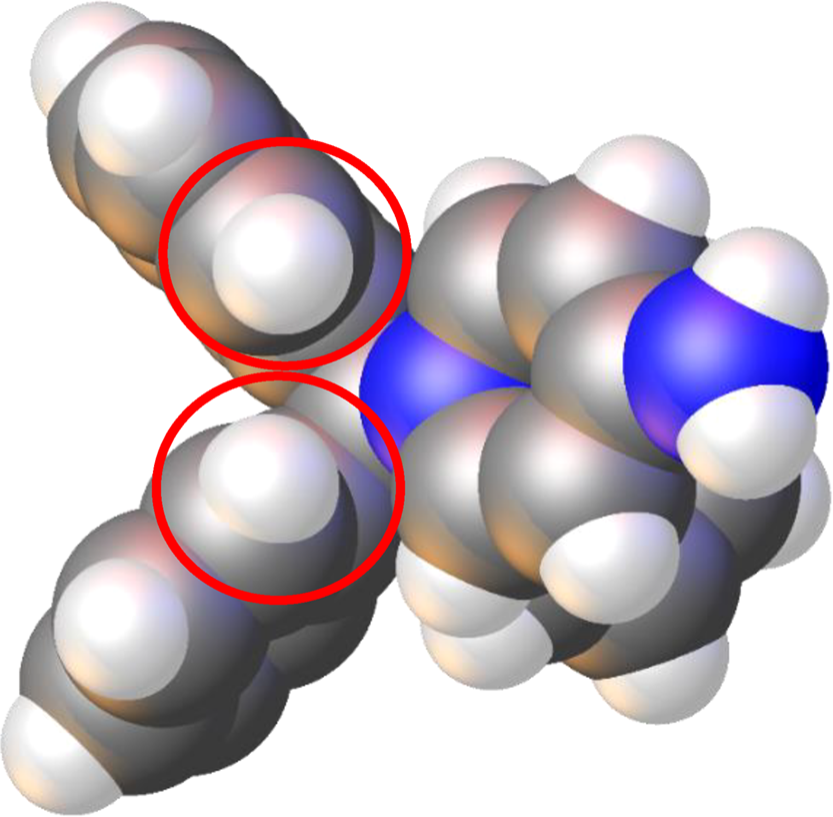
Cation of compound **9** represented as a space-filling model. The red circles highlight the proposed steric interaction with the pyridine moiety causing a bent arrangement.

The 4-aminopyridine moiety of the ILs is bound to the central carbon of the triarylmethane groups through the azaarene nitrogen, as expected. Due to the sterically demanding geometry of the triarylmethane group, the bound pyridinium ring is bent out of the expected linear arrangement (vide infra). A space-filling model of the compound shows that two of the hydrogens on the *ortho* positions on the triarylmethane group are likely causing this bending (Figure 9), which is in agreement with the discussion regarding the RDG analysis of compound **1** (vide supra).

A theoretical validation of this was provided by the optimized structure of compound **9** obtained by performing density functional theory (DFT) simulations (Figures 9 and S2). This bending of the pyridinium–triarylmethane (C–N) bond weakens the bond, making it more susceptible to hydrolysis, as is observed in this case. The experimental bond distance between the central triarylmethane carbon (C6) and the nitrogen (N1) is longer than expected (*d*(C–N) = 1.5238(17) Å) but is in close agreement with 1.516 Å in the simulated structure of IL **9** (Table 3). A search for related C–N bond distances in the CSD^49^ using Mogul^50^ reveals that the bond distance in compound **9** is longer than expected, thus being weaker and offering a rationale to the susceptibility to hydrolysis (the results of the Mogul geometry check are shown in Figure S3). The thermochemical analysis of hydrolysis of compound **9** revealed that the calculated enthalpy of hydrolysis was −12.76 kcal/mol (Table 2). This further confirmed that the hydrolysis of compound **9** is a thermodynamically favored process, which is perhaps driven by the bulky nature of the pyridinium cation and triphenyl moiety that leads to elongation and eventual breaking of the C–N bond as well as formation of a stronger (not bent) C–O bond in the hydrolyzed trityl group.

**Table 2:** Computationally Calculated Free Energies and Enthalpies of Hydrolysis for ILs **1** and **9** Performed at M06-2X/6-31++G(d,p) Theory and Basis Set

IL	Δ*G* (kcal/mol)	Δ*H* (kcal/mol)
**1**	–10.17	–5.13
**9**	–18.03	–12.76

**Figure 10:**
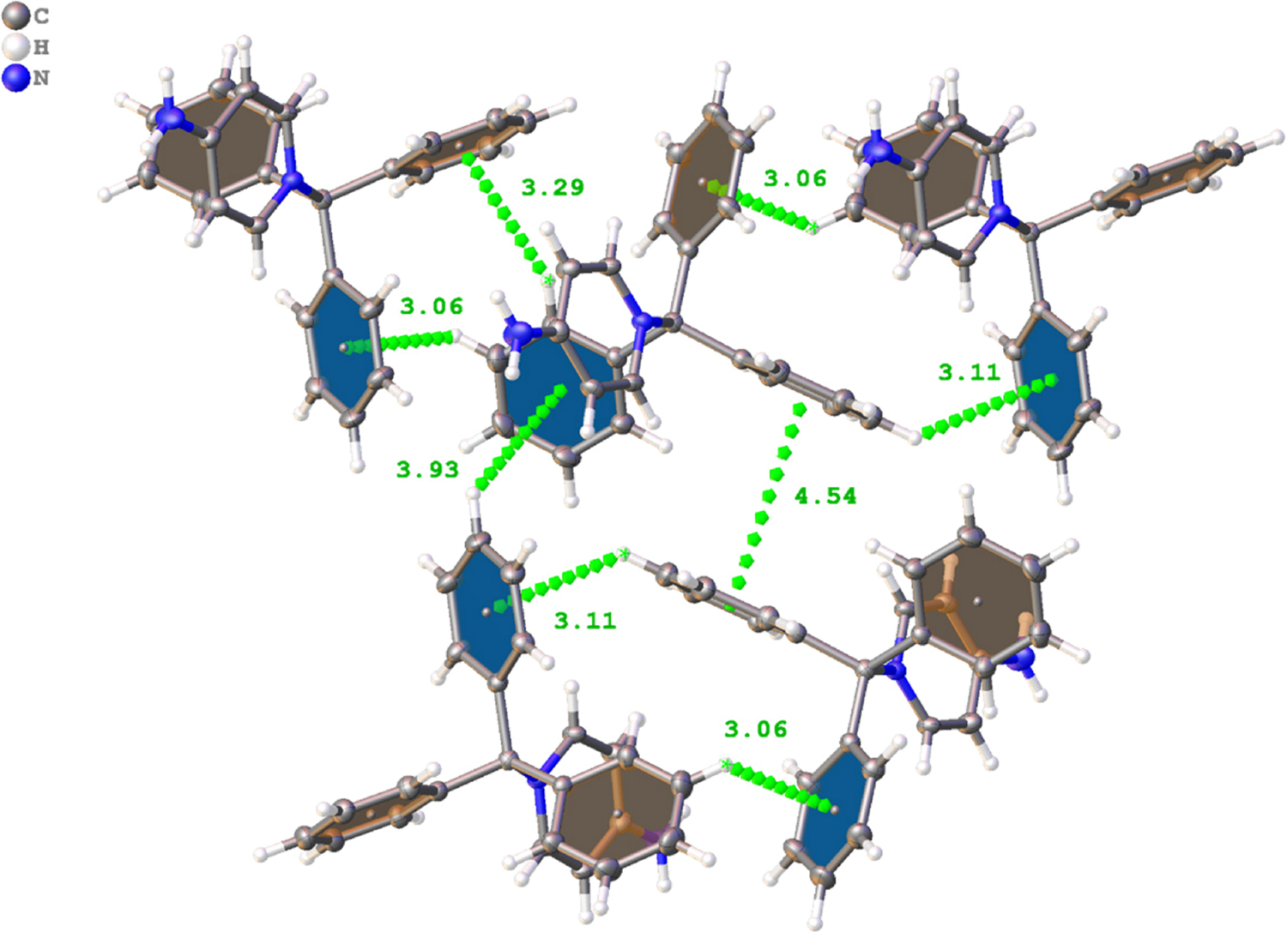
Representations of a portion of the H···π interactions observed between the cations in compound **9**. Distances are shown in angstroms (Å). Blue and orange planes are used to represent the two faces of the aromatic rings.

Compound **9** shares several structural features observed for the triarylmethane groups of structures **1** and **2a**. Specifically, there are numerous π interactions that are linking the cationic groups together in the solid state. All three benzene rings on the triarylmethane group interact with numerous aromatic hydrogens on adjacent rings at distances ranging from 2.85 to 3.24 Å. A depiction of several of these interactions is shown in Figure 10. Additionally, the π system of the pyridinium ring appears to interact with adjacent cation moieties. However, these interactions seem to be artifacts of inefficient packing, given the long distances and angles involved with the interactions. These distances and angles are likely due to the sterically demanding environment surrounding the cationic moiety that prevents close interactions with the π system of the pyridine ring. Instead, the amine group is favoring the formation of hydrogen bonding interactions with the solvent and chloride anions. It should be noted, however, that the chloride anion resides at a distance of 3.975(7) Å [*d*(π···Cl)], appearing to interact with the π system of the cationic ring, though this distance is quite longer than previously reported structures bearing anion···π interactions.^51^

**Figure 11:**
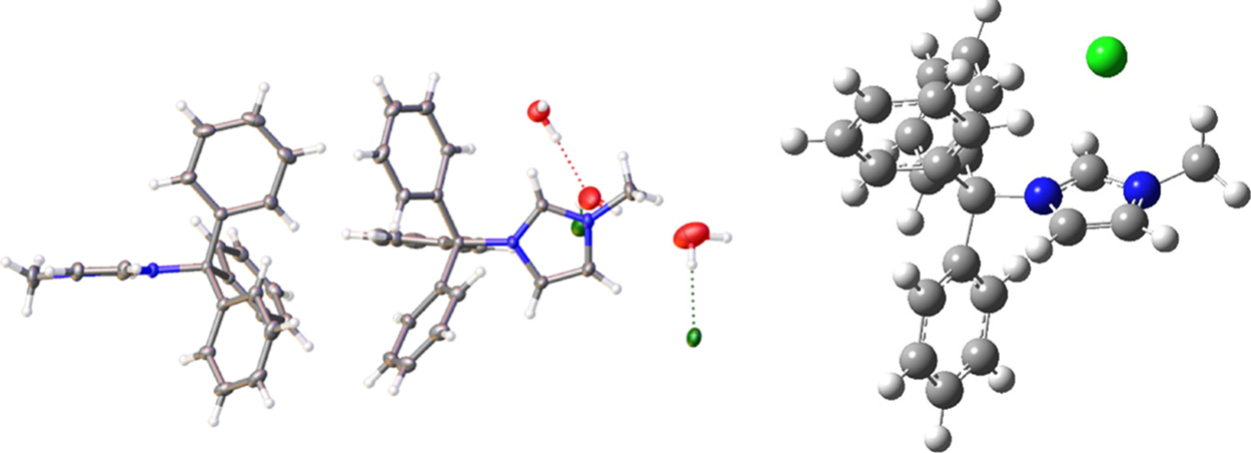
Left: Asymmetric unit of compound **1** shown with 50% probability ellipsoids. Disordered waters are omitted for clarity. Right: Optimized geometry at M06-2x/6-31g++(d,p) level of theory and basis set (carbon: gray; hydrogen: white; oxygen: red; nitrogen: blue; chlorine: green).

The asymmetric unit of the crystal for compound **1** is shown in Figure 11, which contains two cationic moieties along with several disordered water molecules and chloride anions. Likewise, a theoretical validation of this was obtained by the optimized structure of compound **1** via performing DFT simulations (Figures 11 and S4). Given the heavily disordered nature of the anions and water molecules, surface analysis of the compound proved to be ambiguous. However, drawing from the analysis of the other structures discussed herein (i.e., **2a** and **9**) proves to be useful in discerning relevant interactions present in **1**. The imidazolium ring in structure **1** is linear with respect to the central triarylmethane carbon group, in contrast with the bent pyridinium ring observed in **9**. The reduced strain on the C–N bond arises from the smaller five-membered imidazolium ring in **1** compared to the six-membered pyridinium ring in **9**, which is sterically more demanding. The bond length between imidazolium nitrogen and triarylmethane carbon is 1.4957(13)Å *d*(C–N), which is nearly identical to the computed bond length of 1.498 Å (Table 3). This C–N distance in compound **1** is significantly shorter compared to the C–N bond of 1.5238(17) Å in compound **9**. The shorter bond length in is an indicator of relatively smaller strain on the C–N bond; as a result, compound **1** did not undergo hydrolysis in the presence of water. Moreover, the thermochemical analysis revealed that the Δ*H* of hydrolysis for **1** is lower than **9** (see Table 2) with ΔΔ*H* = −7.63 kcal/mol, which is indicative of a higher susceptibility of **9** to undergo hydrolysis compared to **1**.

**Table 3:** Comparison between Experimentally Observed and Computed C–N Bond Lengths for ILs **1** and **9**

IL	experimental	computed
**1**	1.496	1.498
**9**	1.524	1.516

**Figure 12:**
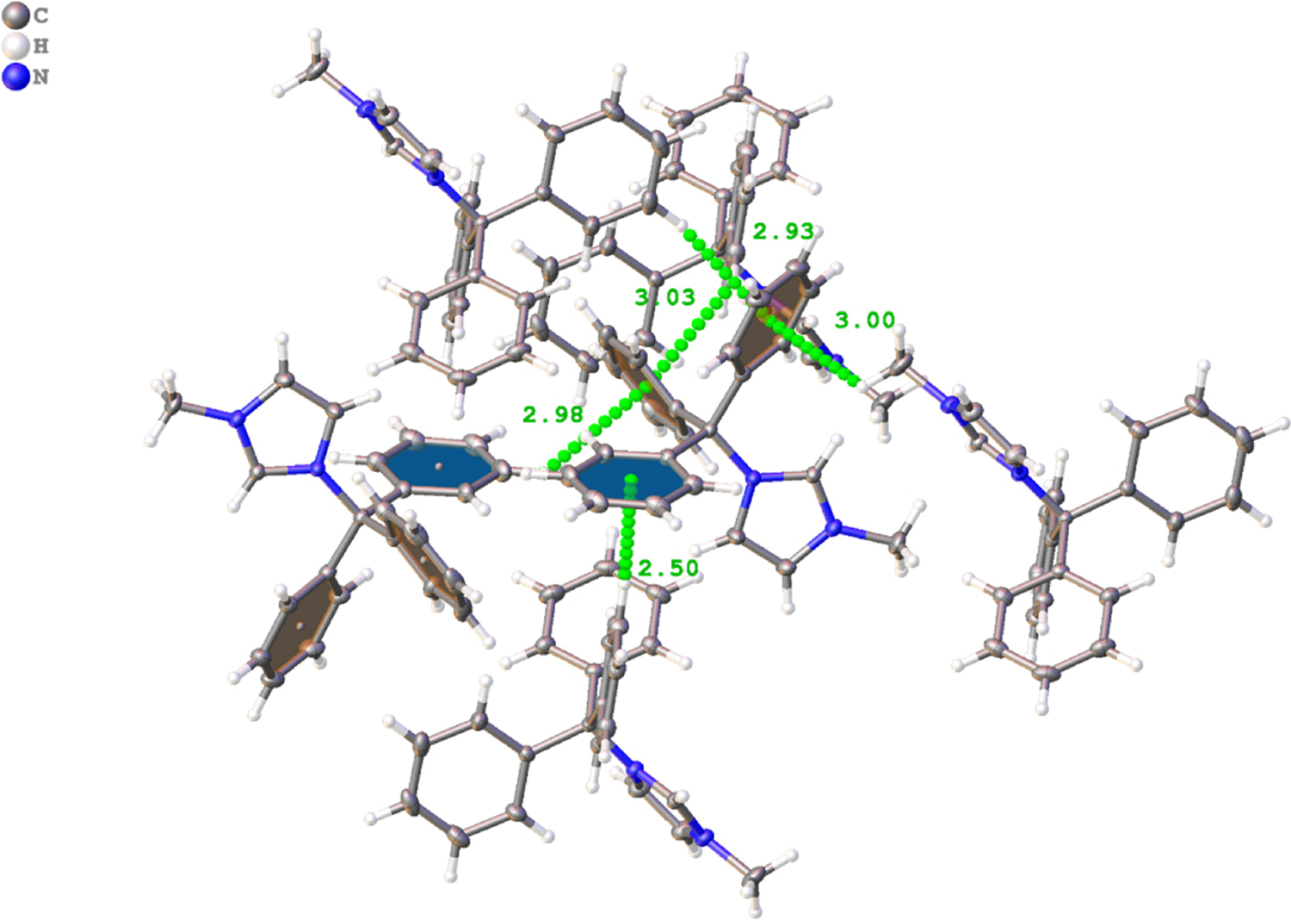
Representations of a portion of the H···π interactions observed between the cations in compound **1**. Distances are shown in angstroms (Å). Blue and orange planes are used to represent the two faces of the aromatic rings.

All three of the benzene rings in both moieties of **1** in the asymmetric unit are interacting via C–H···π interactions with adjacent cations (Figure 12). These interactions vary from ring to ring, however, typically falling within the range of 2.50 to 3.00 Å. A few key distinctions are observed when comparing **1** and **9**. For example, the methyl protons in the imidazolium ring of **1** have linear intermolecular C–H···π interactions with adjacent molecules compared to the aromatic hydrogens’ moieties in **9**. Simply put, the methyl protons in **1** interact with the center of the aromatic ring and are perpendicular to the plane of the ring, whereas, in contrast, the aromatic C–H protons in **9** are skewed at an angle relative to the plane of the ring. Further, the methyl group on the imidazolium ring interacts with the triarylmethane π system, which is absent in compound **9**. The importance of C–H···π interactions in the crystallizability of the resulting IL is also supported in noting the amorphous nature and inability to crystallize the compounds containing 4-methoxy substituents on the aryl rings (**4**–**6**, **9**, and **10**). Many C–H···π interactions in the analyzed crystal structures occur with the *para* positioned hydrogen; thus, we can infer that this key bonding network is disrupted by 4-methoxy functionalization, resulting in amorphous compounds featuring glass transitions. However, it should be explicitly stated that while we logically deduce the importance of the C–H···π interactions to the formation of the crystals for these systems, coulombic interactions will still be the dominant force in crystal formation.^52^

The imidazolium ring also shows anion···π interactions with the chloride anions. Several N···Cl interactions are observed, wherein the chloride atoms are interacting with the π system of the aromatic ring at distances of approximately 3.60 Å. It should be noted that water molecules are also interacting with the imidazolium ring at these distances, though given the disordered nature of these solvents, it is difficult to discern any contributions from these water interactions. However, both anions and water molecules are known to interact with π systems of aromatic rings.^53^

In order to validate the simulated data and the predicted physical properties of a system, it is important to compare it with the experimental observations. We compared computed molecular geometries of compounds **1** and **9** with the molecular structures obtained using single crystal X-ray crystallography. Significantly, we observed that the interatomic bond distances predicted using M06-2X/6-31G++(d,p) theory and basis set were in close agreement with the single crystal data (see Figure S5 and Tables S2 and S3), indicating that the M06-2X/6-31G++(d,p) theory and basis set are able to accurately predict molecular structure and their subsequent physical properties.

## 3 Experimental Section

The detailed synthetic procedure and the crystallographic and computational methods used in this work are described in the Supporting Information.

## 4 Conclusions

This endeavor represents the first introduction of the triarylmethyl moiety to ILs as a biologically compatible model to develop functional ILs with a priori reduced toxicity. Through a facile approach, we developed a wide variety of ionic materials bearing the pharmaceutically significant triarylmethyl moiety and characterized several notable trends in physicochemical properties to inform and expand understanding of IL design principles. The trityl motif dominates the steric and conformational properties of the ILs. Further, unique structural substituents affect the properties and chemical behavior of the resulting IL. The modifications of the aryl rings and the cation head group change the dipole moments of the cations, leading to a clear pattern in melting points or phase transitions in the liquid state. This is especially true in the case of incorporation of asymmetrical substituents (e.g., *ortho*-Cl). The electron-donating or -withdrawing properties and position of these substitutions alter the orientation of the dipole moment. This in turn changes the intermolecular interactions that influence the phase transition behavior of ILs.

Further insights provided through SC-XRD data paired with Hirshfeld surface analysis and RDG analysis revealed features such as key interactions and spatial orientation of each structural element that rationalized the thermophysical patterns observed. Both intra- and intermolecular interactions, particularly with the π system of the aryl rings, play a defined role in the formation of crystalline vs amorphous solids, not understating the role of coulombic interactions. The theoretical studies revealed results consistent with the SC-XRD data in terms of key bond lengths, allowing for analysis of the observed solid-state behavior of the ILs. Specifically, the lengthening and shortening of the heterocyclic cation–trityl bond is directly correlated with susceptibility to hydrolysis. The calculated dipole moment and thermochemical values further establish the links to structure or chemical substituents.

## Supporting Information Available

Synthesis and characterizations of the ILs: ^1^H, ^13^C NMR, and MS spectroscopy; DSC and TGA thermographs; Check CIF files; and additional theoretical and crystallographic data (PDF)
Crystal data of samples **1**, **2a**, and **9** (CIF) (CIF) (CIF)

## Supplementary Material

pg2c00048_si_001.pdf

pg2c00048_si_002.cif

pg2c00048_si_003.cif

pg2c00048_si_004.cif

## Dedication

This article is dedicated to the memory of Professor David A. Evans (1941-2022), an inspirational chemist and educator.
