# Supplementary material for: Anticancer Agents as Design Archetypes: Insights into the Structure–Property Relationships of Ionic Liquids with a Triarylmethyl Moiety
Source: ACS Phys Chem Au. 2022 Dec 7;3(1):94–106. doi: 10.1021/acsphyschemau.2c00048 (PMC9881241; doi:10.1021/acsphyschemau.2c00048)
Supplement: Supplementary file 1 — pg2c00048_si_001.pdf [file pg2c00048_si_001.pdf]

Supporting Information

Anticancer Agents as Design Archetypes: Insights into the Structure–Property Relationships of Ionic Liquids with a Triarylmethyl Moiety

Grace I. Anderson,[†] David Hardy,[†] Patrick C. Hillesheim,*[,‡] Durgesh V. Wagle,*[,†] Matthias Zeller,[‖] Gary A. Baker,[⊥] and Arsalan Mirjafari*[,§]

[†]Department of Chemistry and Physics, Florida Gulf Coast University, Fort Myers, Florida 33965, United States

[‡]Department of Chemistry and Physics, Ave Maria University, Ave Maria, Florida 34142, United States

[‖]Department of Chemistry, Purdue University, West Lafayette, Indiana 47907, United States

[⊥]Department of Chemistry, University of Missouri-Columbia, Columbia, Missouri 65211, United States

[§]Department of Chemistry, State University of New York at Oswego, Oswego, New York 13126, United States

Emails: arsalan.mirjafari@oswego.edu (A.M.), patrick.hillesheim@avemaria.edu (P.C.H.), dwagle@fgcu.edu (D.V.W.)

EXPERIMENTAL

Materials and Instrumentation. All commercial chemicals are used as received unless otherwise noted. H^1 and C^{13} NMR spectra were performed on a JEOL 400 MHz NMR at 295 K with the chemical shifts (δ) notated as parts per million (ppm) and referenced to the corresponding residual NMR solvent peaks ($CDCl_3$, DMSO-d_6). ESI-MS analyses were performed on an Agilent Technologies Infinity Labs LC/MSD model G6125B equipped with electrospray ionization (ESI) and data was collected in positive ion mode. Melting points and glass transition temperatures were measured using a TA Discovery 250 DSC Differential Scanning Calorimeter, calibrated using indium (melting point) and sapphire (heat capacity) references. Thermogravimetric analyses were performed on a TA instrument TGA 550 under nitrogen flow using a platinum pan. The samples were heated from room temperature at a rate of 25 ºC/min to a maximum temperature of 600 ºC. Single crystal XRD experiments were carried out with a Bruker AXS D8 Quest diffractometer with a PhotonII charge-integrating pixel array detector (CPAD) and a Cu-*Kα* radiation microsource X-ray tube. Absorption was corrected for by multi-scan methods using *SADABS*. Additional details are provided in the Crystallographic Data (*vide infra*).

Differential Scanning Calorimetry. In this work, melting points are reported as the transition from crystalline solid state to the isotropic liquid state, distinguished by the magnitude of enthalpy for the transition and the shape of the DSC curve. For each experiment, 5−15 mg of the sample was loaded into an open aluminum pan and heated to 150 °C for 20–30 min to remove a water absorbed from the environment, residual solvents, or volatile contaminants from synthesis. The samples were then cooled to −50 °C, equilibrated for 2 min and then heated at a ramp rate of 5 °C/min to 200 °C. Determined by the TRIOS analysis software, melting points were reported as

the melting onset temperature and glass transition temperatures are reported midpoints of phase transitions. The samples underwent 8-10 heating and subsequent cooling process at a rate of 5 ºC/min, alternating with 10 min isothermal periods to identify the correct phase transitions by observing three overlapping cycles and reported values were the average of the at least three measurements. All measurements were carried out under a nitrogen atmosphere (50 mL/min) and were reproducible to within ±1 °C.

Synthetic Procedure. IL **1** was synthesized by placing of 1-methylimidazole **1a** (1 g, 1 equiv.) in a single-necked 100mL round-bottom flask, equipped with an egg-shaped Teflon-coated magnetic stir bar and dissolving it in 5 mL of dry ethyl acetate. In a separate 50mL beaker, the trityl chloride **1b** (3.4 g, 1 equiv.) was also dissolved in 5 mL of dry ethyl acetate and was added to the 1-methylimidazole solution in one portion while being magnetically stirred. The solution was vigorously stirred at room temperature and a solid precipitate was formed within 2 hours (Scheme S1). The precipitate was separated via vacuum filtration through a sintered glass Buchner funnel and filter cakes were washed with dry ethyl acetate (3 × 10 mL). The product **1** was obtained as a white solid in 92% yield, which was recrystallized via vapor-diffusion method using methanol as the solvent and diethyl ether as the anti-solvent for the crystallographic studies.

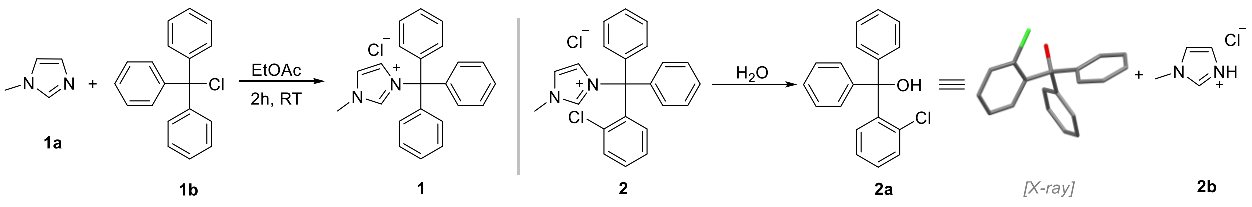

Scheme S1. Left: one step synthesis of trityl-based IL **1**. Right: aerobic hydrolysis of IL **2**, forming the trityl alcohol **2a**.

The same synthetic method was utilized to prepare ILs **3, 7–12** (for the instant formation of IL **12**, see: https://youtube.com/shorts/jywjFBoWens). IL **9** was recrystallized via vapor-diffusion method using methol as the solvent and methyl *t*-butyl ether as the anti-solvent. ILs **4**, **5**, and **6** were synthesized with this modified procedure, replacing acetonitrile as the solvent instead of ethyl acetate. IL **2** was synthesized with a different modification to the original method, replacing the solvent with toluene and stirring at 50 °C for 3 days. The resulting precipitation was recovered via vacuum filtration and washed with (3 × 10 mL) of tetrahydrofuran to obtain the product as a white solid in 86% yield.

IL **1**. ^{1}H NMR (400 MHz, $CDCl_3$) δ_H 9.39 (s, 1H), 7.37-7.24 (m, 18H), 7.09 (s, 1H), 4.04 (s, 3H); ^{13}C NMR (101 MHz, $CDCl_3$) δ_C 146.8, 127.9, 127.3, 121.6, 120.2, 82.0, 36.1; MS (ESI): *m/z* 325.1 (M = $C_{23}H_{21}N_2^+$, calcd. 325.2).

IL **2**. ^{1}H NMR (400 MHz, $CDCl_3$) δ_H 9.75 (s, 1H), 7.77 (s, 1H), 7.50-6.93 (m, 15H), 4.31 (s, 3H); ^{13}C NMR (101 MHz, $CDCl_3$) δ_C 139.1, 138.3, 137.5, 137.0, 132.9, 131.6, 131.4, 129.9, 129.5, 129.1, 127.9, 123.5, 123.4, 79.3, 37.8; MS (ESI): *m/z* 359.2 (M = $C_{23}H_{20}ClN_2^+$, calcd. 359.1).

IL **3**. ^{1}H NMR (400 MHz, DMSO-d_6) δ_H 8.11 (d, J = 9.4 Hz, 2H), 7.43-7.04 (m, 15H), 3.90 (s, 3H); ^{13}C NMR (101 MHz, DMSO-d_6) δ_c 146.6, 144.7, 134.0, 132.9, 131.9, 131.0, 129.6, 128.2, 128.1, 127.2, 126.8, 81.3, 35.3; MS (ESI): *m/z* 404.5 (M = $C_{23}H_{19}ClN_3O_2^+$, calcd. 404.1).

IL **4**. ^{1}H NMR (400 MHz, $CDCl_3$) δ_H 9.65 (s, 1H), 7.42-7.20 (m, 11H), 6.90-6.87 (m, 4H), 4.12 (s, 3H), 3.85 (s, 6H); ^{13}C NMR (101 MHz, $CDCl_3$) δ_c 158.7, 147.4, 139.5, 136.1, 129.2, 127.9, 127.8, 127.2, 121.9, 121.4, 113.2, 81.5, 55.3, 31.0; MS (ESI): *m/z* 385.9 (M = $C_{25}H_{25}N_2O_2^+$, calcd. 385.2).

IL **5**. ^{1}H NMR (400 MHz, $CDCl_3$) δ_H 9.64 (s, 1H), 7.52-7.28 (m, 8H), 7.10-6.95 (m, 6H), 4.21 (s, 3H), 4.04-3.94 (m, 9H); ^{13}C NMR (101 MHz, $CDCl_3$) δ_c 158.5, 139.6, 135.5, 129.0, 121.5, 120.8, 113.1, 81.1, 55.2, 35.9; MS (ESI): *m/z* 385.8 (M = $C_{26}H_{27}N_2O_3^+$, calcd. 415.2).

IL **6**. ^{1}H NMR (400 MHz, $CDCl_3$) δ_H 7.25-6.80 (m, 14H), 3.78 (s, 9H), 2.79 (s, 3H); ^{13}C NMR (101 MHz, $CDCl_3$) δ_c 158.6, 139.7, 129.1, 121.3, 119.0, 113.2, 81.2, 55.3, 10.7; MS (ESI): *m/z* 329.9 (M = $C_{27}H_{29}N_2O_3^+$, calcd. 429.2).

IL **7**. ^{1}H NMR (400 MHz, DMSO-d_6) δ_H 7.39-7.13 (m, 15H), 7.06 (d, J = 2.1 Hz, 1H), 6.87 (d, J = 2.1 Hz, 1H), 3.42 (s, 3H); ^{13}C NMR (101 MHz, DMSO-d_6) δ_c 148.3, 129.6, 128.3, 128.1, 127.2, 81.1, 34.4; MS (ESI): *m/z* 357.7 (M = $C_{23}H_{21}N_2S^+$, calcd. 357.1).

IL **8**. ^{1}H NMR (400 MHz, DMSO-d_6) δ_H 7.39-7.13 7.37-7.02 (m, 15H), 3.44 (s, 3H); ^{13}C NMR (101 MHz, DMSO-d_6) δ_c 205.3, 146.6, 144.6, 134.1, 131.9, 131.0, 129.6, 128.2, 128.1, 127.2, 126.8, 81.3, 34.5; MS (ESI): *m/z* 391.5 (M = $C_{23}H_{20}ClN_2S^+$, calcd. 391.1).

IL **9**. ^{1}H NMR (400 MHz, DMSO-d_6) δ_H 8.51 (d, J = 13.0 Hz, 1H), 8.06 (dd, J = 32.6, 7.1 Hz, 4H), 7.48-7.39 (m, 4H), 7.26-7.24 (m, 3H), 7.03 (s, 3H), 6.82 (q, J = 3.7 Hz, 1H), 6.61 (d, J = 5.0 Hz, 3H), 3.35 (s, 2H); ^{13}C NMR (101 MHz, DMSO-d_6) δ_c 158.8, 157.7, 143.4, 143.1, 138.4, 137.5, 134.5, 132.7, 131.3, 130.0, 128.6, 127.9, 108.8, 82.5; MS (ESI): *m/z* 337.6 (M = $C_{24}H_{21}N_2^+$, calcd. 337.2).

IL **10**. ^{1}H NMR (400 MHz, DMSO-d_6) δ_H 8.51 8.68 (d, J = 14.4 Hz, 1H), 8.05-8.02 (m, 3H), 7.60-7.09 (m, 10H), 6.87 (d, J = 7.8 Hz, 1H), 6.68-6.66 (m, 2H), 3.50 (s, 2H); ^{13}C NMR (101 MHz, DMSO-d_6) δ_c 158.8, 157.7, 143.4, 143.1, 138.4, 137.5, 134.5, 132.7, 131.3, 130.0, 128.6, 127.9, 109.0, 108.8, 82.5; MS (ESI): *m/z* 371.5 (M = $C_{24}H_{20}ClN_2^+$, calcd. 371.1).

IL **11**. ^{1}H NMR (400 MHz, DMSO-d_6) δ_H 8.13-8.08 (m, 4H), 7.29-6.81 (m, 13H), 6.22 (s, 1H), 3.77-3.72 (m, 6H); ^{13}C NMR (101 MHz, DMSO-d_6) δ_c 159.8, 157.8, 148.3, 140.2, 139.7, 128.9,

127.6, 127.4, 126.4, 112.7, 108.7, 79.9, 55.0; MS (ESI): *m/z* 397.5 (M = $C_{26}H_{25}N_2O_2^+$, calcd. 397.2).

IL **12**. ^{1}H NMR (400 MHz, DMSO-d_6) δ_H 7.70-7.00 (m, 14H), 6.76-6.70 (m, 3H), 5.56 (s, 2H); ^{13}C NMR (101 MHz, DMSO-d_6) δ_c 213.0, 201.1, 191.0, 189.3, 148.5, 140.4, 139.9, 136.5, 132.0, 130.9, 129.6, 129.0, 128.7, 128.3, 127.9, 120.6, 106.5, 70.5; MS (ESI): *m/z* 352.4 (M = $C_{24}H_{22}N_3^+$, calcd. 352.2).

COMPUTATIONAL METHODS

The simulations were carried out using the Gaussian 16 software package[1] and processed by using Gaussview.[2] The optimization of the starting structures of the salts in the system were carried out by using keywords "opt" and "freq" respectively. Following starting orientations of IL cation–anion clusters were used for optimization (see Figures S1 and S2). The optimization of these ILs was performed at M06-2X/6-31++G(d,p) level developed by Thrular *et. al.,* with no symmetry restrictions in singlet ground state.[3] The Boys and Bernadi counterpoise procedure were used to account for basis set superposition error (BSSE) using the keyword counterpoise=cp embedded in the Gaussian 16 software package.[4] The absence of imaginary frequencies in the computed structures indicated that the optimized structures were a minimum. The dipole moment (D) for the salt was obtained from the optimized structures.

Thermochemical values for the hydrolysis of the salt complex were obtained using the following equation:

$$\Delta X_{Hydrolysis} = \Sigma X_{products} - \Sigma X_{reactants}$$

CRYSTALLOGRAPHIC DATA

Single crystal data of samples **1**, **2a**, and **9** were collected on a Bruker Quest diffractometer with kappa geometry, a Cu Kα wavelength (λ = 1.54178 Å) I-μ-S microsource X-ray tube, laterally graded multilayer (Goebel) mirror single crystal for monochromatization, a Photon II area detector and an Oxford Cryosystems low temperature device. Examination and data collection were performed at 150 K. Data was collected, reflections were indexed and processed, and the files scaled and corrected for absorption using SADABS[5] and APEX3.[6] The space group was assigned using XPREP within the SHELXTL suite of programs,[7,8] the structure was solved by direct methods using ShelXS[8] and refined by full matrix least squares against F^2 with all reflections with Shelxl2018[9] using the graphical interfaces Shelxle.[10] H atoms were positioned geometrically and constrained to ride on their parent atoms. C–H bond distances were constrained to 0.95 Å for alkene C–H moieties, and to 0.99 and 0.98 Å for aliphatic CH_2 and CH_3 moieties, respectively. Methyl H atoms were allowed to rotate but not to tip to best fit the experimental electron density.

$U_{iso}(H)$ values were set to a multiple of $U_{eq}(C)$ with 1.5 for CH_3 and 1.2 for C-H and CH_2 units, respectively. In the structure of compound **9**, one of two chloride anions is disordered with water molecules over two positions. The disorder is correlated with disorder of two other water molecules around a crystallographic inversion center, imposing exact 1:1 disorder (positions of O2 and its symmetry equivalent are mutually exclusive). Uij components of ADPs of O and Cl atoms sharing the same site were restrained to be similar. Water H atom positions were refined and O–H and H...H distances were restrained to 0.84(2) and 1.36(2) Å, respectively, while a damping factor was applied. Some water H atom positions were further restrained based on hydrogen bonding considerations. H atoms attached to disordered water molecules O3, O4 and O6 were in the final refinement cycles set to ride on their carrier atoms and the damping factor was removed.

Complete crystallographic data, in CIF format, was deposited with the Cambridge Crystallographic Data Centre. CCDC numbers 2205099, 2205100, and 2205101, containing the supplementary crystallographic data for this paper. These data can be obtained free of charge from The Cambridge Crystallographic Data Centre via www.ccdc.cam.ac.uk/data_request/cif.

Hirshfeld surfaces, their corresponding images, and fingerprint plots were calculated using the *CrystalExplorer21*.[11] This software explores packing modes and intermolecular interaction by generating a unique Other images and bond distances were analyzed using Olex2[12] and Mercury.[13] Reduced density gradient (RDG) analysis was accomplished using the Multiwfn software.[14] Visualization of the results was accomplished using VMD.[15] The input wavefunction files for the analysis were acquired using ORCA[16] employing the ωB97X functional[17] and the x2c-TZVP basis set in conjunction with the NoSpherA2[18] software as implemented in Olex2. The wavefunction was calculated for only moiety C in the asymmetric unit. The RDG isosurface was plotted with a 0.55 isovalue to best visualize the interactions discussed.

Table S1. Crystal data and structure refinement for compounds **1, 2a,** and **9**.

**Compounds**	**1**	**2a**	**9**
Crystal data			
Chemical formula	$4(C_{23}H_{21}N_2)\cdot 4(Cl)\cdot 7(H_2O)$	$C_{19}H_{15}ClO$	$2(C_{24}H_{21}N_2)\cdot C_5H_7N_2\cdot CH_4O\cdot 3(Cl)\cdot H_2O$
M_r	1569.58	294.76	926.39
Crystal system, space group	Triclinic, *P*1	Orthorhombic, $P2_12_12_1$	Orthorhombic, *Pbca*
Temperature (K)	150	150	150
a, *b*, *c* (Å)	11.9138 (5), 12.9386 (5), 14.5793 (6)	8.4708 (13), 19.356 (4), 36.259 (8)	17.6483 (10), 14.0704 (6), 38.934 (2)
α, β, γ (°)	86.1777 (16), 77.9486 (17), 69.0609 (15)	90, 90, 90	90, 90, 90
V (Å^3)	2052.62 (15)	5945 (2)	9668.0 (9)
Z	1	16	8
Radiation type	Mo *K*α	Cu *K*α	Mo *K*α
μ (mm^{-1})	0.21	2.22	0.24
Crystal size (mm)	0.53 × 0.51 × 0.11	0.24 × 0.08 × 0.03	0.55 × 0.23 × 0.21
Data Collection			
Diffractometer	Bruker AXS D8 Quest diffractometer with PhotonII charge-integrating pixel array detector (CPAD)	Bruker AXS D8 Quest diffractometer with PhotonIII_C14 charge-integrating pixel array detector (CPAD)	Bruker AXS D8 Quest diffractometer with PhotonII charge-integrating pixel array detector (CPAD)
Absorption correction	Multi-scan *SADABS* 2016/2: Krause, L., Herbst-Irmer, R., Sheldrick G.M. & Stalke D., J. Appl. Cryst. 48 (2015) 3-10	Multi-scan *SADABS* 2016/2: Krause, L., Herbst-Irmer, R., Sheldrick G.M. & Stalke D., J. Appl. Cryst. 48 (2015) 3-10	Multi-scan *SADABS* 2016/2: Krause, L., Herbst-Irmer, R., Sheldrick G.M. & Stalke D., J. Appl. Cryst. 48 (2015) 3-10
T_{min}, T_{max}	0.715, 0.747	0.571, 0.754	0.663, 0.747
No. of measured, independent and observed [$I > 2\sigma(I)$] reflections	173297, 15670, 12936	28533, 11591, 9934	97195, 15376, 11162
R_{int}	0.044	0.050	0.074
$(\sin \theta/\lambda)_{max}$ (Å^{-1})	0.770	0.638	0.770
Refinement			
$R[F^2 > 2\sigma(F^2)]$, $wR(F^2)$, S	0.041, 0.117, 1.01	0.057, 0.156, 1.03	0.048, 0.125, 1.02
No. of reflections	15670	11591	15376
No. of parameters	555	762	604
No. of restraints	34	-	2
H-atom treatment	H atoms treated by a mixture of independent and constrained refinement	H-atom parameters constrained	H atoms treated by a mixture of independent and constrained refinement
$\Delta\rho_{max}$, $\Delta\rho_{min}$ (e Å^{-3})	0.46, −0.43	0.77, −0.39	0.49, −0.48
Absolute structure	-	Refined as an inversion twin.	-
Absolute structure parameter	-	0.098 (19)	-

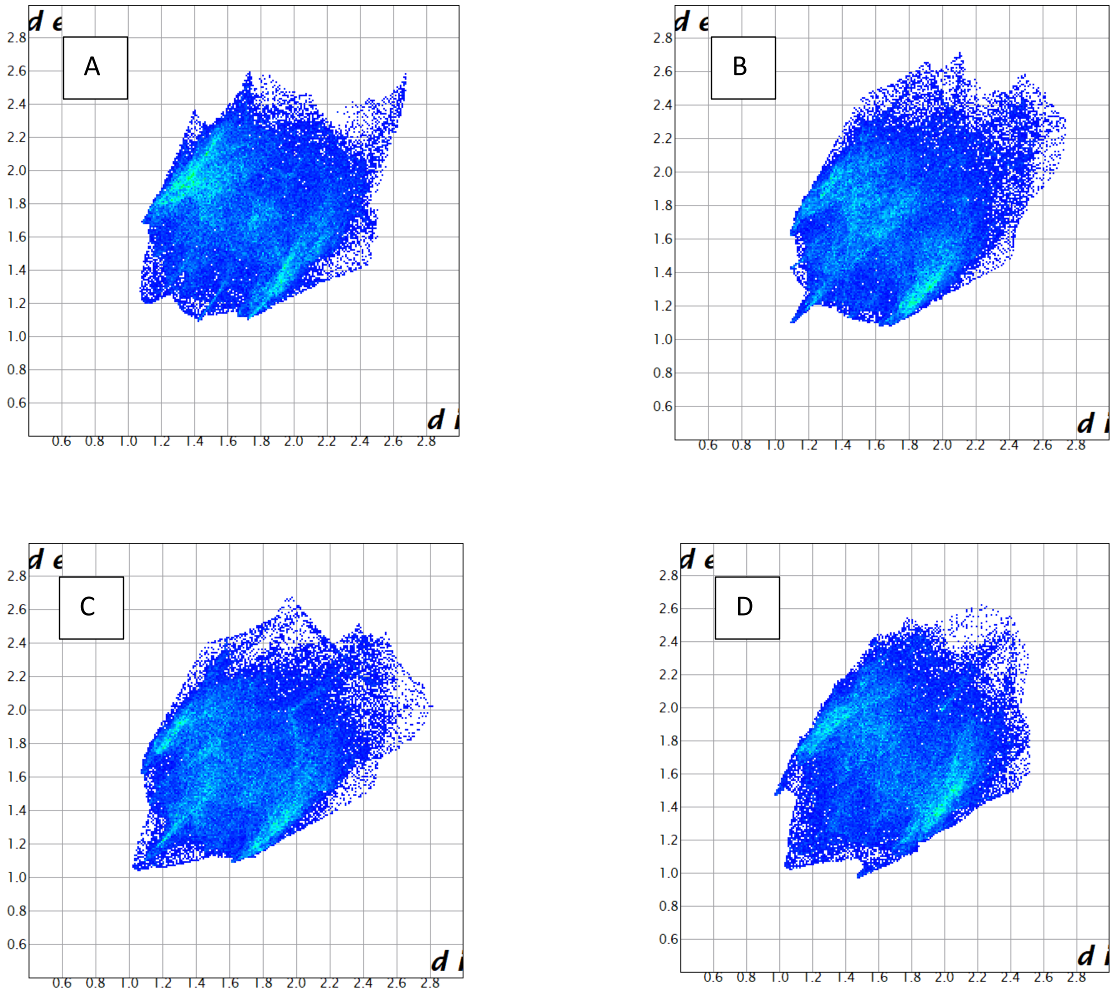

Figure S1. Fingerprint plots for the four distinct moieties in compound **2a**.

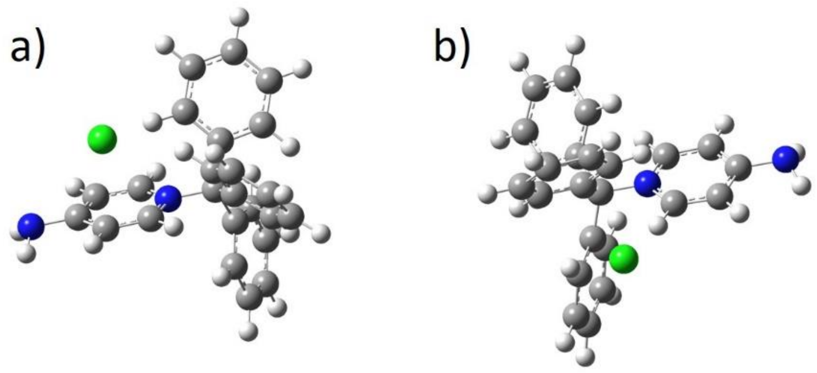

Figure S2. Starting structures used for optimization of compound **9** derivative of trityl-IL salts.

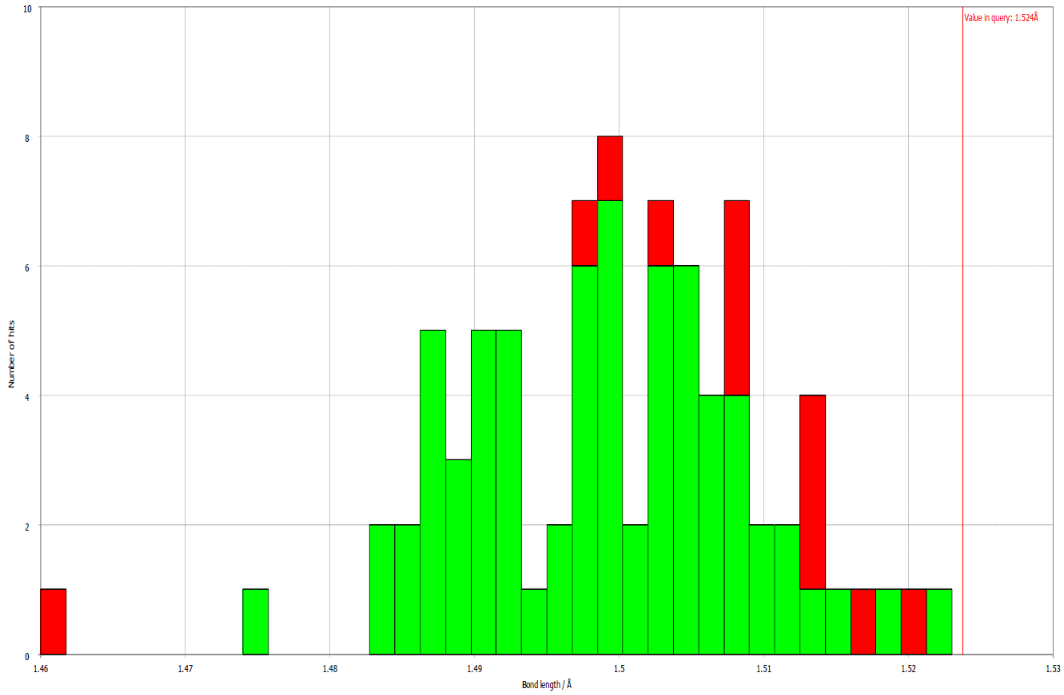

Figure S3. The results of the Mogul geometry search check for compound **9**.

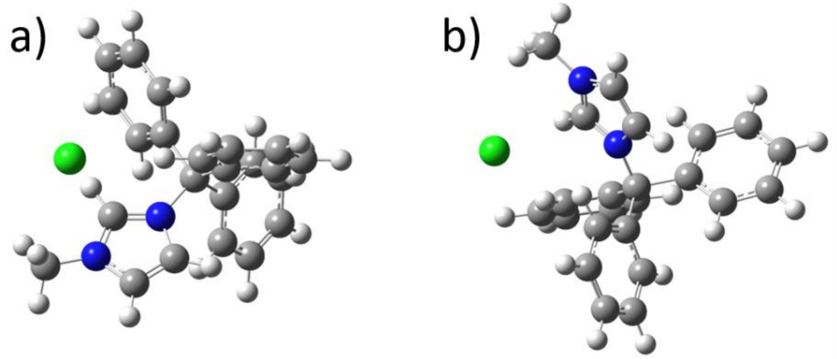

Figure S4. Starting structures used for optimization of compound **1** derivative of trityl-IL salts.

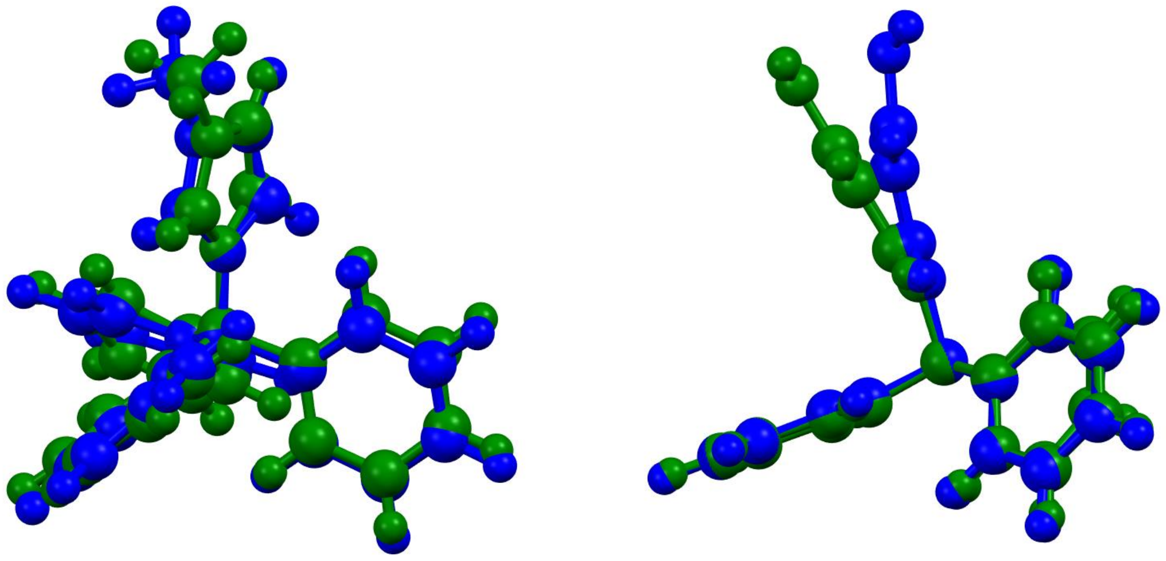

Figure S5. Overlaying images of crystal (green) and DFT optimized (blue) structures of compounds **1** (left) and **9** (right).

Table S2. Comparison of the bond length in compound **1** obtained from single crystal X-ray data with the simulated structure obtained using DFT method at M06-2X/6-31G++(d,p) theory and basis set.

Bond	Bond distance (Å)	
	X-ray data	simulated data
C24–N3	1.498	1.498
C25–N3	1.385	1.383
C27–N3	1.341	1.334
C27–N4	1.329	1.331
C28–N4	1.464	1.459
C26–N4	1.374	1.378
C26–C25	1.355	1.356
C29–C24	1.540	1.539
C29–C30	1.398	1.396
C30–C31	1.391	1.396
C31–C32	1.387	1.396
C32–C33	1.386	1.391
C34–C29	1.396	1.396

Table S3. Comparison of the bond length in compound **9** obtained from single crystal X-ray data with the simulated structure obtained using DFT method at M06-2X/6-31G++(d,p) theory and basis set.

**Bond**	**Bond distance (Å)**	
	**X-ray data**	**simulated data**
N1–C1	1.359	1.355
C1–C2	1.358	1.364
C2–C3	1.412	1.411
N2–C3	1.328	1.351
N1–C6	1.522	1.516
C3–C4	1.420	1.411
C4–C5	1.357	1.364
C6–C13	1.536	1.540
C10–C11	1.384	1.395
C13–C18	1.403	1.401
C14–C15	1.389	1.394
C15–C16	1.380	1.393
C16–C17	1.395	1.394
C17–C18	1.380	1.393

References

(1) Frisch, M. J.; Trucks, G. W.; Schlegel, H. B.; Scuseria, G. E.; Robb, M. A.; Cheeseman, J. R.; Scalmani, G.; Barone, V.; Petersson, G. A.; Nakatsuji, H.; Li, X.; Caricato, M.; Marenich, A. V.; Bloino, J.; Janesko, B. G.; Gomperts, R.; Mennucci, B.; Hratchian, H. P.; Ortiz, J. V.; Izmaylov, A. F.; Sonnenberg, J. L.; Williams-Young, D.; Ding, F.; Lipparini, F.; Egidi, F.; Goings, J.; Peng, B.; Petrone, A.; Henderson, T.; Ranasinghe, D.; Zakrzewski, V. G.; Gao, J.; Rega, N.; Zheng, G.; Liang, W.; Hada, M.; Ehara, M.; Toyota, K.; Fukuda, R.; Hasegawa, J.; Ishida, M.; Nakajima, T.; Honda, Y.; Kitao, O.; Nakai, H.; Vreven, T.; Throssell, K.; Montgomery, J. A., Jr.; Peralta, J. E.; Ogliaro, F.; Bearpark, M. J.; Heyd, J. J.; Brothers, E. N.; Kudin, K. N.; Staroverov, V. N.; Keith, T. A.; Kobayashi, R.; Normand, J.; Raghavachari, K.; Rendell, A.; Burant, J.; Iyengar, S.; Tomasi, J.; Cossi, M.; Millam, J. M.; Klene, M.; Adamo, C.; Cammi, R.; Ochterski, J. W.; Martin, R. L.; Morokuma, K.; Farkas, O.; Foresman, J. B.; Fox, D. J. *Gaussian 16 C.0.1*; Gaussian, Inc.: Wallingford CT, 2016.

(2) Dennington, R.; Kieth, T. A.; Millam, J. M. *GaussView*; Semichem Inc.: Shawnee Mission, KS, 2016.

(3) Zhao, Y.; Truhlar, D. G. Density Functionals with Broad Applicability in Chemistry. *Acc. Chem. Res.* **2008**, *41* (2), 157–167. https://doi.org/10.1021/ar700111a.

(4) Boys, S. F.; Bernardi, F. The Calculation of Small Molecular Interactions by the Differences of Separate Total Energies. Some Procedures with Reduced Errors. *null* **1970**, *19* (4), 553–566. https://doi.org/10.1080/00268977000101561.

(5) Krause, L.; Herbst-Irmer, R.; Sheldrick, G. M.; Stalke, D. Comparison of Silver and Molybdenum Microfocus X-Ray Sources for Single-Crystal Structure Determination. *J Appl Cryst* **2015**, *48* (1), 3–10. https://doi.org/10.1107/S1600576714022985.

(6) Bruker. *Apex3 V2019.1-0, SAINT V8.40A*; Bruker Advanced X-ray Solutions; Bruker AXS Inc: Madison, Wisconsin, USA, 2019.

(7) *SHELXTL Suite of Programs*; Bruker Advanced X-ray Solutions; Bruker AXS Inc: Madison, Wisconsin, USA, 2000.

(8) Sheldrick, G. M. A Short History of SHELX. *Acta Cryst A* **2008**, *64* (1), 112–122. https://doi.org/10.1107/S0108767307043930.

(9) Sheldrick, G. M. Crystal Structure Refinement with SHELXL. *Acta Cryst C* **2015**, *71* (1), 3–8. https://doi.org/10.1107/S2053229614024218.

(10) Hübschle, C. B.; Sheldrick, G. M.; Dittrich, B. ShelXle: A Qt Graphical User Interface for SHELXL. *J Appl Cryst* **2011**, *44* (6), 1281–1284. https://doi.org/10.1107/S0021889811043202.

(11) Spackman, P. R.; Turner, M. J.; McKinnon, J. J.; Wolff, S. K.; Grimwood, D. J.; Jayatilaka, D.; Spackman, M. A. *CrystalExplorer* : A Program for Hirshfeld Surface Analysis, Visualization and Quantitative Analysis of Molecular Crystals. *J Appl Crystallogr* **2021**, *54* (3), 1006–1011. https://doi.org/10.1107/S1600576721002910.

(12) Dolomanov, O. V.; Bourhis, L. J.; Gildea, R. J.; Howard, J. A. K.; Puschmann, H. *OLEX2* : A Complete Structure Solution, Refinement and Analysis Program. *Journal of Applied Crystallography* **2009**, *42* (2), 339–341. https://doi.org/10.1107/S0021889808042726.

(13) Macrae, C. F.; Bruno, I. J.; Chisholm, J. A.; Edgington, P. R.; McCabe, P.; Pidcock, E.; Rodriguez-Monge, L.; Taylor, R.; van de Streek, J.; Wood, P. A. *Mercury CSD 2.0* – New Features for the Visualization and Investigation of Crystal Structures. *Journal of Applied Crystallography* **2008**, *41* (2), 466–470. https://doi.org/10.1107/S0021889807067908.

(14) Lu, T.; Chen, F. Multiwfn: A Multifunctional Wavefunction Analyzer. *J. Comput. Chem.* **2012**, *33* (5), 580–592. https://doi.org/10.1002/jcc.22885.

(15) Humphrey, W.; Dalke, A.; Schulten, K. VMD: Visual Molecular Dynamics. *Journal of Molecular Graphics* **1996**, *14* (1), 33–38. https://doi.org/10.1016/0263-7855(96)00018-5.

(16) Neese, F. Software Update: The ORCA Program System, Version 4.0. *WIREs Computational Molecular Science* **2018**, *8* (1), e1327. https://doi.org/10.1002/wcms.1327.

(17) Mardirossian, N.; Head-Gordon, M. ΩB97X-V: A 10-Parameter, Range-Separated Hybrid, Generalized Gradient Approximation Density Functional with Nonlocal Correlation, Designed by a Survival-of-the-Fittest Strategy. *Physical Chemistry Chemical Physics* **2014**, *16* (21), 9904. https://doi.org/10.1039/c3cp54374a.

(18) Kleemiss, F.; Dolomanov, O. V.; Bodensteiner, M.; Peyerimhoff, N.; Midgley, L.; Bourhis, L. J.; Genoni, A.; Malaspina, L. A.; Jayatilaka, D.; Spencer, J. L.; White, F.; Grundkötter-Stock, B.; Steinhauer, S.; Lentz, D.; Puschmann, H.; Grabowsky, S. Accurate Crystal Structures and Chemical Properties from NoSpherA2. *Chem. Sci.* **2021**, *12* (5), 1675–1692. https://doi.org/10.1039/D0SC05526C.

^{1}H and ^{13}C NMR spectra of products

1

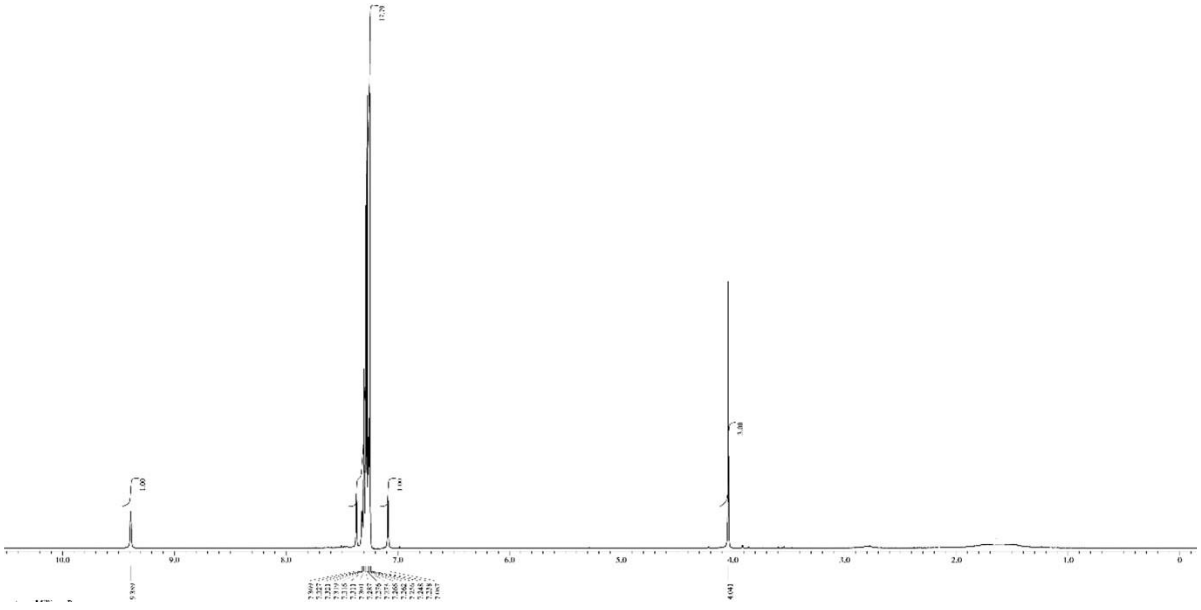

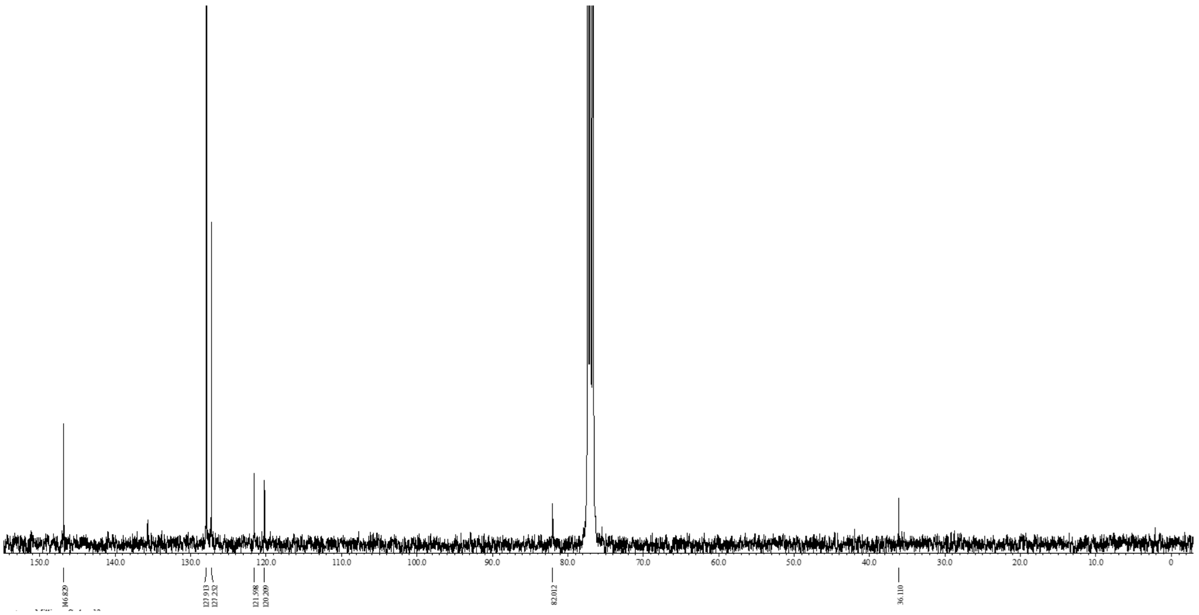

2

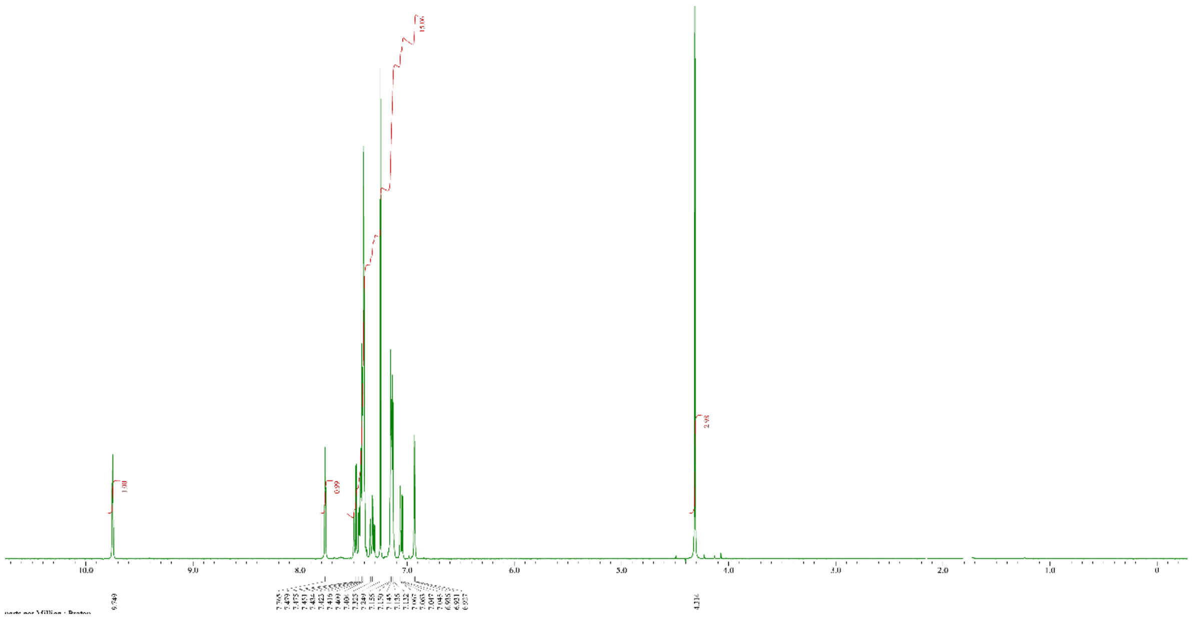

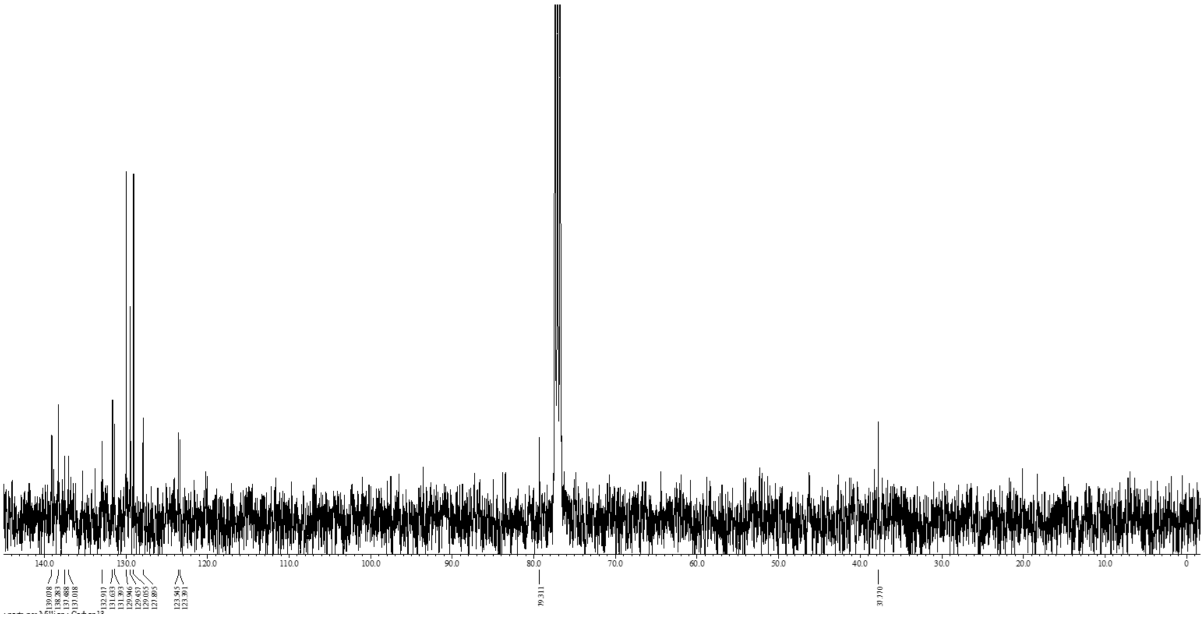

3

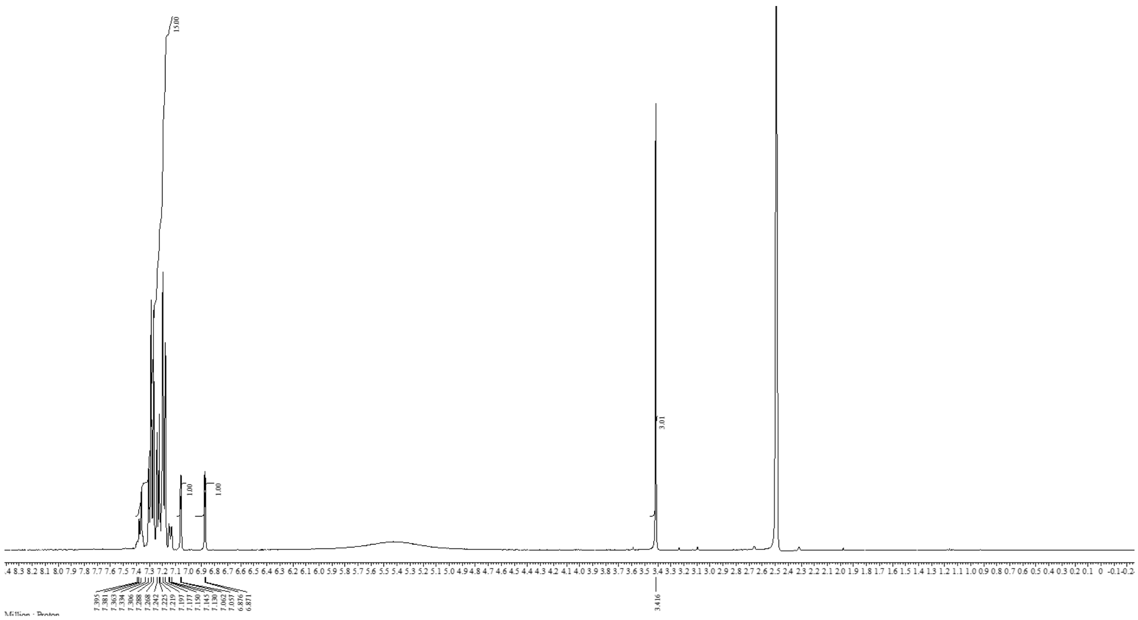

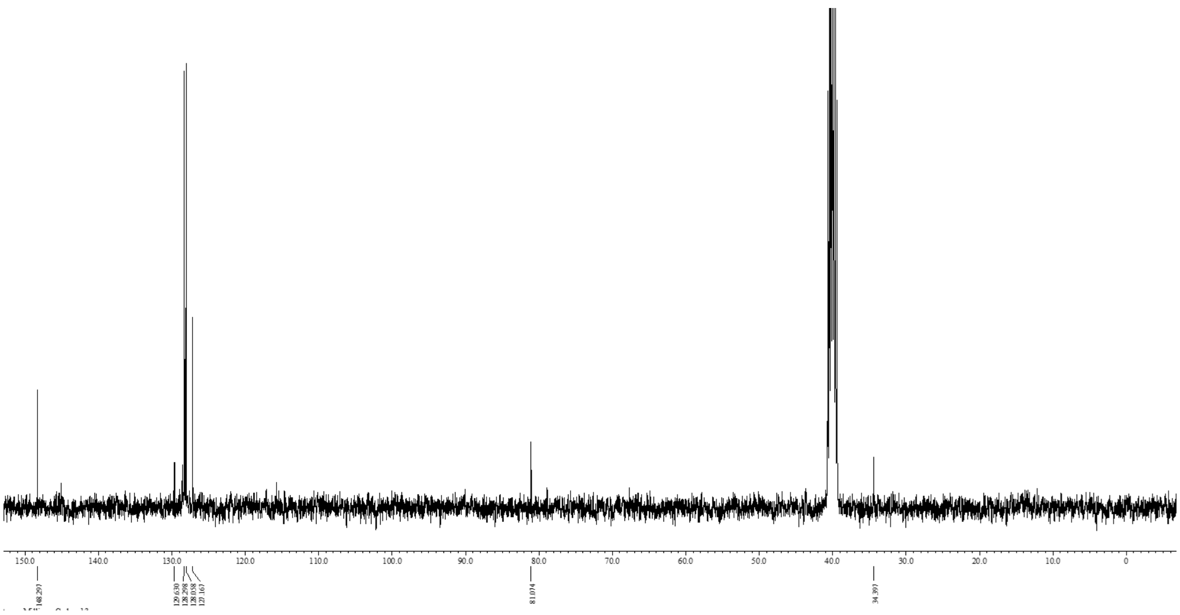

4

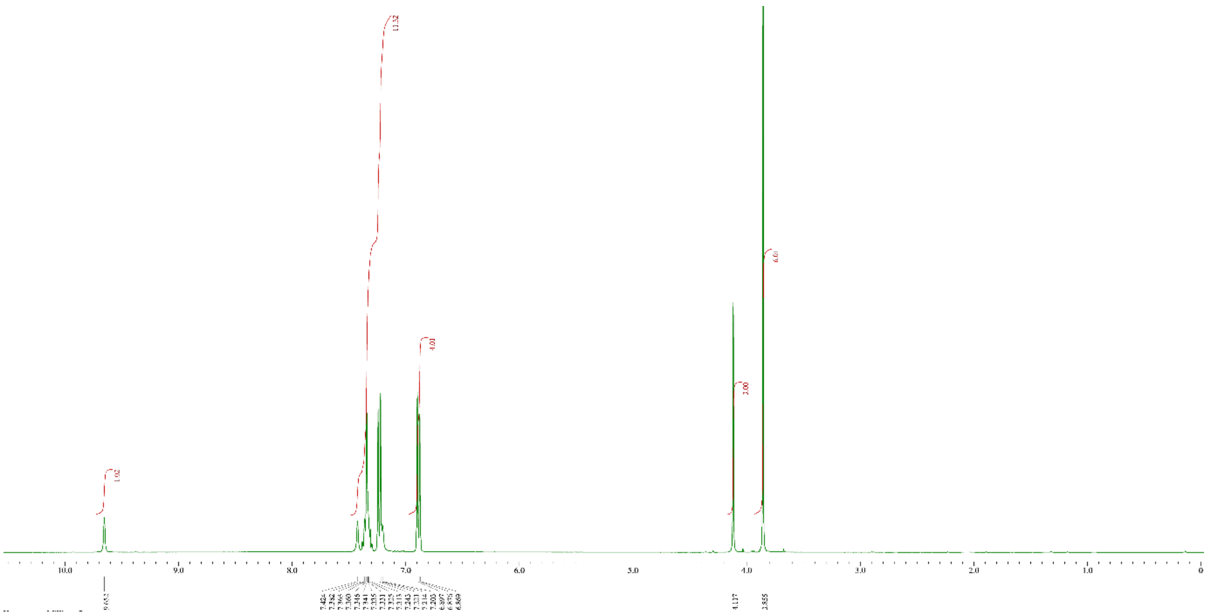

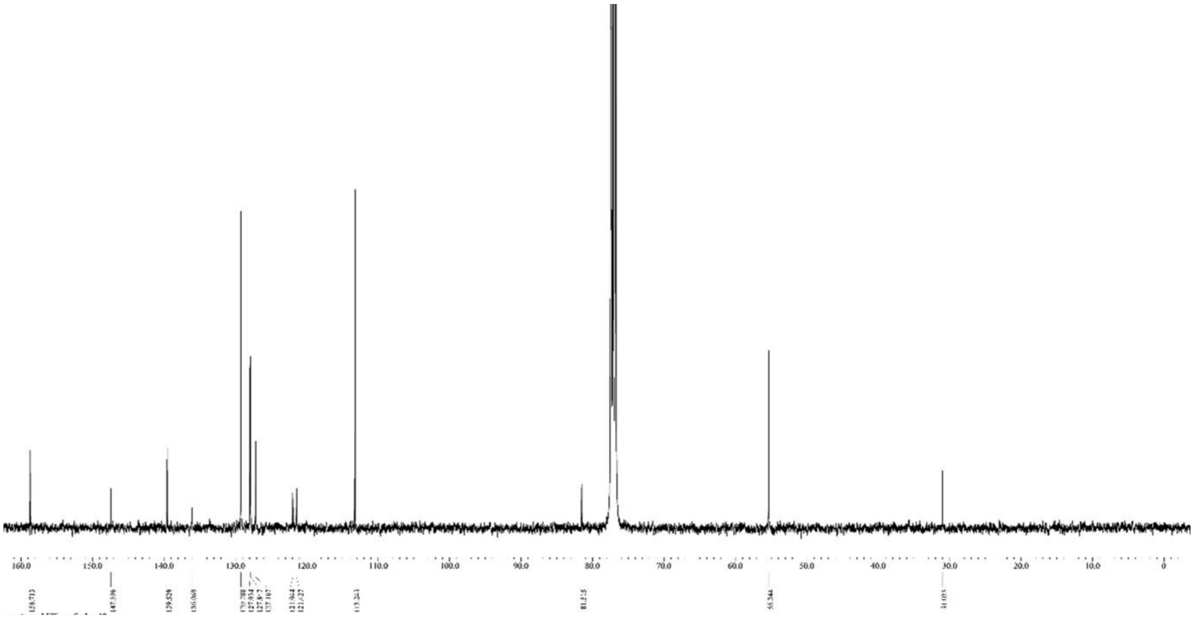

5

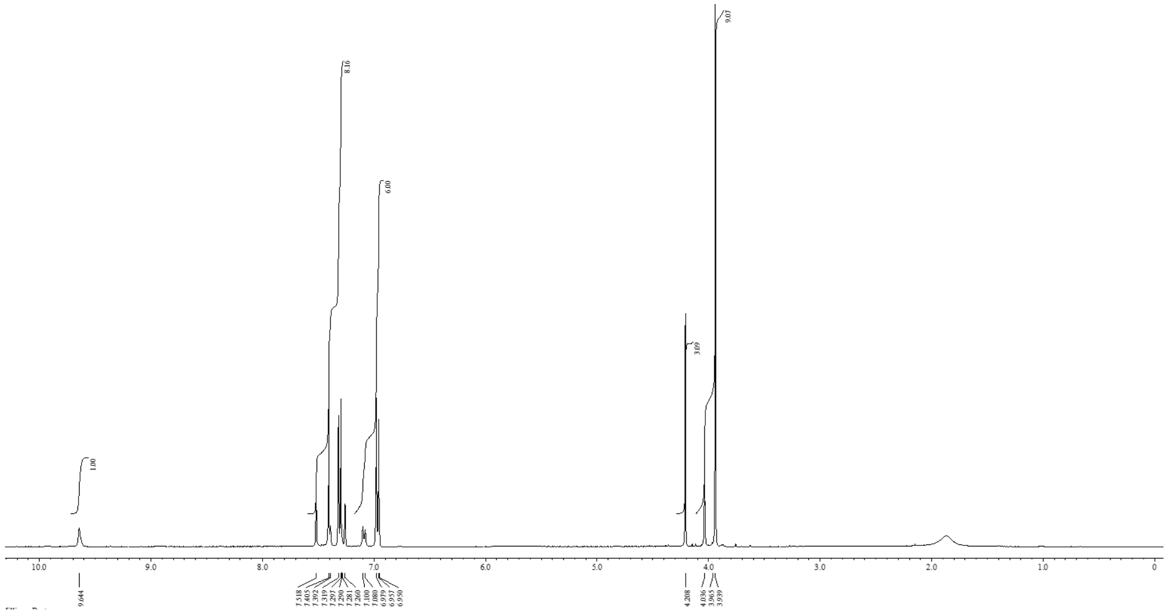

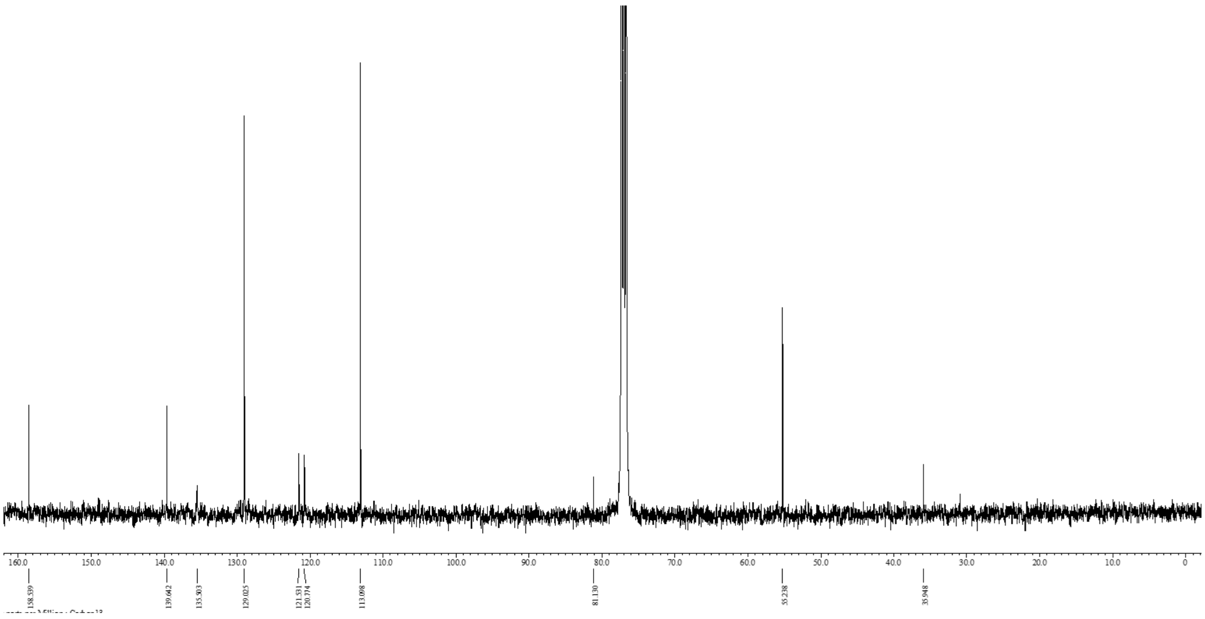

6

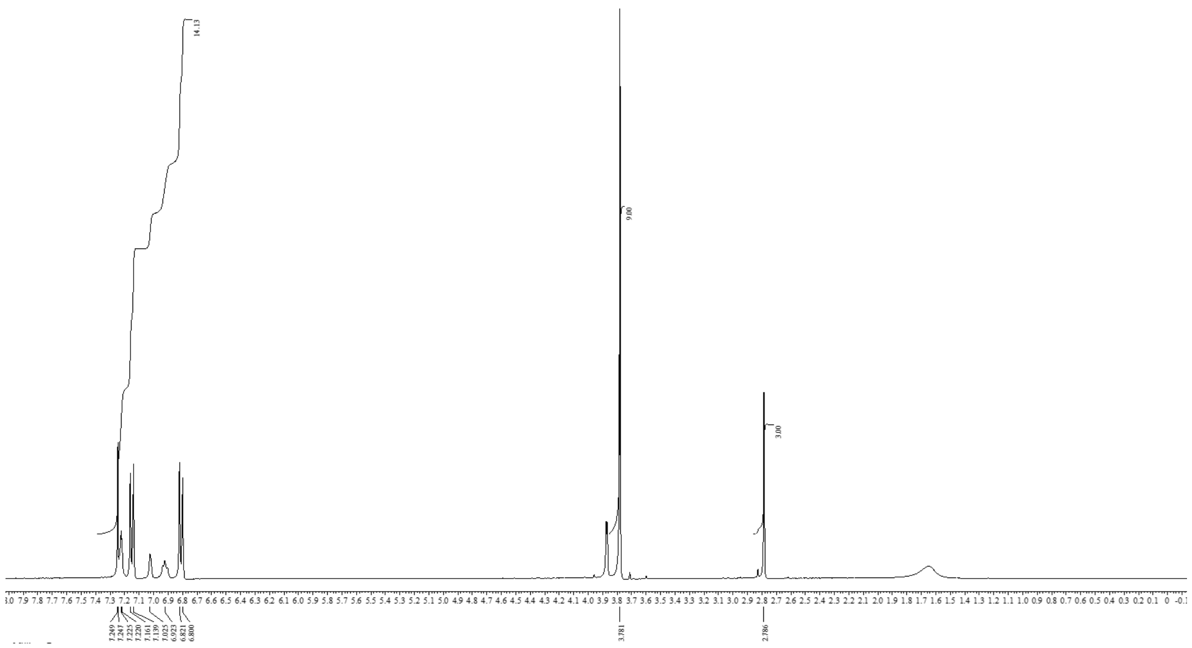

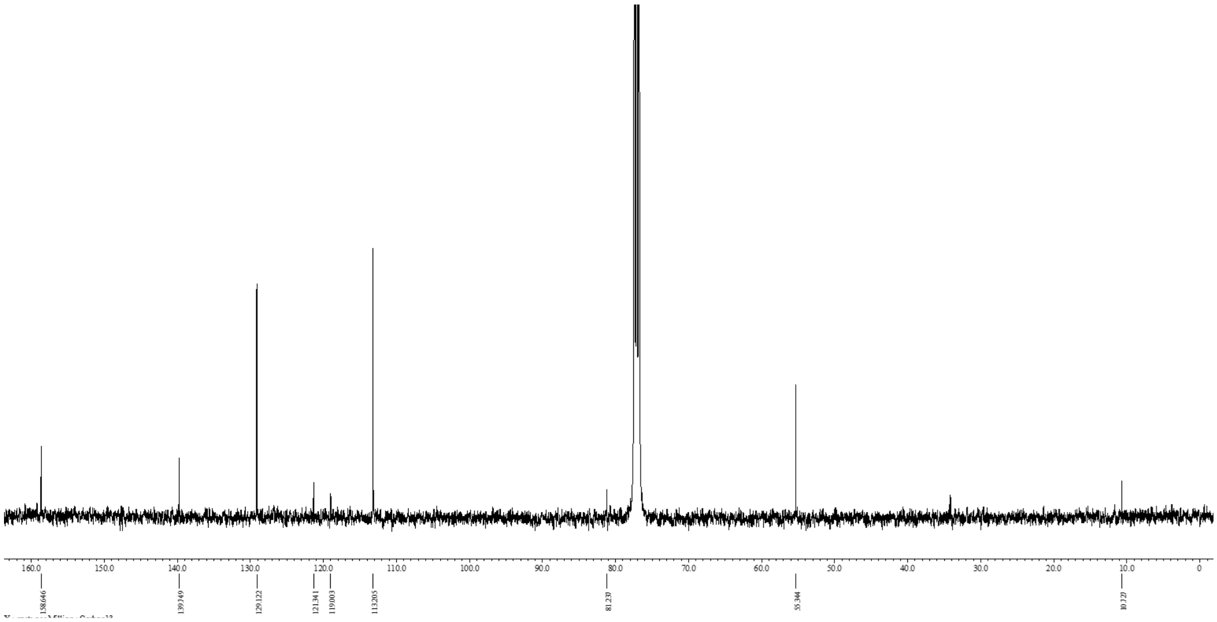

7

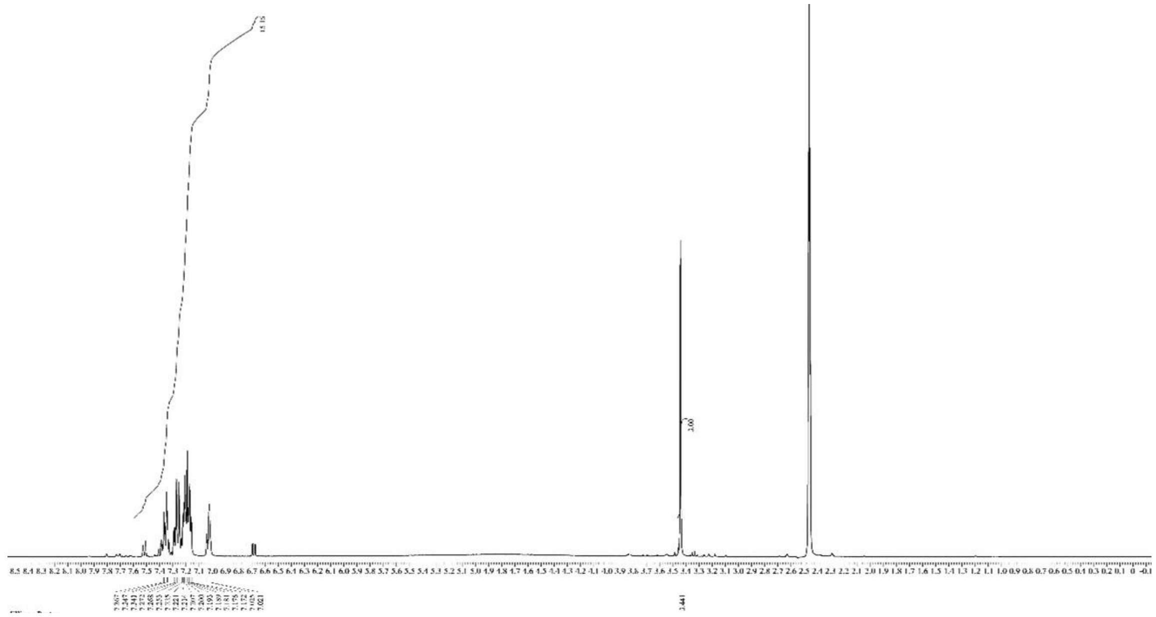

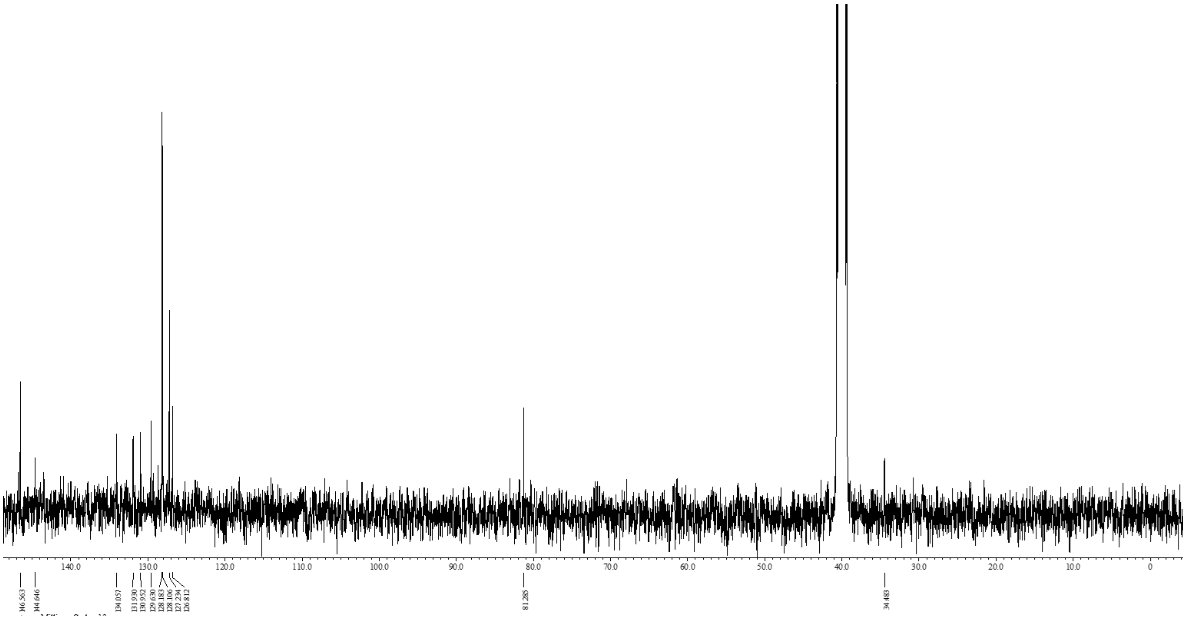

8

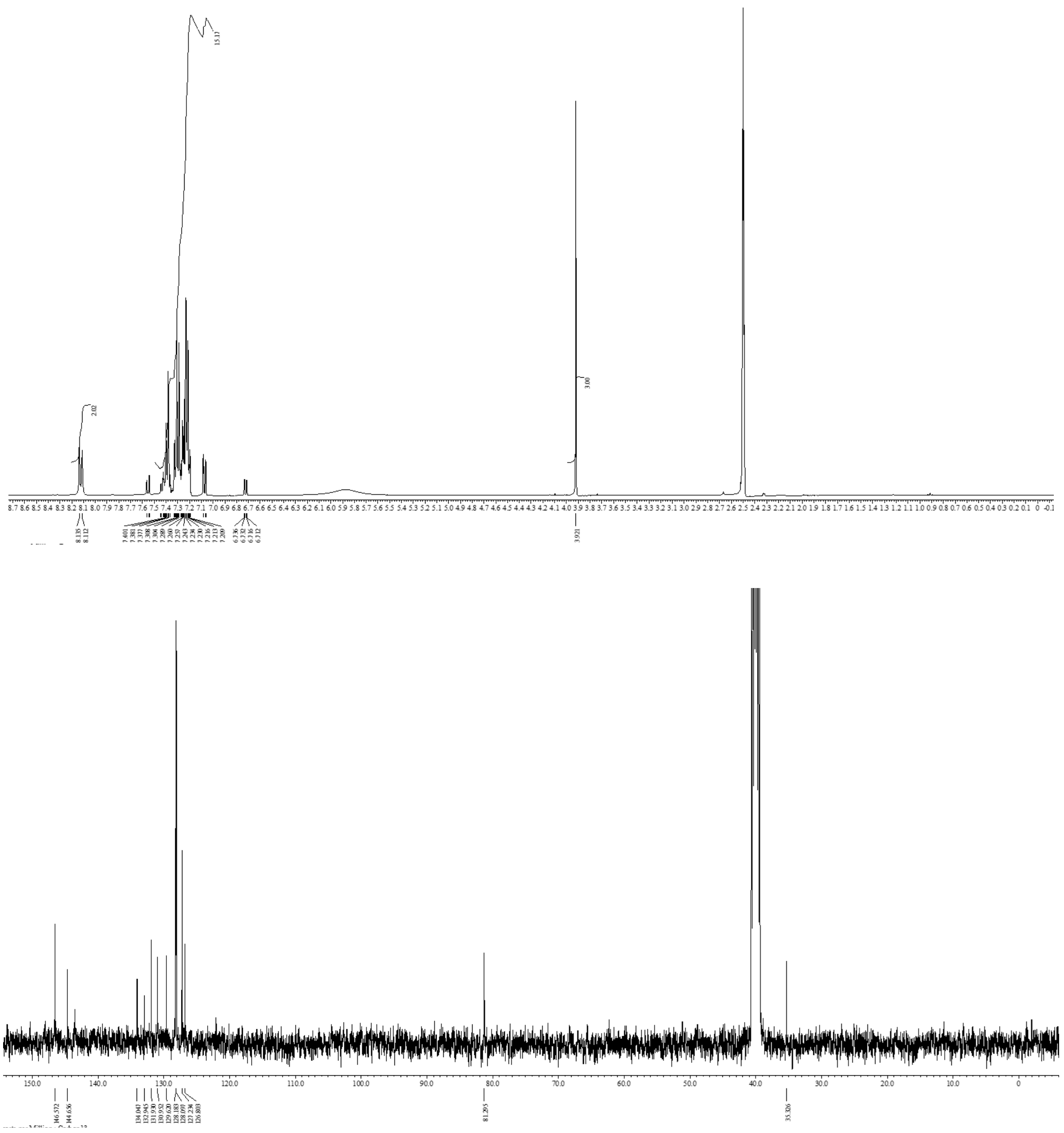

9

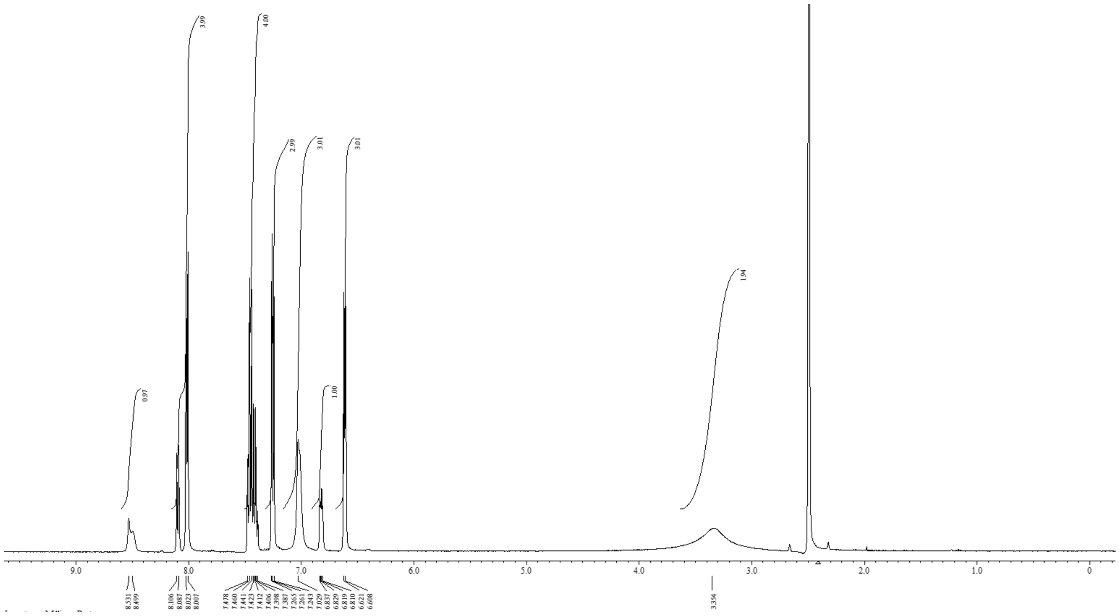
9.0
8.0
7.0
6.0
5.0
4.0
3.0
2.0
1.0
0
0.97
3.99
4.00
2.99
3.01
1.00
3.01
1.94

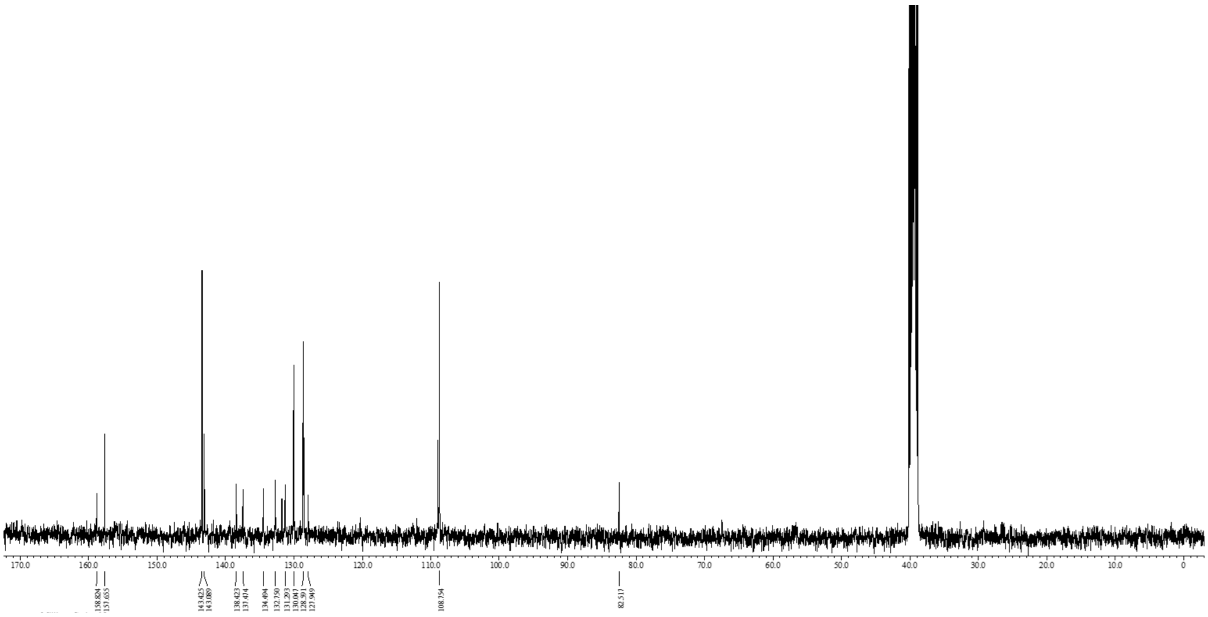
170.0
160.0
150.0
140.0
130.0
120.0
110.0
100.0
90.0
80.0
70.0
60.0
50.0
40.0
30.0
20.0
10.0
0
108.354
82.511

10

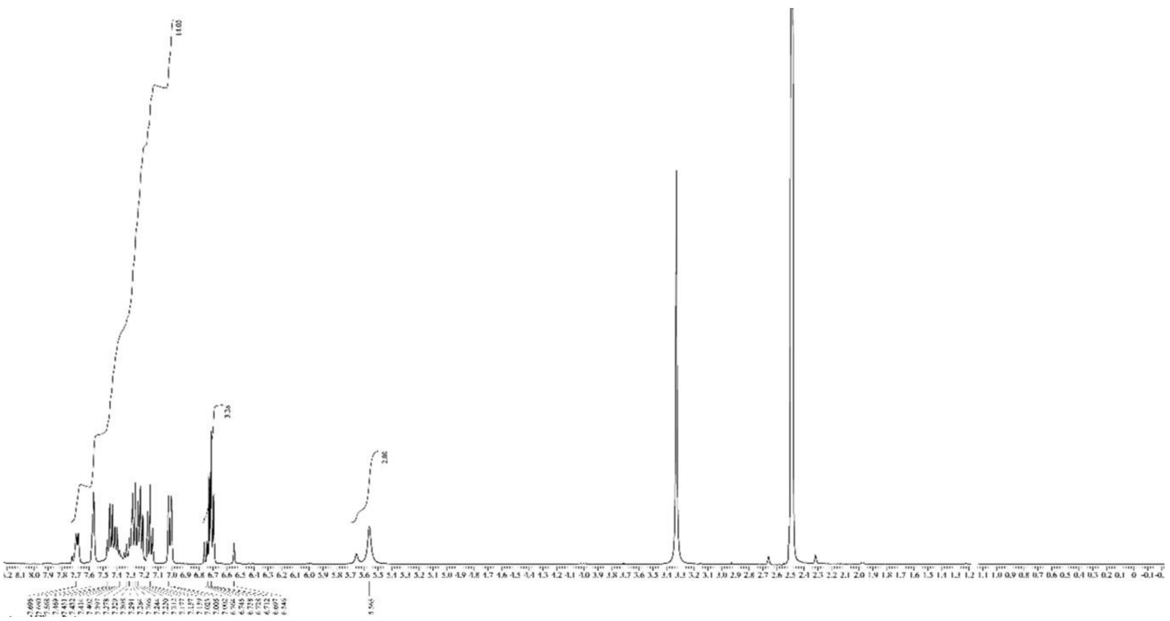

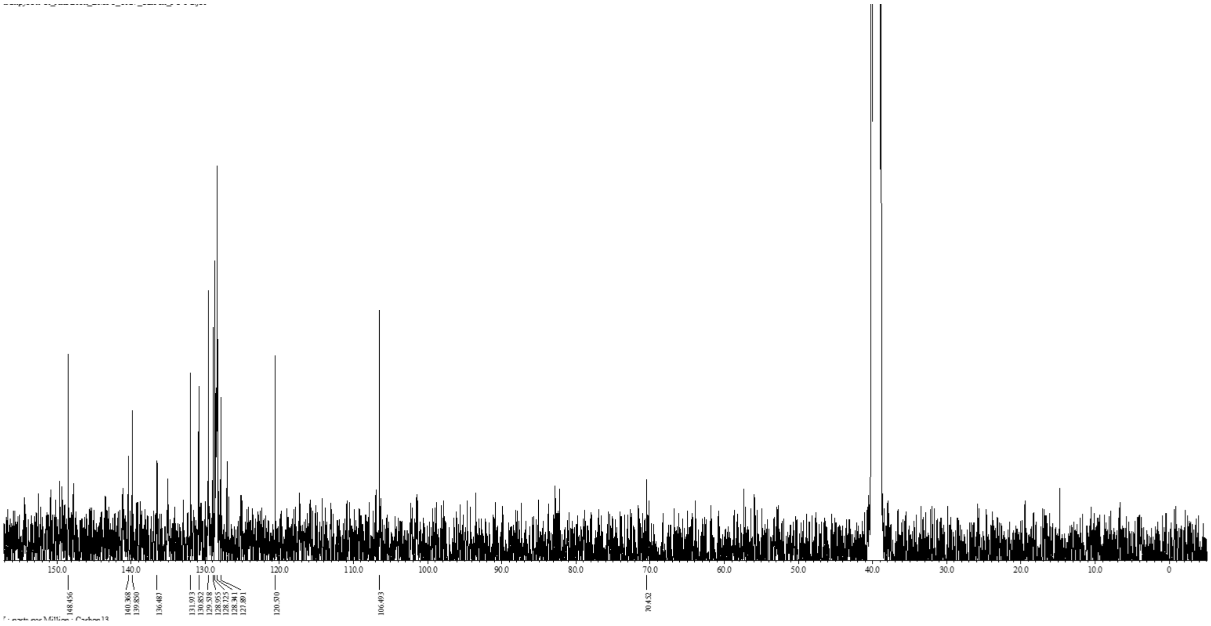

11

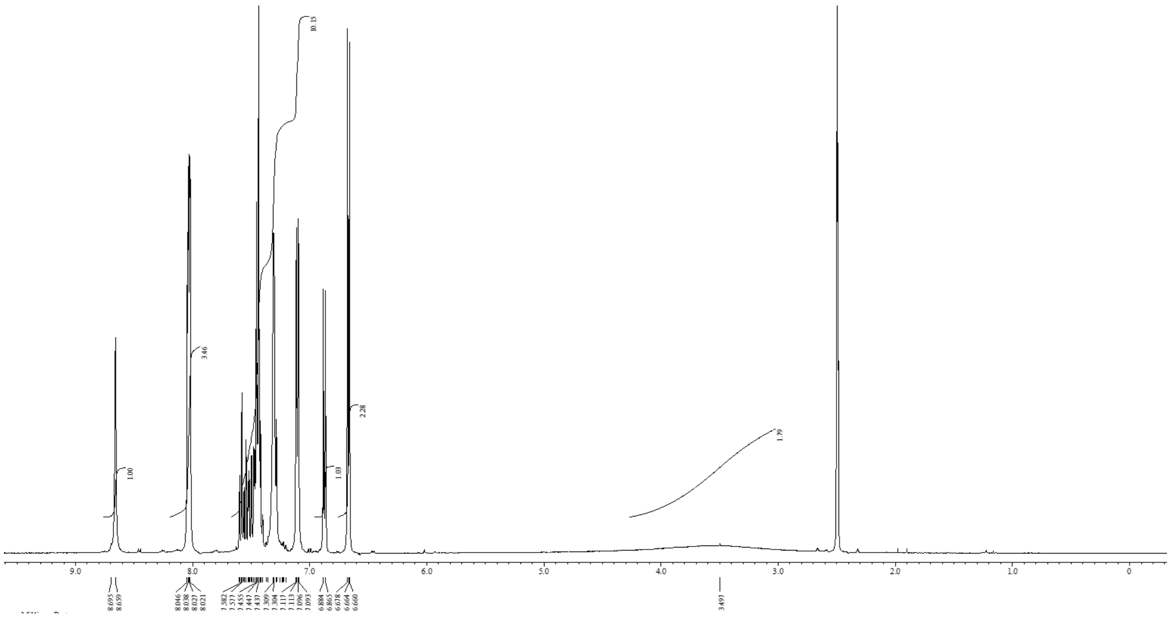

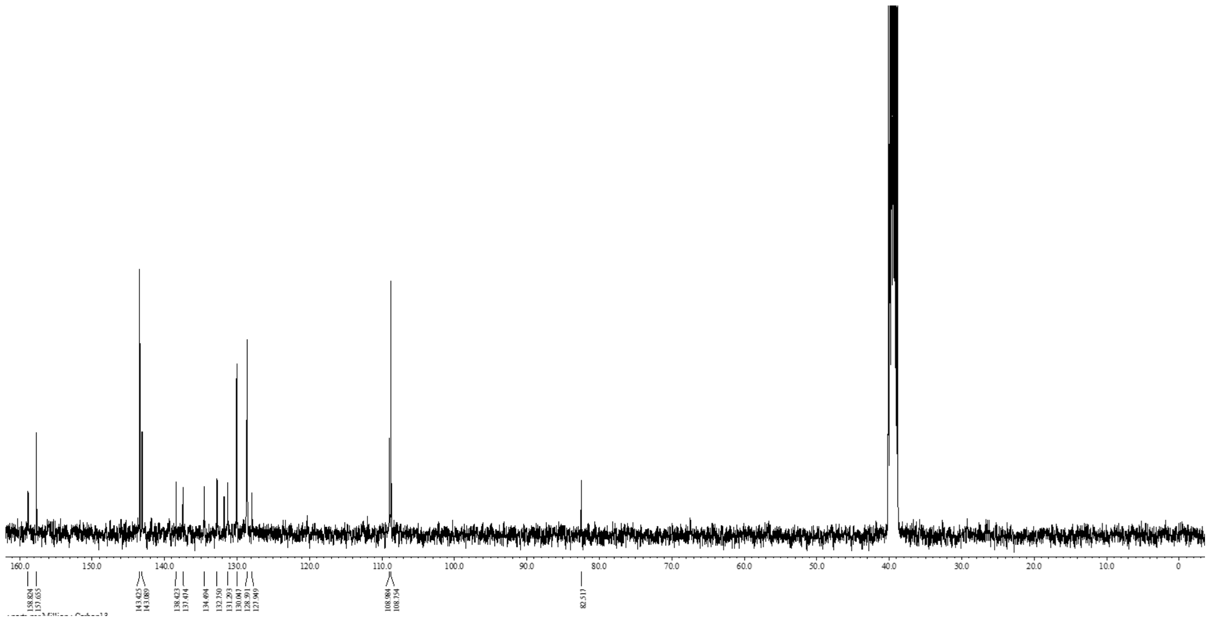

12

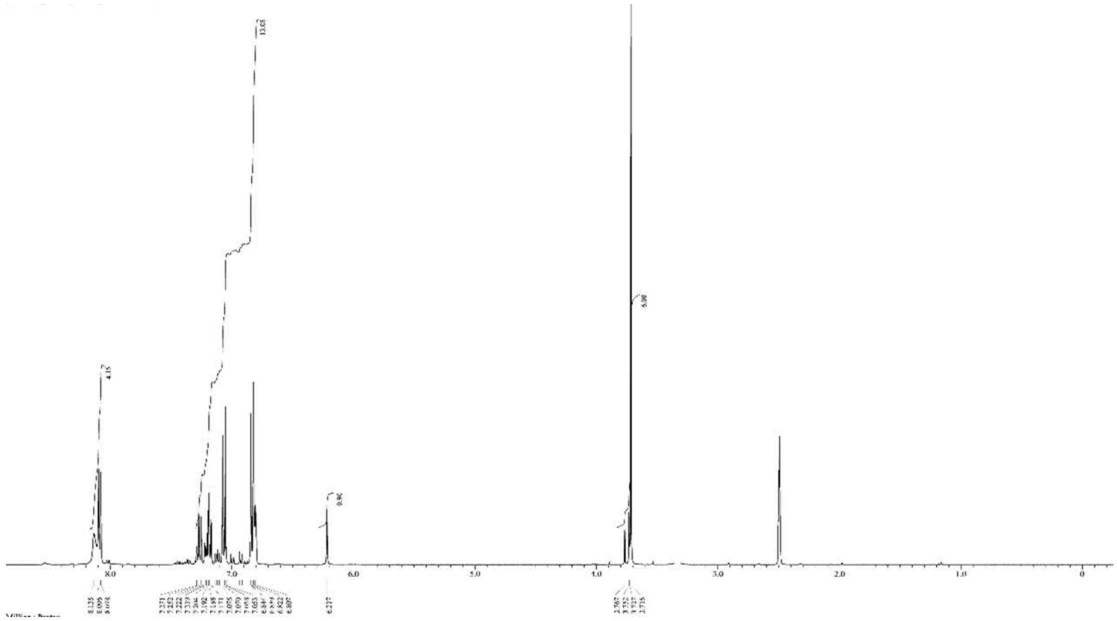

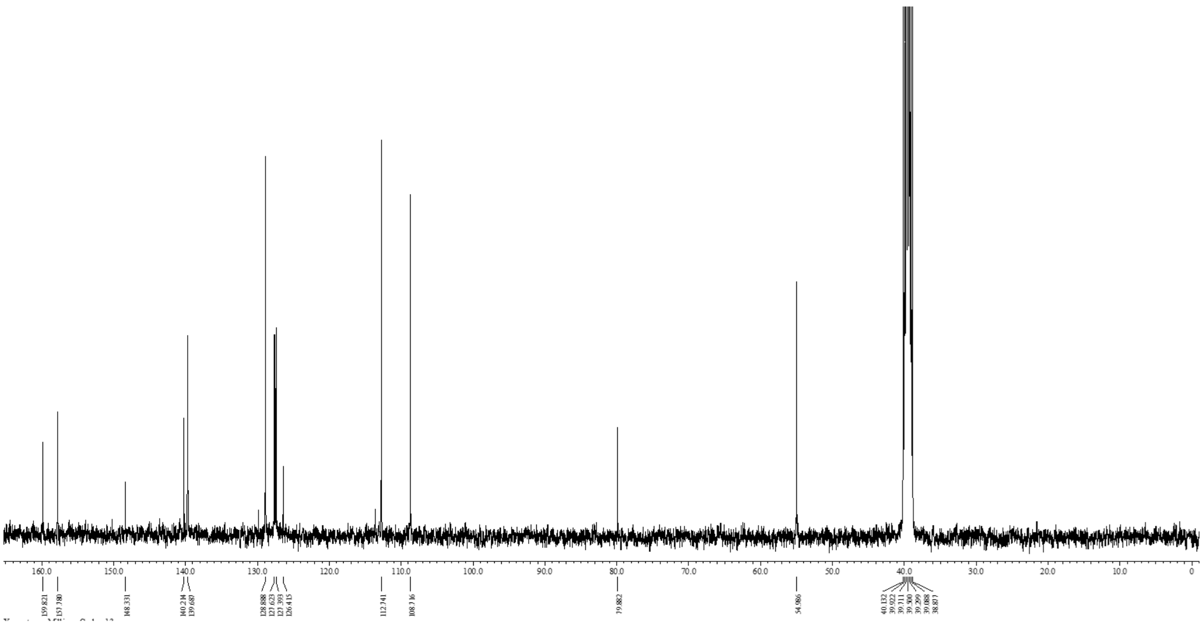

DSC and TGA thermograms of products

1

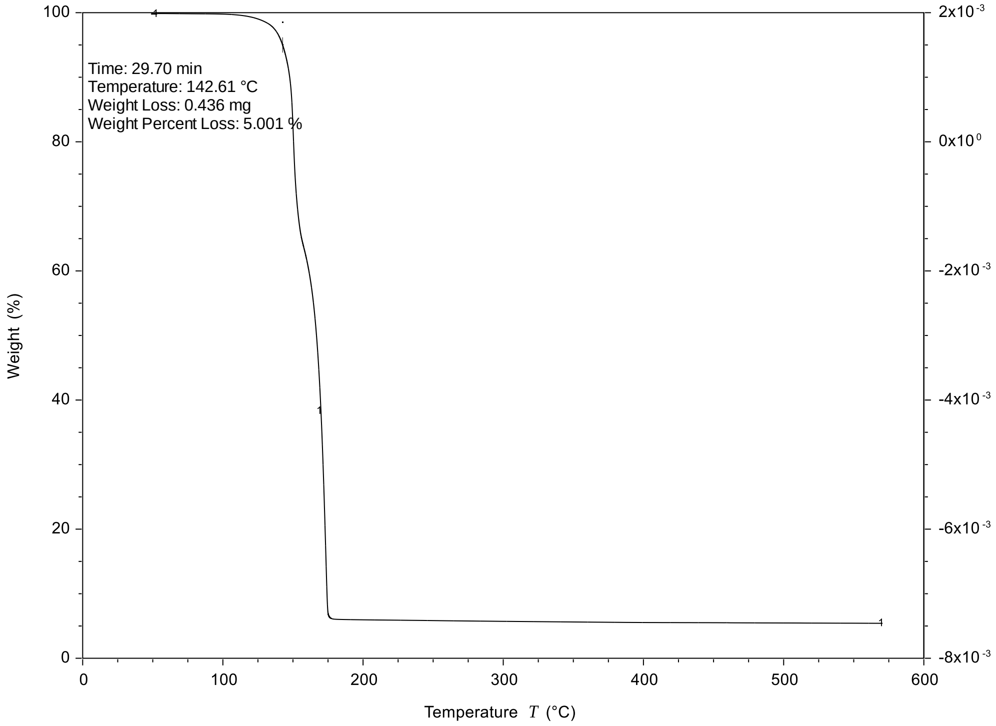

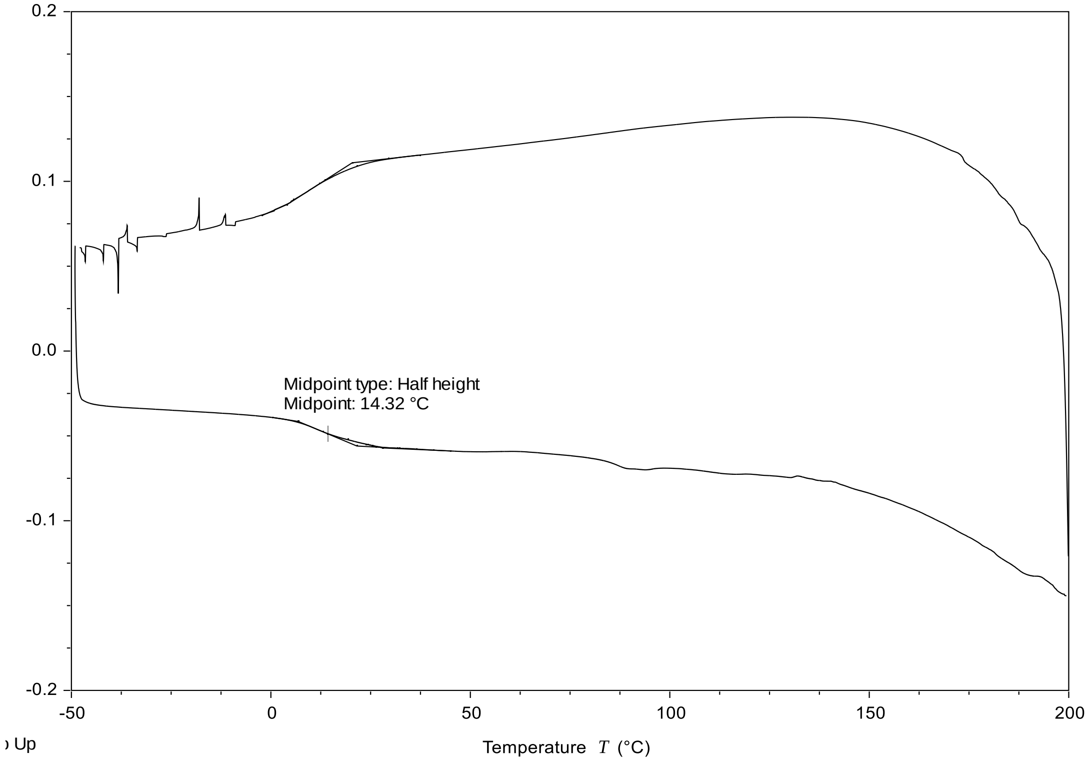

2

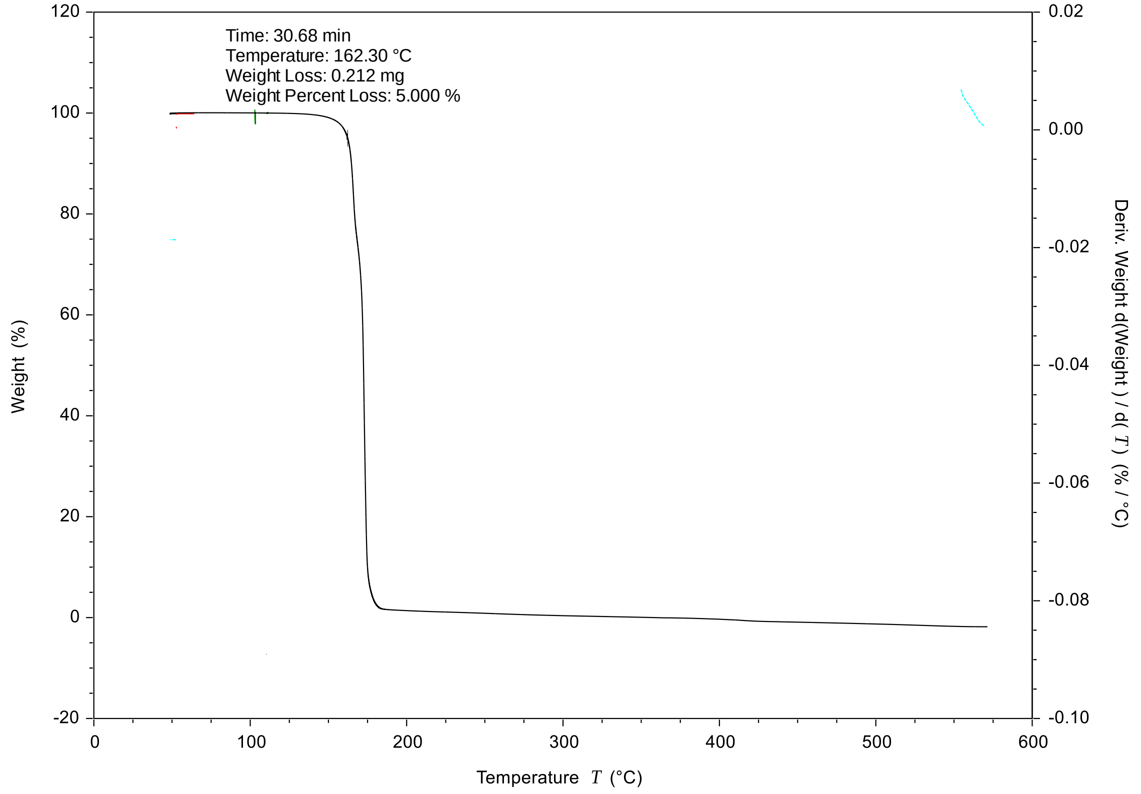
Time: 30.68 min
Temperature: 162.30 °C
Weight Loss: 0.212 mg
Weight Percent Loss: 5.000 %
Weight (%)
Deriv. Weight d(Weight) / d(T) (% / °C)
Temperature T (°C)

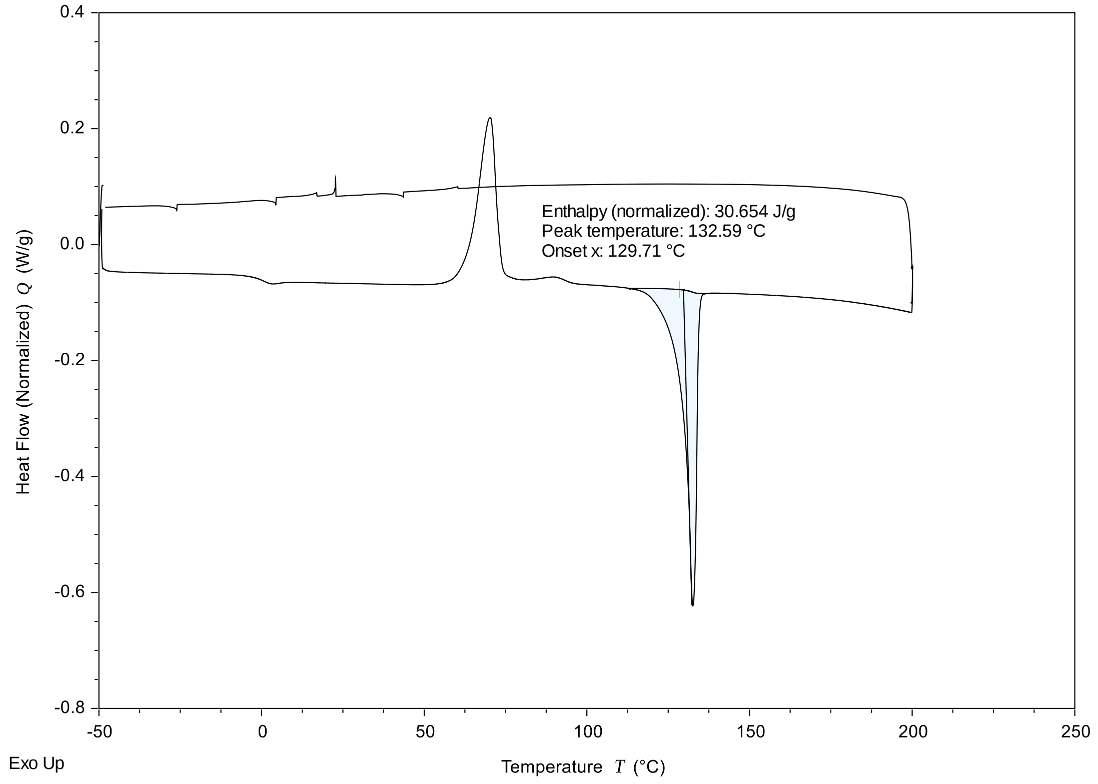
Enthalpy (normalized): 30.654 J/g
Peak temperature: 132.59 °C
Onset x: 129.71 °C
Heat Flow (Normalized) Q (W/g)
Temperature T (°C)
Exo Up

3

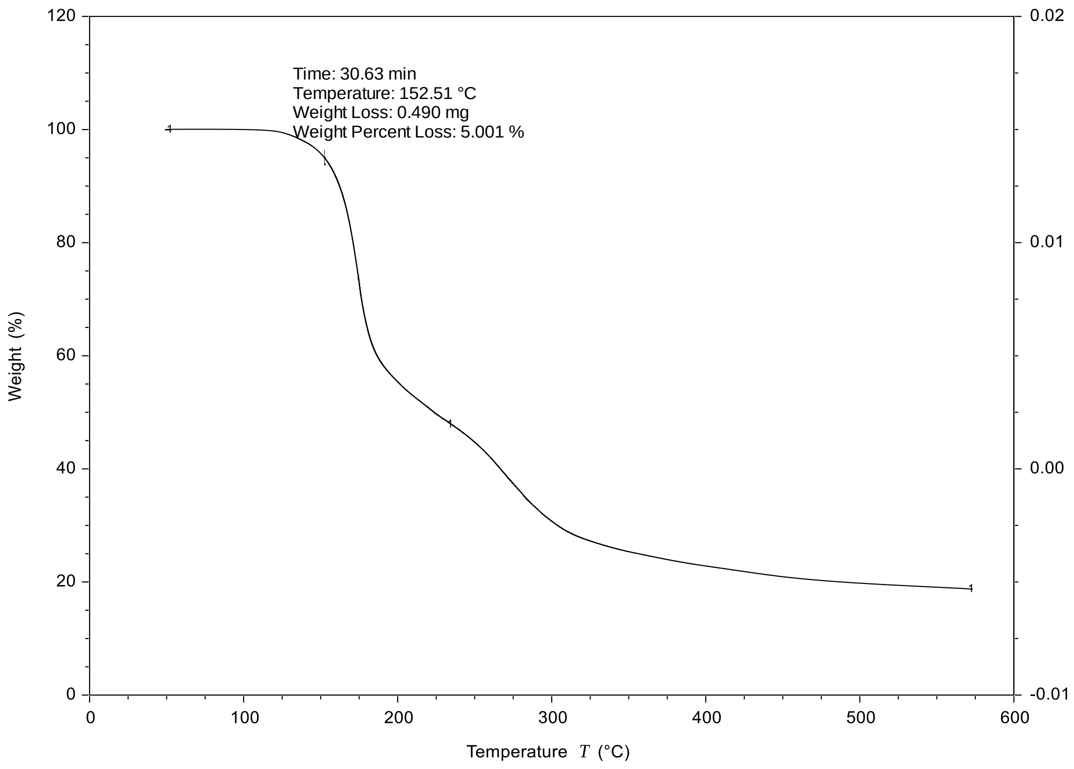
Time: 30.63 min
Temperature: 152.51 °C
Weight Loss: 0.490 mg
Weight Percent Loss: 5.001 %
Weight (%)
Temperature T (°C)

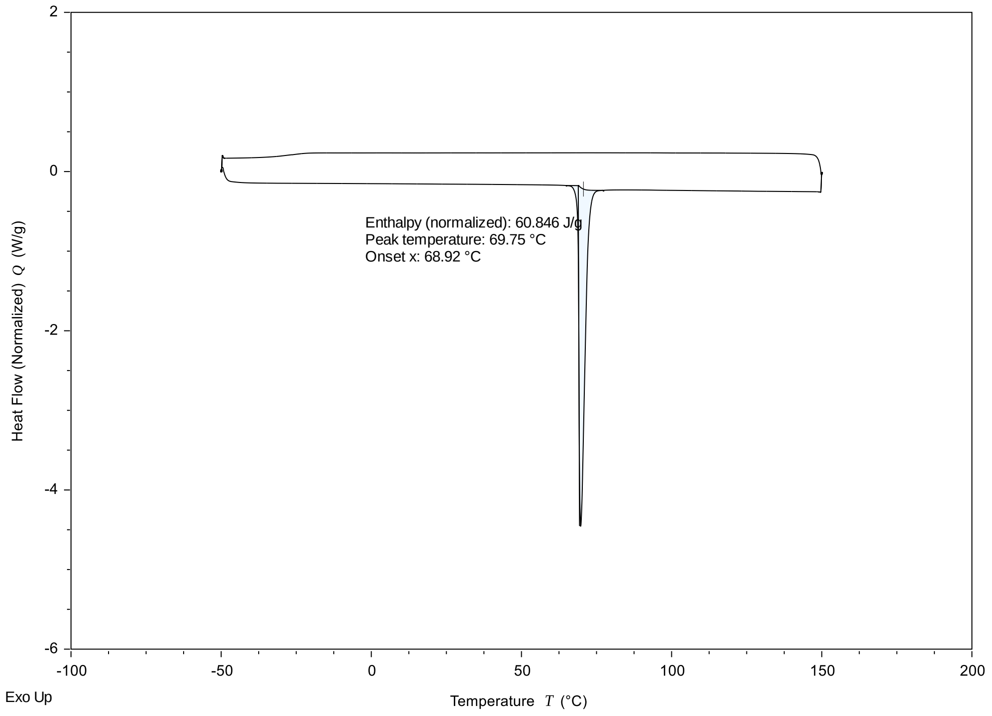
Enthalpy (normalized): 60.846 J/g
Peak temperature: 69.75 °C
Onset x: 68.92 °C
Heat Flow (Normalized) Q (W/g)
Temperature T (°C)
Exo Up

4

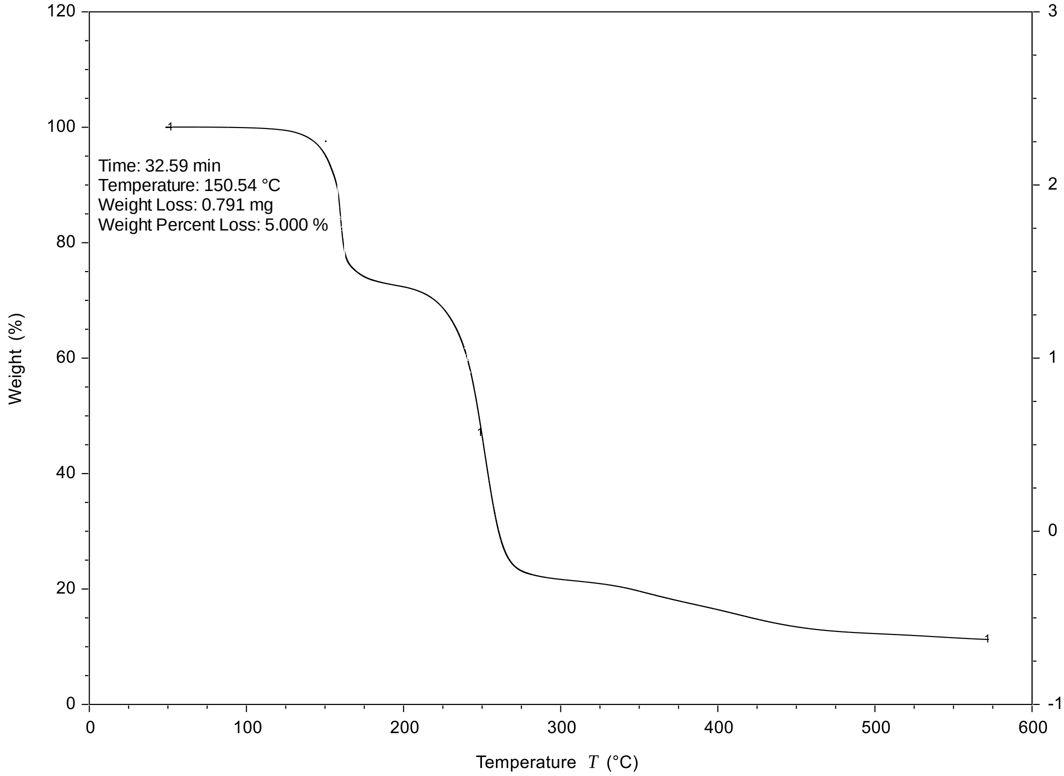
120
100
80
60
40
20
0
3
2
1
0
-1
0
100
200
300
400
500
600
Weight (%)
Temperature T (°C)
Time: 32.59 min
Temperature: 150.54 °C
Weight Loss: 0.791 mg
Weight Percent Loss: 5.000 %

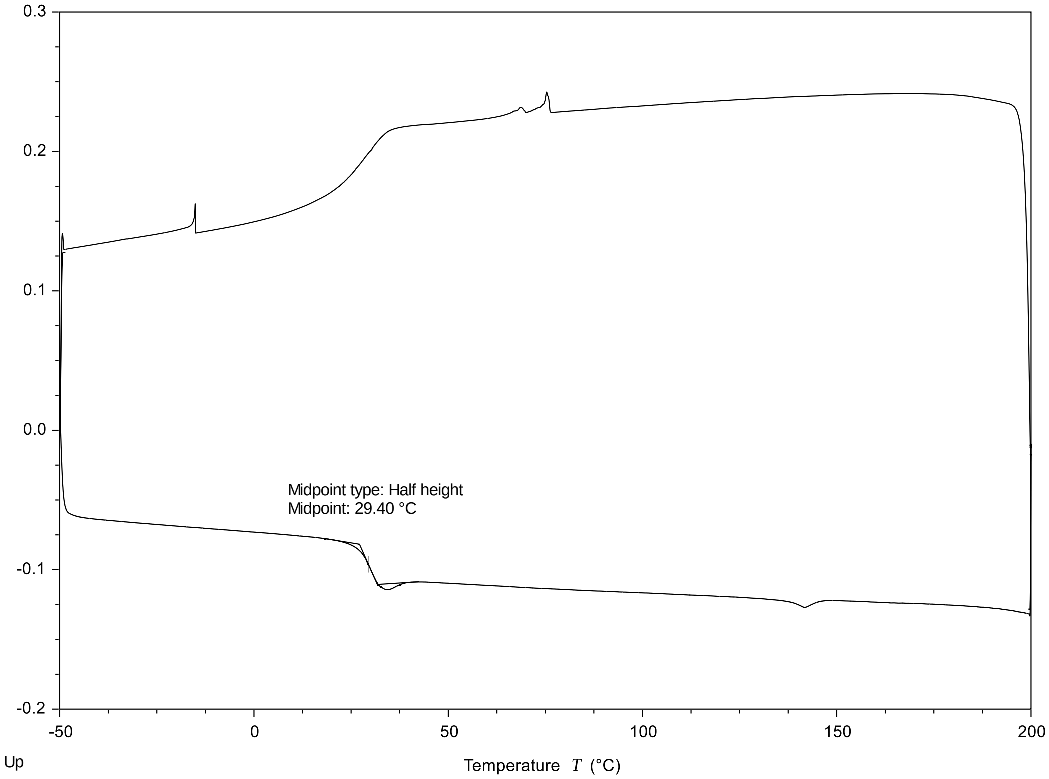
0.3
0.2
0.1
0.0
-0.1
-0.2
-50
0
50
100
150
200
Up
Temperature T (°C)
Midpoint type: Half height
Midpoint: 29.40 °C

5

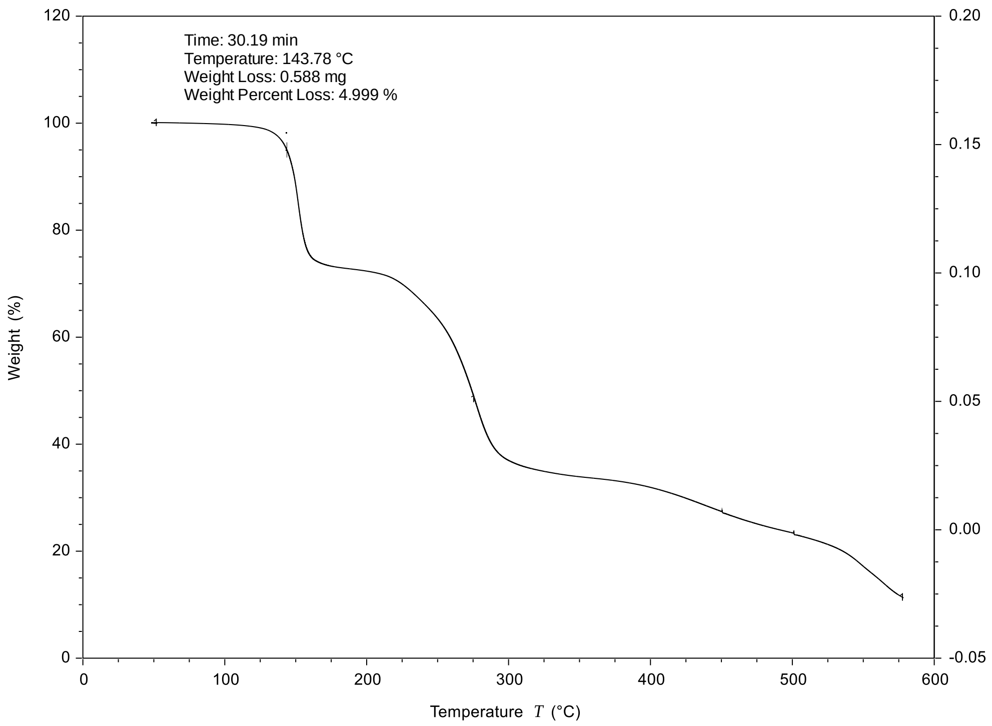
Time: 30.19 min
Temperature: 143.78 °C
Weight Loss: 0.588 mg
Weight Percent Loss: 4.999 %
Weight (%)
Temperature T (°C)

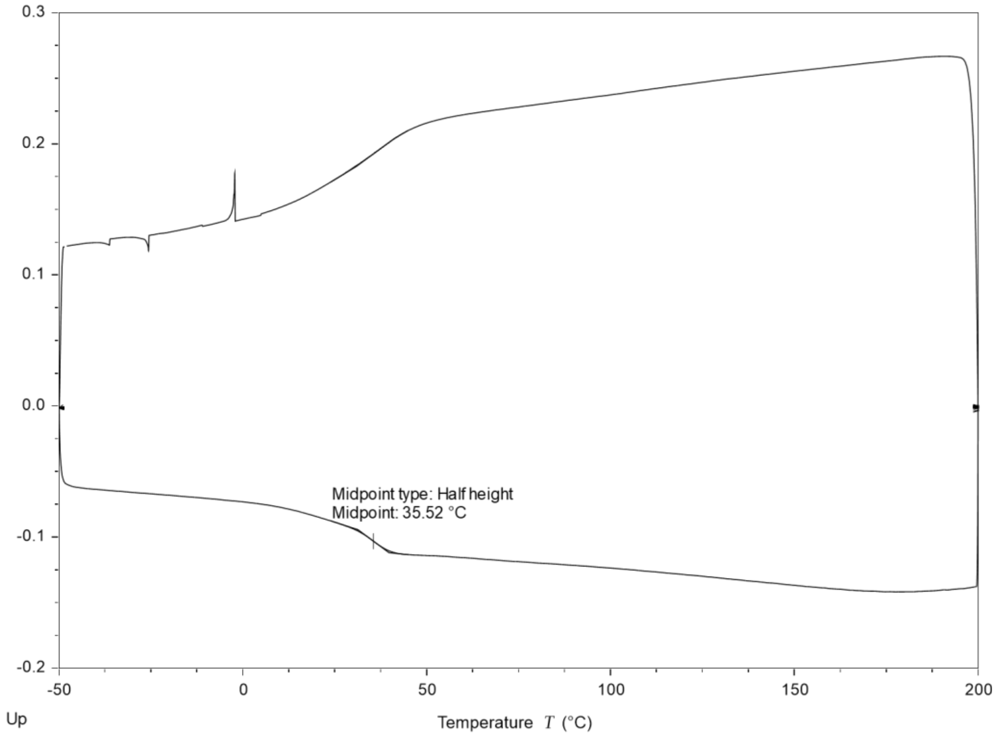
Midpoint type: Half height
Midpoint: 35.52 °C
Up
Temperature T (°C)

6

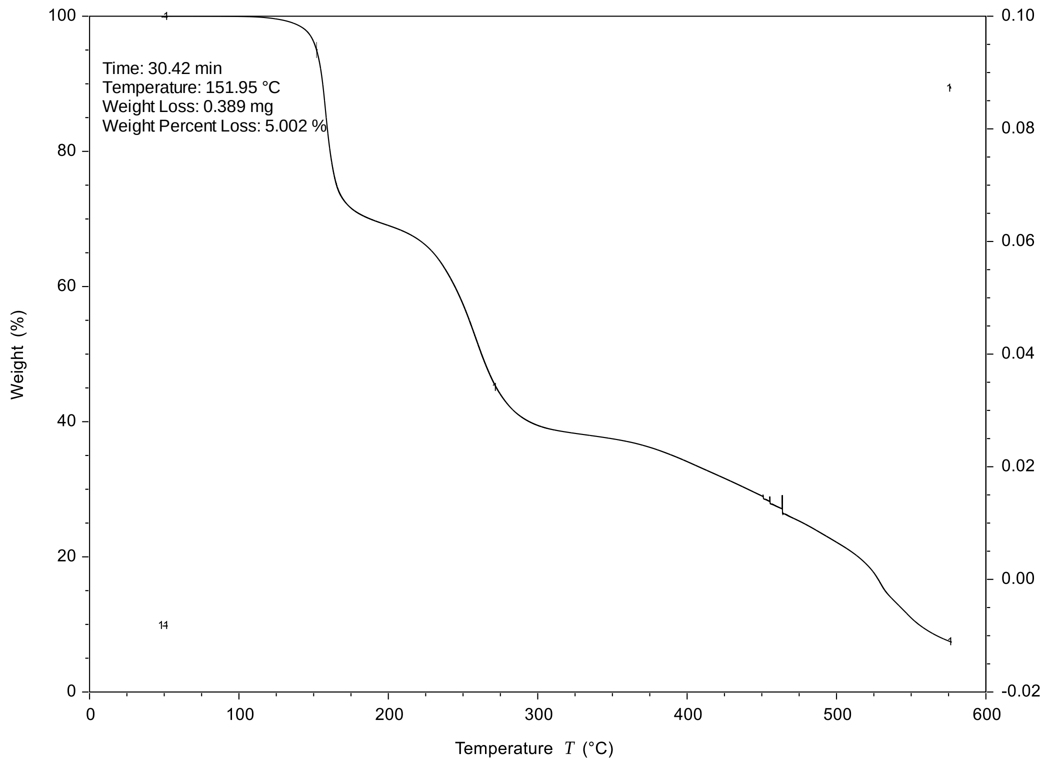
Time: 30.42 min
Temperature: 151.95 °C
Weight Loss: 0.389 mg
Weight Percent Loss: 5.002 %
Weight (%)
Temperature T (°C)
100
80
60
40
20
0
0.10
0.08
0.06
0.04
0.02
0.00
-0.02
0
100
200
300
400
500
600

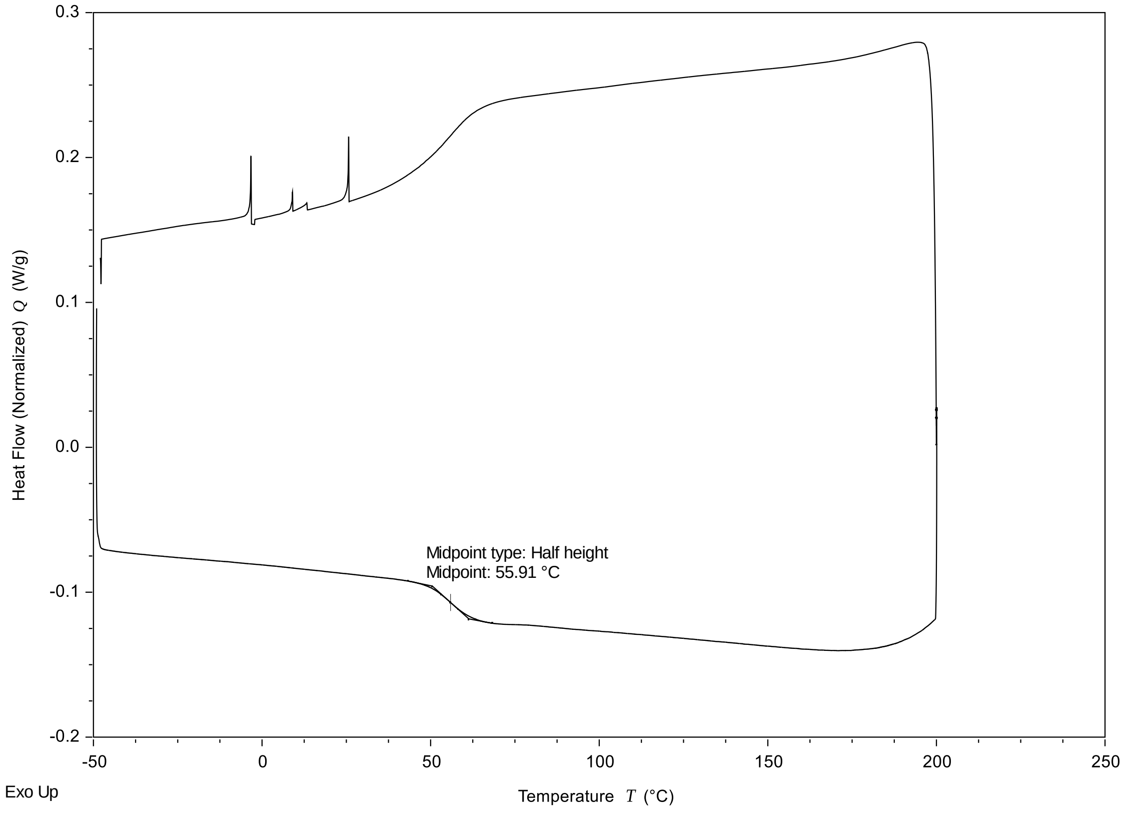
Midpoint type: Half height
Midpoint: 55.91 °C
Heat Flow (Normalized) Q (W/g)
Temperature T (°C)
Exo Up
0.3
0.2
0.1
0.0
-0.1
-0.2
-50
0
50
100
150
200
250

7

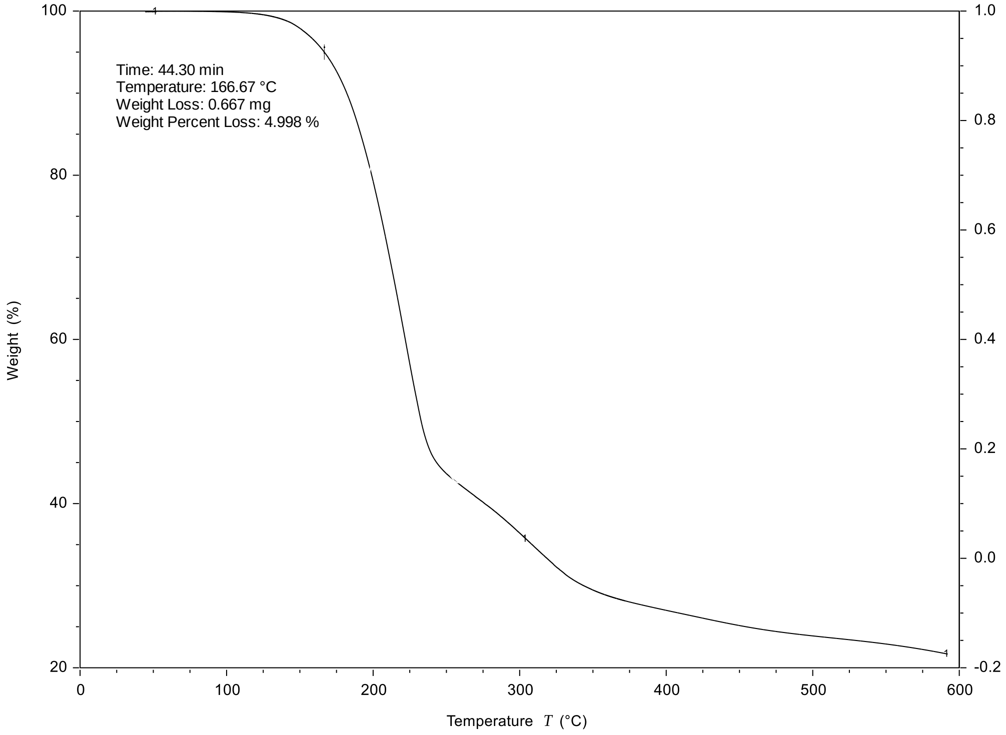
Time: 44.30 min
Temperature: 166.67 °C
Weight Loss: 0.667 mg
Weight Percent Loss: 4.998 %
Weight (%)
Temperature T (°C)
100
80
60
40
20
1.0
0.8
0.6
0.4
0.2
0.0
-0.2
0
100
200
300
400
500
600

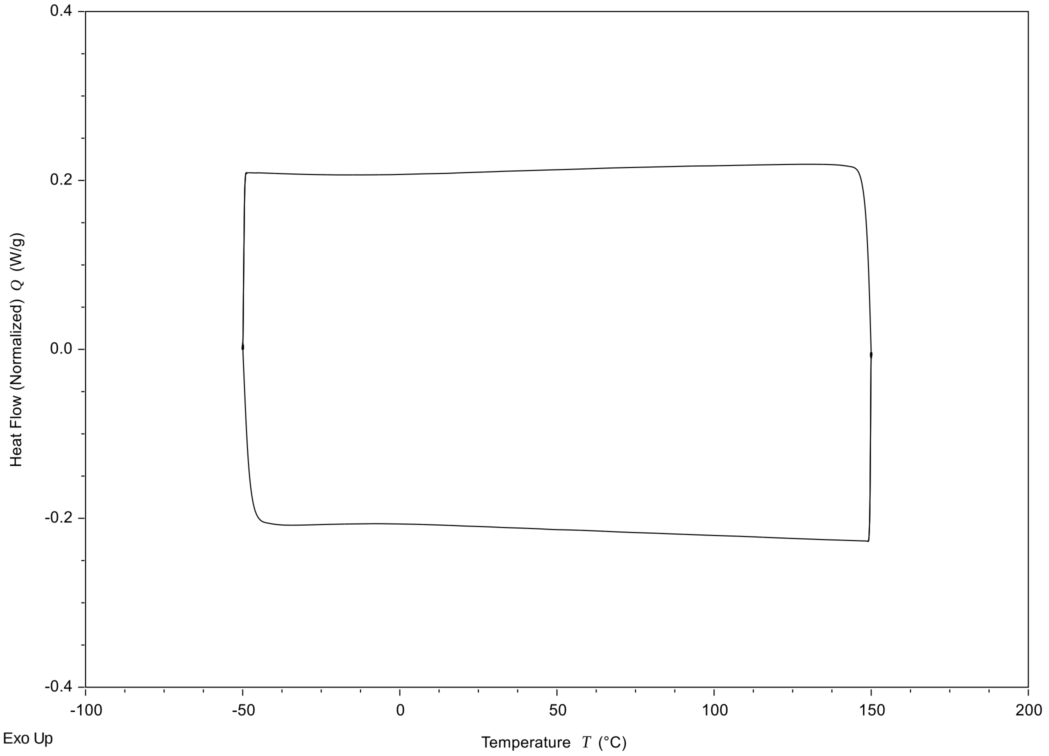
Heat Flow (Normalized) Q (W/g)
Temperature T (°C)
Exo Up
0.4
0.2
0.0
-0.2
-0.4
-100
-50
0
50
100
150
200

8

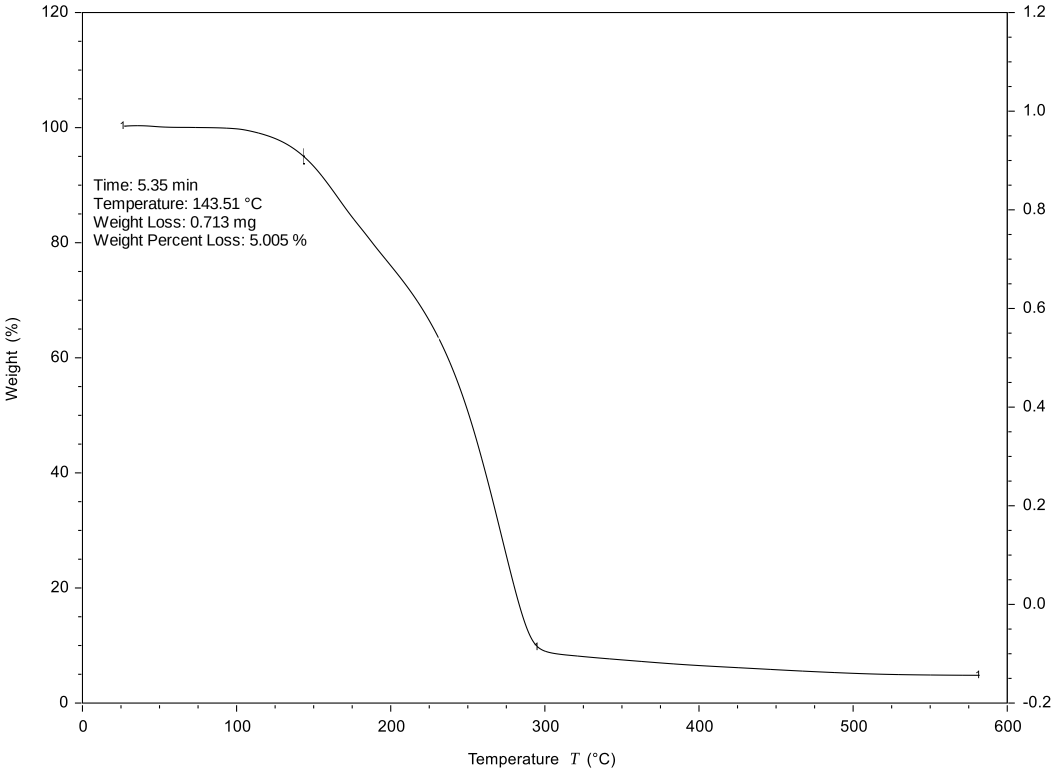
Time: 5.35 min
Temperature: 143.51 °C
Weight Loss: 0.713 mg
Weight Percent Loss: 5.005 %
Weight (%)
Temperature T (°C)

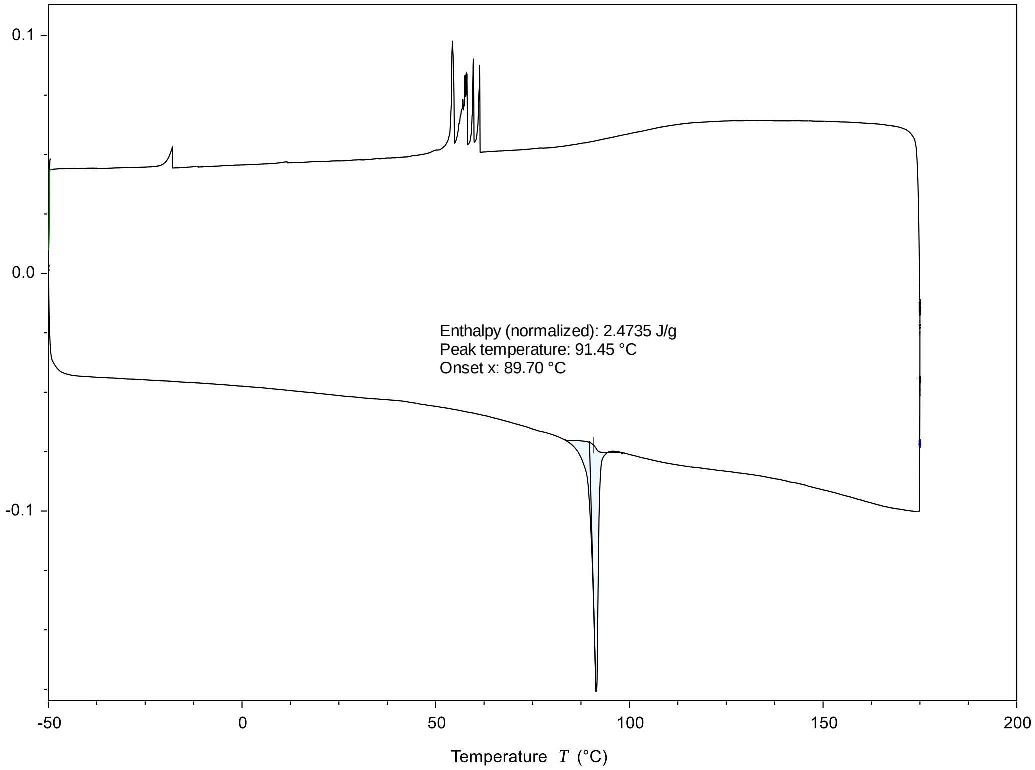
Enthalpy (normalized): 2.4735 J/g
Peak temperature: 91.45 °C
Onset x: 89.70 °C
Temperature T (°C)

9

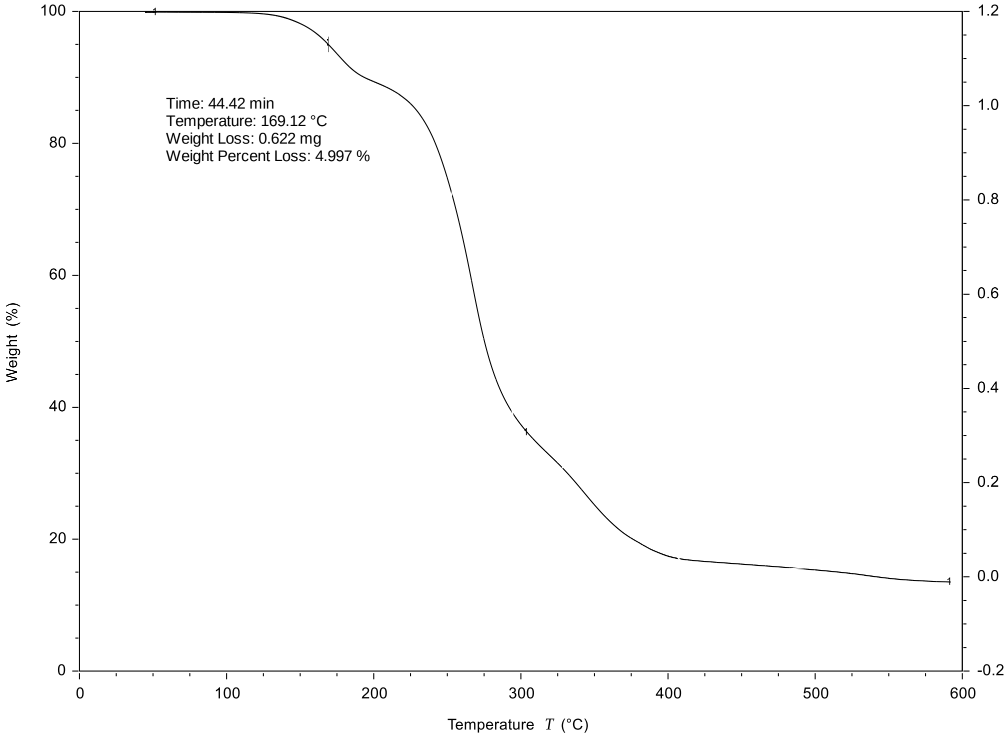
Time: 44.42 min
Temperature: 169.12 °C
Weight Loss: 0.622 mg
Weight Percent Loss: 4.997 %
Weight (%)
Temperature T (°C)
100
80
60
40
20
0
1.2
1.0
0.8
0.6
0.4
0.2
0.0
-0.2
0
100
200
300
400
500
600

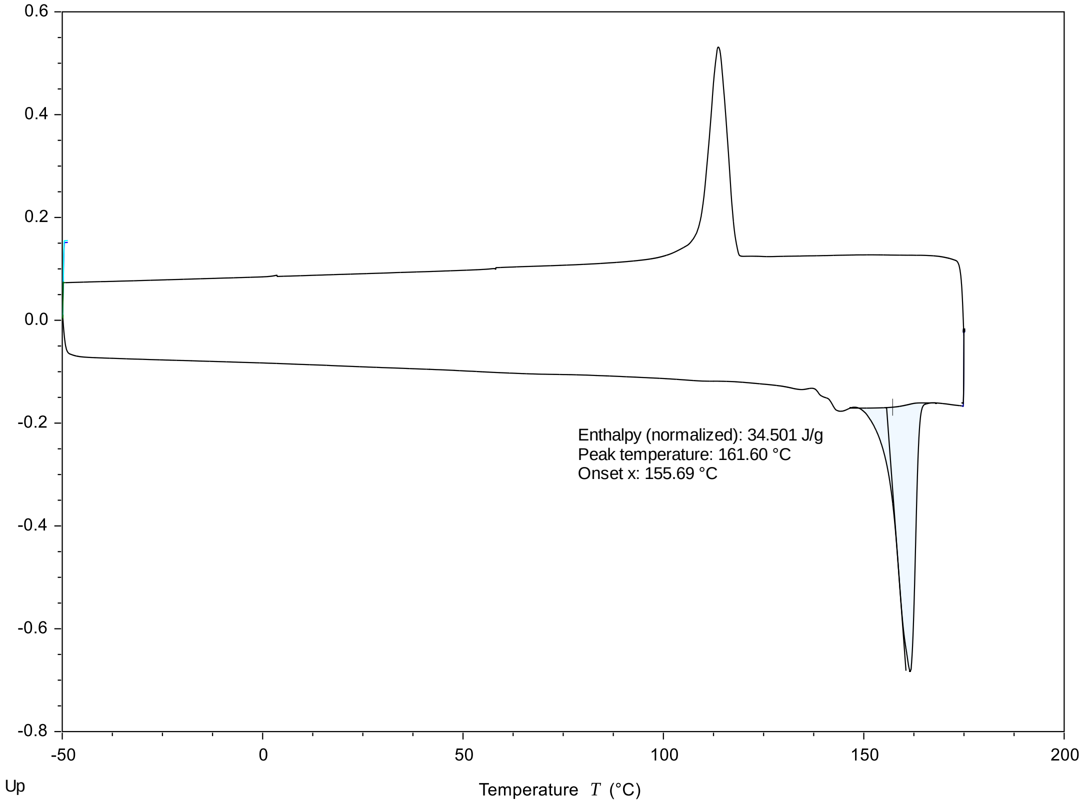
Enthalpy (normalized): 34.501 J/g
Peak temperature: 161.60 °C
Onset x: 155.69 °C
0.6
0.4
0.2
0.0
-0.2
-0.4
-0.6
-0.8
-50
0
50
100
150
200
Up
Temperature T (°C)

10

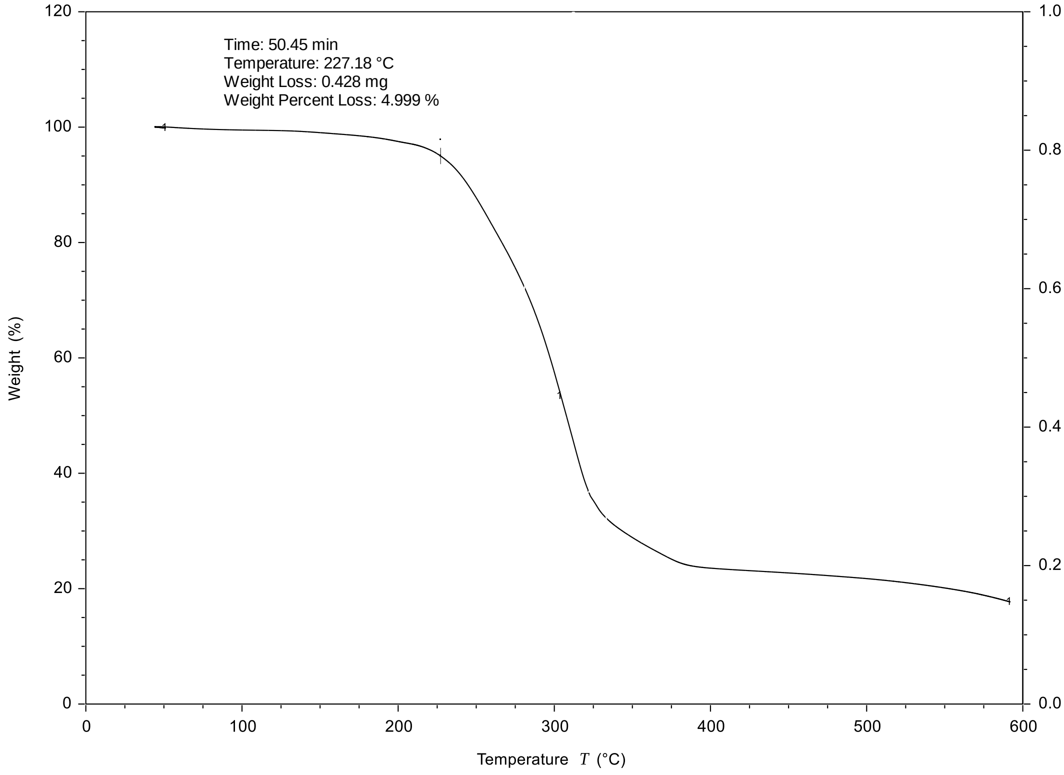
Time: 50.45 min
Temperature: 227.18 °C
Weight Loss: 0.428 mg
Weight Percent Loss: 4.999 %
Weight (%)
Temperature T (°C)

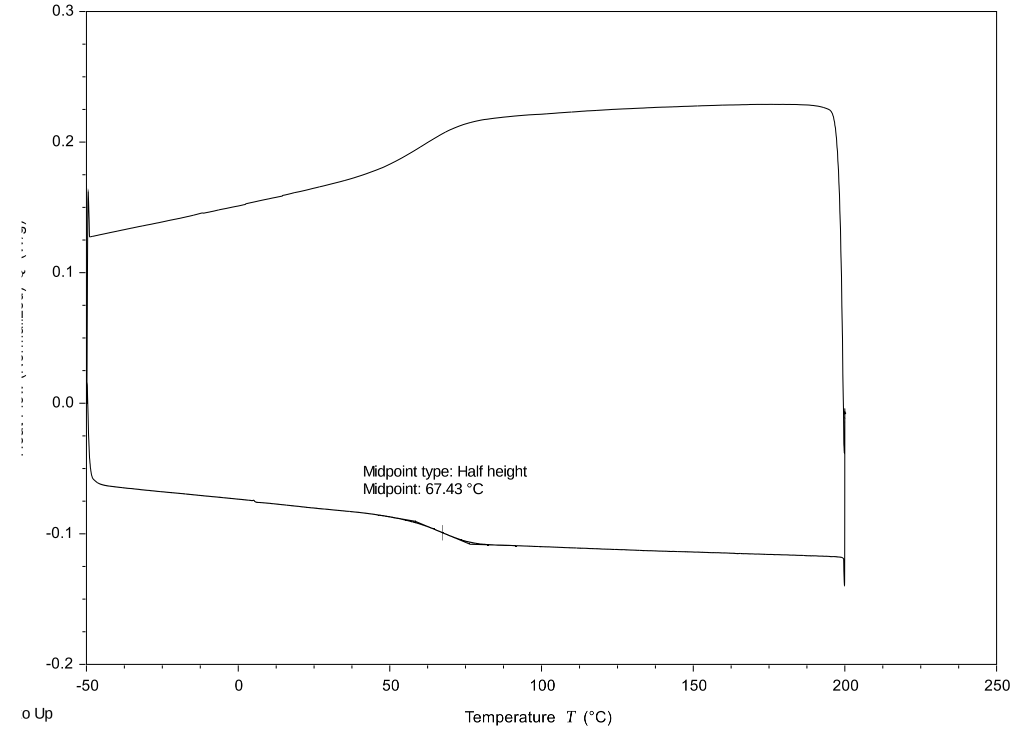
Midpoint type: Half height
Midpoint: 67.43 °C
o Up
Temperature T (°C)

11

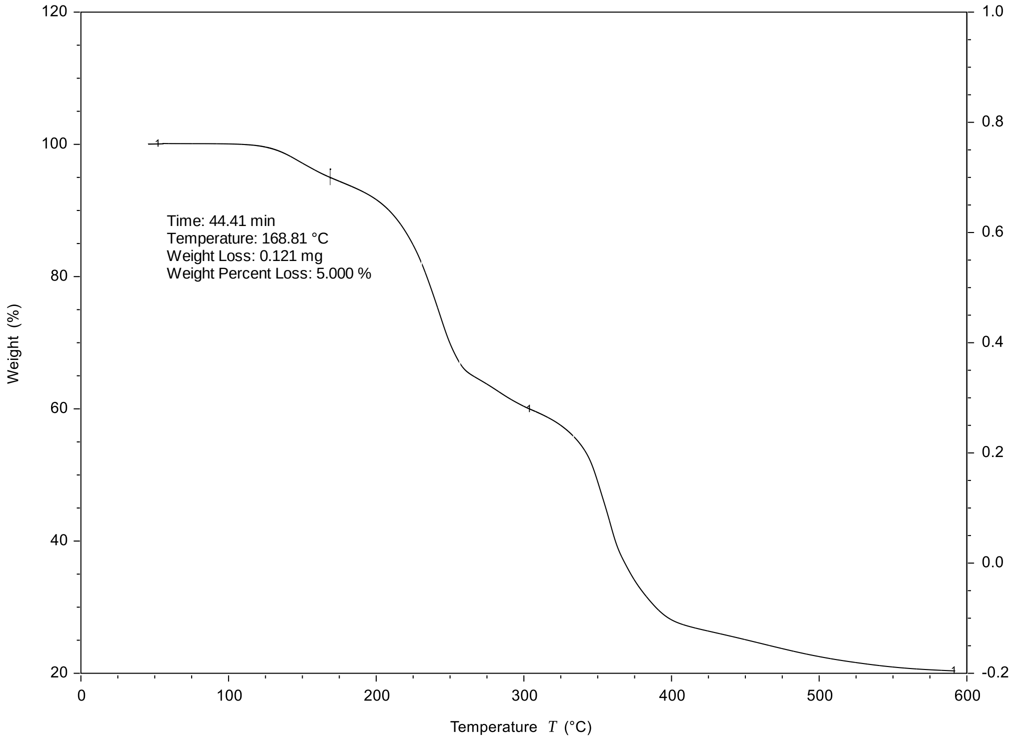
Time: 44.41 min
Temperature: 168.81 °C
Weight Loss: 0.121 mg
Weight Percent Loss: 5.000 %
Weight (%)
Temperature T (°C)
120
100
80
60
40
20
0
100
200
300
400
500
600
1.0
0.8
0.6
0.4
0.2
0.0
-0.2

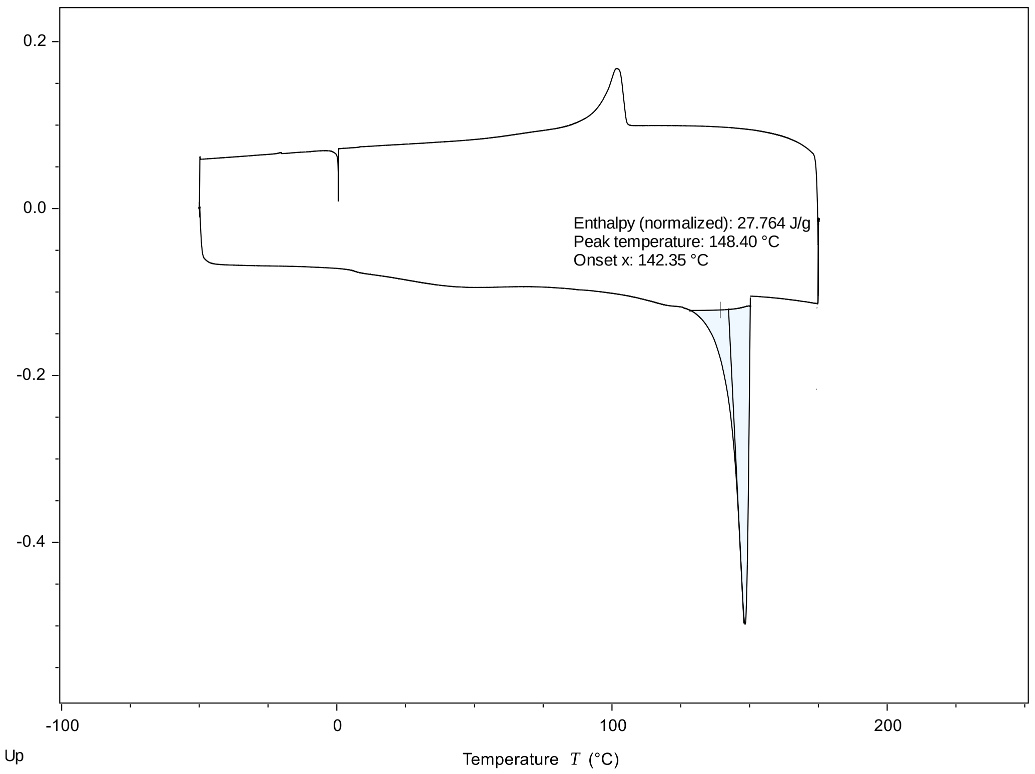
Enthalpy (normalized): 27.764 J/g
Peak temperature: 148.40 °C
Onset x: 142.35 °C
0.2
0.0
-0.2
-0.4
-100
0
100
200
Up
Temperature T (°C)

12

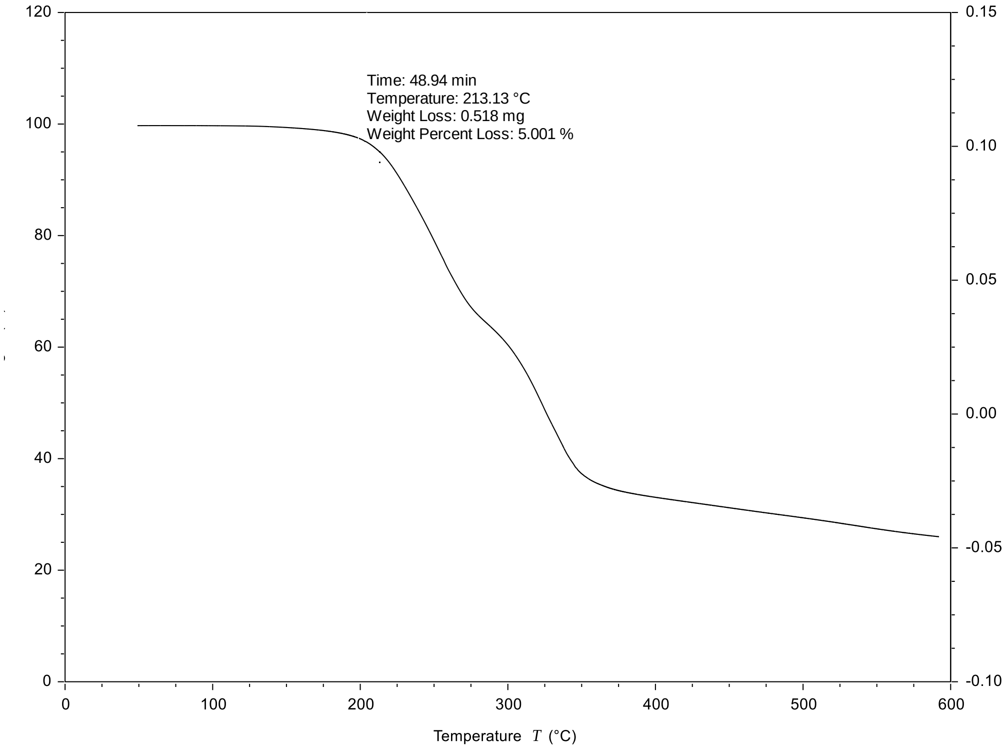

Time: 48.94 min
Temperature: 213.13 °C
Weight Loss: 0.518 mg
Weight Percent Loss: 5.001 %
Temperature T (°C)

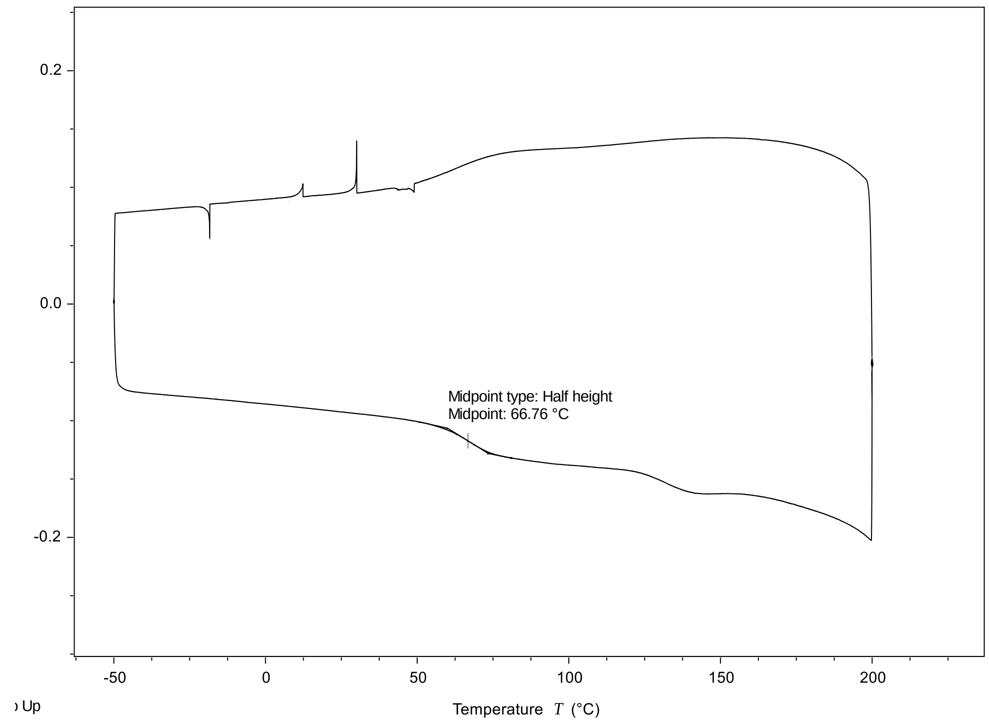

Midpoint type: Half height
Midpoint: 66.76 °C
Temperature T (°C)

Examples of Gaussian16 input files for calculations.

Compound 1

%mem=80GB
%nprocshared=8
opt freq 6-31++g(d,p) m062x pop=chelpg scf=tight

Title Card Required

0 1

C	0.51495900	0.08298400	0.00167100
C	0.20302800	0.56621000	1.44036600
C	1.15225600	0.56202100	2.45501000
C	-1.04829500	1.13279800	1.70211700
C	0.85312700	1.08057700	3.71070000
H	2.13703500	0.17478300	2.28607800
C	-1.34950700	1.63024300	2.95709900
H	-1.80367800	1.18198000	0.93723900
C	-0.39840600	1.60731100	3.96932200
H	1.60709900	1.06988700	4.47772500
H	-2.32603300	2.04362300	3.13338800
H	-0.63057500	2.00442000	4.94156800
C	1.90462000	-0.59322700	-0.13355900
C	2.87789100	-0.17133400	-1.02612000
C	2.16297600	-1.74407200	0.61854500
C	4.08551500	-0.85722600	-1.14552900
H	2.71446000	0.68471000	-1.64854500
C	3.35802300	-2.42526700	0.50401700
H	1.42131500	-2.11308300	1.30376300
C	4.33365500	-1.97927600	-0.38168200
H	4.82164600	-0.50478000	-1.84584800
H	3.52895400	-3.30400100	1.10009100
H	5.26547600	-2.50786200	-0.47474700
C	0.32082300	1.30346800	-0.92556900
C	-0.71252100	1.39894800	-1.84343000
C	1.16260200	2.40667200	-0.75232000
C	-0.87400400	2.55338700	-2.60727800
H	-1.44350500	0.62206000	-1.94567200
C	1.00617500	3.54650700	-1.51400600
H	1.93740400	2.38042700	-0.00745900
C	-0.01451100	3.62182700	-2.45707700
H	-1.69576500	2.60763600	-3.29803200
H	1.66786100	4.38109100	-1.36371300
H	-0.14515500	4.51314400	-3.04449600

N	-0.44151100	-1.01017800	-0.39592800
C	-1.44491200	-1.53389500	0.28353600
C	-0.33064300	-1.72316800	-1.58174800
H	-1.80032800	-1.19748000	1.22843700
C	-1.27013600	-2.67294800	-1.56598500
H	0.40839900	-1.49158500	-2.31425000
H	-1.52319700	-3.41444600	-2.29147900
C	-3.17254300	-3.26199100	-0.01321700
H	-3.07419900	-4.29676900	-0.31094100
H	-3.30953900	-3.20563000	1.05593100
H	-4.00502600	-2.77785600	-0.50130200
N	-1.94313000	-2.55903900	-0.36813200
Cl	-3.94007000	0.05593900	-0.36697100

Compound 2

%mem=80GB
%nprocshared=8
opt freq 6-31++g(d,p) m062x pop=chelpg scf=tight

Title Card Required

0 1

C	0.50806800	0.07842500	0.00573700
C	0.20200400	0.61841800	1.41400500
C	1.18943500	0.73493000	2.39398900
C	-1.07770100	1.13650900	1.66538400
C	0.89427000	1.32592700	3.62430000
H	2.19710500	0.38008400	2.20662500
C	-1.37075100	1.70467000	2.90051100
C	-0.38647300	1.80156500	3.88556500
H	1.67388400	1.41221000	4.37467300
H	-2.37190100	2.08291400	3.08091900
H	-0.61508800	2.25586200	4.84457700
C	1.85790100	-0.65895400	-0.10099500
C	2.78939400	-0.39952000	-1.10582700
C	2.09974400	-1.72565100	0.77628400
C	3.95448600	-1.16469300	-1.20890600
H	2.61337400	0.39501600	-1.82210400
C	3.25825400	-2.48599700	0.67702200
C	4.19707200	-2.20253300	-0.31708000
H	4.66763300	-0.94406000	-1.99679500
H	3.42780000	-3.30204100	1.37226700

H	5.10328300	-2.79423100	-0.39766400
C	0.36642500	1.27809400	-0.94370100
C	-0.75371200	1.45990900	-1.75493700
C	1.33506000	2.28875600	-0.87559900
C	-0.87119900	2.61800000	-2.52846000
H	-1.57796700	0.74987300	-1.74680400
C	1.21716900	3.43413200	-1.65284700
H	2.17977400	2.18271300	-0.19916200
C	0.11372800	3.59799800	-2.49322400
H	-1.75758800	2.74990200	-3.14047700
H	1.97903200	4.20496200	-1.59263400
H	0.01570700	4.49575900	-3.09535200
N	-0.49017300	-0.97433500	-0.36772300
C	-1.43263300	-1.53925900	0.38825500
C	-0.48151000	-1.61450500	-1.59410400
H	-1.70882500	-1.24091600	1.38596300
C	-1.43008000	-2.58223900	-1.54623400
H	0.20176400	-1.32616900	-2.37620800
H	-1.76275500	-3.28412700	-2.29338800
C	-3.22194100	-3.20746500	0.11378500
H	-3.27601900	-4.17462100	-0.38585600
H	-3.21189300	-3.35436900	1.19383300
H	-4.04830300	-2.54692800	-0.16737700
N	-1.98378600	-2.54617400	-0.28531100
Cl	-3.74492900	0.03496800	-0.43897700
H	1.38144889	-1.95617899	1.53510478
Cl	-2.33689831	1.05856491	0.43821374

Compound 3

%mem=80GB
%nprocshared=8
opt freq 6-31++g(d,p) m062x pop=chelpg scf=tight

Title Card Required

0 1

C	0.50806800	0.07842500	0.00573700
C	0.20200400	0.61841800	1.41400500
C	1.18943500	0.73493000	2.39398900
C	-1.07770100	1.13650900	1.66538400
C	0.89427000	1.32592700	3.62430000
H	2.19710500	0.38008400	2.20662500
C	-1.37075100	1.70467000	2.90051100
C	-0.38647300	1.80156500	3.88556500

H	1.67388400	1.41221000	4.37467300
H	-2.37190100	2.08291400	3.08091900
H	-0.61508800	2.25586200	4.84457700
C	1.85790100	-0.65895400	-0.10099500
C	2.78939400	-0.39952000	-1.10582700
C	2.09974400	-1.72565100	0.77628400
C	3.95448600	-1.16469300	-1.20890600
H	2.61337400	0.39501600	-1.82210400
C	3.25825400	-2.48599700	0.67702200
C	4.19707200	-2.20253300	-0.31708000
H	4.66763300	-0.94406000	-1.99679500
H	3.42780000	-3.30204100	1.37226700
H	5.10328300	-2.79423100	-0.39766400
C	0.36642500	1.27809400	-0.94370100
C	-0.75371200	1.45990900	-1.75493700
C	1.33506000	2.28875600	-0.87559900
C	-0.87119900	2.61800000	-2.52846000
H	-1.57796700	0.74987300	-1.74680400
C	1.21716900	3.43413200	-1.65284700
H	2.17977400	2.18271300	-0.19916200
C	0.11372800	3.59799800	-2.49322400
H	-1.75758800	2.74990200	-3.14047700
H	1.97903200	4.20496200	-1.59263400
H	0.01570700	4.49575900	-3.09535200
N	-0.49017300	-0.97433500	-0.36772300
C	-1.43263300	-1.53925900	0.38825500
C	-0.48151000	-1.61450500	-1.59410400
H	-1.70882500	-1.24091600	1.38596300
C	-1.43008000	-2.58223900	-1.54623400
H	0.20176400	-1.32616900	-2.37620800
C	-3.22194100	-3.20746500	0.11378500
H	-3.27601900	-4.17462100	-0.38585600
H	-3.21189300	-3.35436900	1.19383300
H	-4.04830300	-2.54692800	-0.16737700
N	-1.98378600	-2.54617400	-0.28531100
Cl	-3.74492900	0.03496800	-0.43897700
H	1.38144889	-1.95617899	1.53510478
Cl	-2.33689831	1.05856491	0.43821374
N	-1.88383033	-3.53957547	-2.56531080
O	-3.22943927	-3.42607177	-2.72672174
O	-1.58010342	-4.80389370	-2.16675167

Compound 4

%mem=80GB

%nprocshared=8
opt freq 6-31++g(d,p) m062x pop=chelpg scf=tight

Title Card Required

0 1

C	0.50806800	0.07842500	0.00573700
C	0.20200400	0.61841800	1.41400500
C	1.18943500	0.73493000	2.39398900
C	-1.07770100	1.13650900	1.66538400
C	0.89427000	1.32592700	3.62430000
H	2.19710500	0.38008400	2.20662500
C	-1.37075100	1.70467000	2.90051100
H	-1.85923800	1.08813200	0.90372500
C	-0.38647300	1.80156500	3.88556500
H	1.67388400	1.41221000	4.37467300
H	-2.37190100	2.08291400	3.08091900
H	-0.61508800	2.25586200	4.84457700
C	1.85790100	-0.65895400	-0.10099500
C	2.78939400	-0.39952000	-1.10582700
C	2.09974400	-1.72565100	0.77628400
C	3.95448600	-1.16469300	-1.20890600
H	2.61337400	0.39501600	-1.82210400
C	3.25825400	-2.48599700	0.67702200
H	1.36967400	-1.95995800	1.54754400
C	4.19707200	-2.20253300	-0.31708000
H	4.66763300	-0.94406000	-1.99679500
H	3.42780000	-3.30204100	1.37226700
C	0.36642500	1.27809400	-0.94370100
C	-0.75371200	1.45990900	-1.75493700
C	1.33506000	2.28875600	-0.87559900
C	-0.87119900	2.61800000	-2.52846000
H	-1.57796700	0.74987300	-1.74680400
C	1.21716900	3.43413200	-1.65284700
H	2.17977400	2.18271300	-0.19916200
C	0.11372800	3.59799800	-2.49322400
H	-1.75758800	2.74990200	-3.14047700
H	0.01570700	4.49575900	-3.09535200
N	-0.49017300	-0.97433500	-0.36772300
C	-1.43263300	-1.53925900	0.38825500
C	-0.48151000	-1.61450500	-1.59410400
H	-1.70882500	-1.24091600	1.38596300
C	-1.43008000	-2.58223900	-1.54623400
H	0.20176400	-1.32616900	-2.37620800
H	-1.76275500	-3.28412700	-2.29338800
C	-3.22194100	-3.20746500	0.11378500

H	-3.27601900	-4.17462100	-0.38585600
H	-3.21189300	-3.35436900	1.19383300
H	-4.04830300	-2.54692800	-0.16737700
N	-1.98378600	-2.54617400	-0.28531100
Cl	-3.74492900	0.03496800	-0.43897700
O	2.22085089	4.44962706	-1.57352211
O	5.39113199	-2.98217804	-0.42326071
C	1.95534268	5.46047325	-2.54953204
H	1.02109637	5.93298181	-2.32858073
H	2.73768672	6.19014765	-2.52920751
H	1.90857806	5.01465737	-3.52110873
C	6.43359887	-2.36454803	0.33614430
H	7.32187750	-2.95747998	0.27062511
H	6.62765810	-1.38712280	-0.05357475
H	6.13128831	-2.28689876	1.35960848

Compound 5

%mem=80GB
%nprocshared=8
opt freq 6-31++g(d,p) m062x pop=chelpg scf=tight

Title Card Required

0 1

C	0.50806800	0.07842500	0.00573700
C	0.20200400	0.61841800	1.41400500
C	1.18943500	0.73493000	2.39398900
C	-1.07770100	1.13650900	1.66538400
C	0.89427000	1.32592700	3.62430000
H	2.19710500	0.38008400	2.20662500
C	-1.37075100	1.70467000	2.90051100
H	-1.85923800	1.08813200	0.90372500
C	-0.38647300	1.80156500	3.88556500
H	1.67388400	1.41221000	4.37467300
H	-2.37190100	2.08291400	3.08091900
C	1.85790100	-0.65895400	-0.10099500
C	2.78939400	-0.39952000	-1.10582700
C	2.09974400	-1.72565100	0.77628400
C	3.95448600	-1.16469300	-1.20890600
H	2.61337400	0.39501600	-1.82210400
C	3.25825400	-2.48599700	0.67702200
H	1.36967400	-1.95995800	1.54754400
C	4.19707200	-2.20253300	-0.31708000
H	4.66763300	-0.94406000	-1.99679500

H	3.42780000	-3.30204100	1.37226700
C	0.36642500	1.27809400	-0.94370100
C	-0.75371200	1.45990900	-1.75493700
C	1.33506000	2.28875600	-0.87559900
C	-0.87119900	2.61800000	-2.52846000
H	-1.57796700	0.74987300	-1.74680400
C	1.21716900	3.43413200	-1.65284700
H	2.17977400	2.18271300	-0.19916200
C	0.11372800	3.59799800	-2.49322400
H	-1.75758800	2.74990200	-3.14047700
H	0.01570700	4.49575900	-3.09535200
N	-0.49017300	-0.97433500	-0.36772300
C	-1.43263300	-1.53925900	0.38825500
C	-0.48151000	-1.61450500	-1.59410400
H	-1.70882500	-1.24091600	1.38596300
C	-1.43008000	-2.58223900	-1.54623400
H	0.20176400	-1.32616900	-2.37620800
H	-1.76275500	-3.28412700	-2.29338800
C	-3.22194100	-3.20746500	0.11378500
H	-3.27601900	-4.17462100	-0.38585600
H	-3.21189300	-3.35436900	1.19383300
H	-4.04830300	-2.54692800	-0.16737700
N	-1.98378600	-2.54617400	-0.28531100
Cl	-3.74492900	0.03496800	-0.43897700
O	2.22085089	4.44962706	-1.57352211
O	5.39113199	-2.98217804	-0.42326071
C	1.95534268	5.46047325	-2.54953204
H	1.02109637	5.93298181	-2.32858073
H	2.73768672	6.19014765	-2.52920751
H	1.90857806	5.01465737	-3.52110873
C	6.43359887	-2.36454803	0.33614430
H	7.32187750	-2.95747998	0.27062511
H	6.62765810	-1.38712280	-0.05357475
H	6.13128831	-2.28689876	1.35960848
O	-0.68763679	2.40002883	5.14891033
C	-1.14312959	1.39388692	6.05726311
H	-1.33979538	1.83711579	7.01108214
H	-2.04022470	0.95077407	5.67809117
H	-0.39019178	0.64092376	6.16229230

Compound 6

%mem=80GB
%nprocshared=8
opt freq 6-31++g(d,p) m062x pop=chelpg scf=tight

Title Card Required

0 1

C	0.50806800	0.07842500	0.00573700
C	0.20200400	0.61841800	1.41400500
C	1.18943500	0.73493000	2.39398900
C	-1.07770100	1.13650900	1.66538400
C	0.89427000	1.32592700	3.62430000
H	2.19710500	0.38008400	2.20662500
C	-1.37075100	1.70467000	2.90051100
H	-1.85923800	1.08813200	0.90372500
C	-0.38647300	1.80156500	3.88556500
H	1.67388400	1.41221000	4.37467300
H	-2.37190100	2.08291400	3.08091900
C	1.85790100	-0.65895400	-0.10099500
C	2.78939400	-0.39952000	-1.10582700
C	2.09974400	-1.72565100	0.77628400
C	3.95448600	-1.16469300	-1.20890600
H	2.61337400	0.39501600	-1.82210400
C	3.25825400	-2.48599700	0.67702200
H	1.36967400	-1.95995800	1.54754400
C	4.19707200	-2.20253300	-0.31708000
H	4.66763300	-0.94406000	-1.99679500
H	3.42780000	-3.30204100	1.37226700
C	0.36642500	1.27809400	-0.94370100
C	-0.75371200	1.45990900	-1.75493700
C	1.33506000	2.28875600	-0.87559900
C	-0.87119900	2.61800000	-2.52846000
H	-1.57796700	0.74987300	-1.74680400
C	1.21716900	3.43413200	-1.65284700
H	2.17977400	2.18271300	-0.19916200
C	0.11372800	3.59799800	-2.49322400
H	-1.75758800	2.74990200	-3.14047700
H	0.01570700	4.49575900	-3.09535200
N	-0.49017300	-0.97433500	-0.36772300
C	-1.43263300	-1.53925900	0.38825500
C	-0.48151000	-1.61450500	-1.59410400
C	-1.43008000	-2.58223900	-1.54623400
H	0.20176400	-1.32616900	-2.37620800
H	-1.76275500	-3.28412700	-2.29338800
C	-3.22194100	-3.20746500	0.11378500
H	-3.27601900	-4.17462100	-0.38585600
H	-3.21189300	-3.35436900	1.19383300
H	-4.04830300	-2.54692800	-0.16737700
N	-1.98378600	-2.54617400	-0.28531100

Cl	-3.74492900	0.03496800	-0.43897700
O	2.22085089	4.44962706	-1.57352211
O	5.39113199	-2.98217804	-0.42326071
C	1.95534268	5.46047325	-2.54953204
H	1.02109637	5.93298181	-2.32858073
H	2.73768672	6.19014765	-2.52920751
H	1.90857806	5.01465737	-3.52110873
C	6.43359887	-2.36454803	0.33614430
H	7.32187750	-2.95747998	0.27062511
H	6.62765810	-1.38712280	-0.05357475
H	6.13128831	-2.28689876	1.35960848
O	-0.68763679	2.40002883	5.14891033
C	-1.14312959	1.39388692	6.05726311
H	-1.33979538	1.83711579	7.01108214
H	-2.04022470	0.95077407	5.67809117
H	-0.39019178	0.64092376	6.16229230
C	-1.82742615	-1.11280286	1.81439438
H	-0.94385590	-0.92695362	2.38855783
H	-2.41771825	-0.22148442	1.76959839
H	-2.39500854	-1.89366677	2.27591555

Compound 7

%mem=80GB
%nprocshared=8
opt freq 6-31++g(d,p) m062x pop=chelpg scf=tight

Title Card Required

0 1

C	0.50806800	0.07842500	0.00573700
C	0.20200400	0.61841800	1.41400500
C	1.18943500	0.73493000	2.39398900
C	-1.07770100	1.13650900	1.66538400
C	0.89427000	1.32592700	3.62430000
H	2.19710500	0.38008400	2.20662500
C	-1.37075100	1.70467000	2.90051100
H	-1.85923800	1.08813200	0.90372500
C	-0.38647300	1.80156500	3.88556500
H	1.67388400	1.41221000	4.37467300
H	-2.37190100	2.08291400	3.08091900
H	-0.61508800	2.25586200	4.84457700
C	1.85790100	-0.65895400	-0.10099500
C	2.78939400	-0.39952000	-1.10582700
C	2.09974400	-1.72565100	0.77628400

C	3.95448600	-1.16469300	-1.20890600
H	2.61337400	0.39501600	-1.82210400
C	3.25825400	-2.48599700	0.67702200
H	1.36967400	-1.95995800	1.54754400
C	4.19707200	-2.20253300	-0.31708000
H	4.66763300	-0.94406000	-1.99679500
H	3.42780000	-3.30204100	1.37226700
H	5.10328300	-2.79423100	-0.39766400
C	0.36642500	1.27809400	-0.94370100
C	-0.75371200	1.45990900	-1.75493700
C	1.33506000	2.28875600	-0.87559900
C	-0.87119900	2.61800000	-2.52846000
H	-1.57796700	0.74987300	-1.74680400
C	1.21716900	3.43413200	-1.65284700
H	2.17977400	2.18271300	-0.19916200
C	0.11372800	3.59799800	-2.49322400
H	-1.75758800	2.74990200	-3.14047700
H	1.97903200	4.20496200	-1.59263400
H	0.01570700	4.49575900	-3.09535200
Cl	-3.74492900	0.03496800	-0.43897700
S	-0.67802493	-1.17244647	-0.43800180
C	-0.28584850	-1.81806923	-2.04976137
C	-1.46210638	-2.88425647	-3.84997698
C	-0.71984603	-3.82516636	-3.29246855
H	-0.48740781	-3.81393445	-1.15398887
H	-2.04852888	-3.00785504	-4.73773383
H	-0.61467208	-4.82545305	-3.66076950
N	-0.04161737	-3.34886685	-2.02743195
N	-1.43011694	-1.58876474	-3.07032894
C	-2.71713418	-1.35260032	-2.40046851
H	-2.81678210	-2.02331967	-1.57275731
H	-2.75586225	-0.34302369	-2.04811190
H	-3.51556627	-1.51955567	-3.09295108

Compound 8

%mem=80GB
%nprocshared=8
opt freq 6-31++g(d,p) m062x pop=chelpg scf=tight

Title Card Required

0 1

C	0.50806800	0.07842500	0.00573700
C	0.20200400	0.61841800	1.41400500

C	1.18943500	0.73493000	2.39398900
C	-1.07770100	1.13650900	1.66538400
C	0.89427000	1.32592700	3.62430000
H	2.19710500	0.38008400	2.20662500
C	-1.37075100	1.70467000	2.90051100
H	-1.85923800	1.08813200	0.90372500
C	-0.38647300	1.80156500	3.88556500
H	1.67388400	1.41221000	4.37467300
H	-2.37190100	2.08291400	3.08091900
H	-0.61508800	2.25586200	4.84457700
C	1.85790100	-0.65895400	-0.10099500
C	2.78939400	-0.39952000	-1.10582700
C	2.09974400	-1.72565100	0.77628400
C	3.95448600	-1.16469300	-1.20890600
H	2.61337400	0.39501600	-1.82210400
C	3.25825400	-2.48599700	0.67702200
C	4.19707200	-2.20253300	-0.31708000
H	4.66763300	-0.94406000	-1.99679500
H	3.42780000	-3.30204100	1.37226700
H	5.10328300	-2.79423100	-0.39766400
C	0.36642500	1.27809400	-0.94370100
C	-0.75371200	1.45990900	-1.75493700
C	1.33506000	2.28875600	-0.87559900
C	-0.87119900	2.61800000	-2.52846000
H	-1.57796700	0.74987300	-1.74680400
C	1.21716900	3.43413200	-1.65284700
H	2.17977400	2.18271300	-0.19916200
C	0.11372800	3.59799800	-2.49322400
H	-1.75758800	2.74990200	-3.14047700
H	1.97903200	4.20496200	-1.59263400
H	0.01570700	4.49575900	-3.09535200
Cl	-3.74492900	0.03496800	-0.43897700
S	-0.67802493	-1.17244647	-0.43800180
C	-0.28584850	-1.81806923	-2.04976137
C	-1.46210638	-2.88425647	-3.84997698
C	-0.71984603	-3.82516636	-3.29246855
H	-0.48740781	-3.81393445	-1.15398887
H	-2.04852888	-3.00785504	-4.73773383
H	-0.61467208	-4.82545305	-3.66076950
N	-0.04161737	-3.34886685	-2.02743195
N	-1.43011694	-1.58876474	-3.07032894
C	-2.71713418	-1.35260032	-2.40046851
H	-2.81678210	-2.02331967	-1.57275731
H	-2.75586225	-0.34302369	-2.04811190
H	-3.51556627	-1.51955567	-3.09295108
Cl	0.91824924	-2.10483723	2.02443781

Compound 9

```
%mem=80GB
%nprocshared=8
# opt freq 6-31++g(d,p) m062x pop=chelpg scf=tight

Title Card Required

0 1
 C                  0.50806800    0.07842500    0.00573700
 C                  0.20200400    0.61841800    1.41400500
 C                  1.18943500    0.73493000    2.39398900
 C                 -1.07770100    1.13650900    1.66538400
 C                  0.89427000    1.32592700    3.62430000
 H                  2.19710500    0.38008400    2.20662500
 C                 -1.37075100    1.70467000    2.90051100
 H                 -1.85923800    1.08813200    0.90372500
 C                 -0.38647300    1.80156500    3.88556500
 H                  1.67388400    1.41221000    4.37467300
 H                 -2.37190100    2.08291400    3.08091900
 H                 -0.61508800    2.25586200    4.84457700
 C                  1.85790100   -0.65895400   -0.10099500
 C                  2.78939400   -0.39952000   -1.10582700
 C                  2.09974400   -1.72565100    0.77628400
 C                  3.95448600   -1.16469300   -1.20890600
 H                  2.61337400    0.39501600   -1.82210400
 C                  3.25825400   -2.48599700    0.67702200
 H                  1.36967400   -1.95995800    1.54754400
 C                  4.19707200   -2.20253300   -0.31708000
 H                  4.66763300   -0.94406000   -1.99679500
 H                  3.42780000   -3.30204100    1.37226700
 H                  5.10328300   -2.79423100   -0.39766400
 C                  0.36642500    1.27809400   -0.94370100
 C                 -0.75371200    1.45990900   -1.75493700
 C                  1.33506000    2.28875600   -0.87559900
 C                 -0.87119900    2.61800000   -2.52846000
 H                 -1.57796700    0.74987300   -1.74680400
 C                  1.21716900    3.43413200   -1.65284700
 H                  2.17977400    2.18271300   -0.19916200
 C                  0.11372800    3.59799800   -2.49322400
 H                 -1.75758800    2.74990200   -3.14047700
 H                  1.97903200    4.20496200   -1.59263400
 H                  0.01570700    4.49575900   -3.09535200
 Cl                -3.74492900    0.03496800   -0.43897700
```

N	-0.54688803	-0.88575673	-0.33824172
C	-1.36971494	-1.39066992	0.60142881
C	-0.73139310	-1.30153498	-1.60653823
C	-2.39294134	-2.31480931	0.32164350
H	-1.19677274	-1.03324768	1.63217856
C	-1.72601872	-2.22185148	-1.98506452
H	-0.04334596	-0.87211103	-2.35650903
C	-2.56955419	-2.73466989	-0.99804079
H	-3.03420805	-2.69334623	1.12776754
H	-1.83059084	-2.52521536	-3.03454114
N	-3.62543218	-3.69789838	-1.34186128
H	-3.34448390	-4.22931745	-2.14102450
H	-3.77918961	-4.31384637	-0.56922506

Compound 10

%mem=80GB
%nprocshared=8
opt freq 6-31++g(d,p) m062x pop=chelpg scf=tight

Title Card Required

0 1

C	-0.65891000	0.05227800	0.00077900
C	-0.76512000	-0.89332500	1.21161000
C	-2.00490100	-1.14770500	1.80245600
C	0.35021600	-1.64617100	1.60561200
C	-2.12348700	-2.10664300	2.80811400
H	-2.88888700	-0.60904700	1.47492500
C	0.22604100	-2.59596200	2.61544700
H	1.32077800	-1.51370900	1.11976800
C	-1.00687100	-2.82584100	3.22564300
H	-3.09358700	-2.29032100	3.25915100
H	1.10265300	-3.16428000	2.90951300
H	-1.09929700	-3.57040000	4.01008600
C	-1.65855200	1.22176100	-0.00488700
C	-2.07486700	1.80070800	-1.20843500
C	-2.02060300	1.84810100	1.19316100
C	-2.86021900	2.95192300	-1.21387900
H	-1.79246500	1.34885200	-2.15421300
C	-2.80573600	2.99867600	1.18997200
C	-3.23397200	3.55253000	-0.01437700
H	-3.17747700	3.37811800	-2.16029300
H	-3.08027400	3.46166600	2.13245200
H	-3.84832600	4.44711300	-0.01790300
C	-0.76107700	-0.89784500	-1.20709700

C	0.36388900	-1.60415000	-1.65376400
C	-2.01620800	-1.20640400	-1.73928900
C	0.22948500	-2.56158100	-2.65673900
H	1.35300600	-1.43958300	-1.21784700
C	-2.14354500	-2.16901800	-2.73882900
H	-2.90503500	-0.70529500	-1.36749000
C	-1.01892100	-2.84300300	-3.20891600
H	1.11362700	-3.09557100	-2.99005500
H	-3.12569500	-2.39276400	-3.14326700
H	-1.11726400	-3.59227600	-3.98810200
Cl	3.57929200	-1.17651100	-0.00775300
N	0.69158500	0.74115900	0.00419400
C	1.27608000	1.10550700	1.17130100
C	1.25340900	1.14483700	-1.16095400
C	2.50209000	1.70228400	1.20679000
H	0.74515300	0.84577400	2.07672900
C	2.48028400	1.73990100	-1.20050000
H	0.70168100	0.91944700	-2.06339100
C	3.20383900	1.90480500	-0.00029200
H	2.96856300	1.90194800	2.16361100
H	2.92805800	1.97241600	-2.15888700
N	4.49544500	2.30014900	-0.00511500
H	5.01184400	2.12674000	-0.85639600
H	5.02652100	2.09559800	0.83019500
Cl	-1.47646544	1.17279386	2.72465523

Compound 11

%mem=80GB
%nprocshared=8
opt freq 6-31++g(d,p) m062x pop=chelpg scf=tight

Title Card Required

0 1

C	-0.65891000	0.05227800	0.00077900
C	-0.76512000	-0.89332500	1.21161000
C	-2.00490100	-1.14770500	1.80245600
C	0.35021600	-1.64617100	1.60561200
C	-2.12348700	-2.10664300	2.80811400
H	-2.88888700	-0.60904700	1.47492500
C	0.22604100	-2.59596200	2.61544700
H	1.32077800	-1.51370900	1.11976800
C	-1.00687100	-2.82584100	3.22564300
H	-3.09358700	-2.29032100	3.25915100

H	1.10265300	-3.16428000	2.90951300
C	-1.65855200	1.22176100	-0.00488700
C	-2.07486700	1.80070800	-1.20843500
C	-2.02060300	1.84810100	1.19316100
C	-2.86021900	2.95192300	-1.21387900
H	-1.79246500	1.34885200	-2.15421300
C	-2.80573600	2.99867600	1.18997200
C	-3.23397200	3.55253000	-0.01437700
H	-3.17747700	3.37811800	-2.16029300
H	-3.08027400	3.46166600	2.13245200
H	-3.84832600	4.44711300	-0.01790300
C	-0.76107700	-0.89784500	-1.20709700
C	0.36388900	-1.60415000	-1.65376400
C	-2.01620800	-1.20640400	-1.73928900
C	0.22948500	-2.56158100	-2.65673900
H	1.35300600	-1.43958300	-1.21784700
C	-2.14354500	-2.16901800	-2.73882900
H	-2.90503500	-0.70529500	-1.36749000
C	-1.01892100	-2.84300300	-3.20891600
H	1.11362700	-3.09557100	-2.99005500
H	-3.12569500	-2.39276400	-3.14326700
Cl	3.57929200	-1.17651100	-0.00775300
N	0.69158500	0.74115900	0.00419400
C	1.27608000	1.10550700	1.17130100
C	1.25340900	1.14483700	-1.16095400
C	2.50209000	1.70228400	1.20679000
H	0.74515300	0.84577400	2.07672900
C	2.48028400	1.73990100	-1.20050000
H	0.70168100	0.91944700	-2.06339100
C	3.20383900	1.90480500	-0.00029200
H	2.96856300	1.90194800	2.16361100
H	2.92805800	1.97241600	-2.15888700
N	4.49544500	2.30014900	-0.00511500
H	5.01184400	2.12674000	-0.85639600
H	5.02652100	2.09559800	0.83019500
H	-1.68979210	1.43754495	2.12423988
O	-1.12863232	-3.80671746	4.25906235
O	-1.14848004	-3.83011030	-4.23543129
C	-1.24861077	-3.18526729	-5.50785077
H	-1.24715966	-3.92313161	-6.28274053
H	-2.15800418	-2.62319051	-5.55227273
H	-0.41559138	-2.52697503	-5.64062873
C	-1.20345487	-3.15475201	5.52959083
H	-2.11689587	-2.60114021	5.59314795
H	-1.17560758	-3.88760589	6.30872479
H	-0.37384719	-2.48767583	5.63757451

Compound 12

%mem=80GB
%nprocshared=8
opt freq 6-31++g(d,p) m062x pop=chelpg scf=tight

Title Card Required

0 1

C	-0.65891000	0.05227800	0.00077900
C	-0.76512000	-0.89332500	1.21161000
C	-2.00490100	-1.14770500	1.80245600
C	0.35021600	-1.64617100	1.60561200
C	-2.12348700	-2.10664300	2.80811400
H	-2.88888700	-0.60904700	1.47492500
C	0.22604100	-2.59596200	2.61544700
H	1.32077800	-1.51370900	1.11976800
C	-1.00687100	-2.82584100	3.22564300
H	-3.09358700	-2.29032100	3.25915100
H	1.10265300	-3.16428000	2.90951300
C	-1.65855200	1.22176100	-0.00488700
C	-2.07486700	1.80070800	-1.20843500
C	-2.02060300	1.84810100	1.19316100
C	-2.86021900	2.95192300	-1.21387900
H	-1.79246500	1.34885200	-2.15421300
C	-2.80573600	2.99867600	1.18997200
C	-3.23397200	3.55253000	-0.01437700
H	-3.17747700	3.37811800	-2.16029300
H	-3.08027400	3.46166600	2.13245200
C	-0.76107700	-0.89784500	-1.20709700
C	0.36388900	-1.60415000	-1.65376400
C	-2.01620800	-1.20640400	-1.73928900
C	0.22948500	-2.56158100	-2.65673900
H	1.35300600	-1.43958300	-1.21784700
C	-2.14354500	-2.16901800	-2.73882900
H	-2.90503500	-0.70529500	-1.36749000
C	-1.01892100	-2.84300300	-3.20891600
H	1.11362700	-3.09557100	-2.99005500
H	-3.12569500	-2.39276400	-3.14326700
Cl	3.57929200	-1.17651100	-0.00775300
N	0.69158500	0.74115900	0.00419400
C	1.27608000	1.10550700	1.17130100
C	1.25340900	1.14483700	-1.16095400
C	2.50209000	1.70228400	1.20679000

H	0.74515300	0.84577400	2.07672900
C	2.48028400	1.73990100	-1.20050000
H	0.70168100	0.91944700	-2.06339100
C	3.20383900	1.90480500	-0.00029200
H	2.96856300	1.90194800	2.16361100
H	2.92805800	1.97241600	-2.15888700
N	4.49544500	2.30014900	-0.00511500
H	5.01184400	2.12674000	-0.85639600
H	5.02652100	2.09559800	0.83019500
H	-1.68979210	1.43754495	2.12423988
O	-1.12863232	-3.80671746	4.25906235
O	-1.14848004	-3.83011030	-4.23543129
C	-1.24861077	-3.18526729	-5.50785077
H	-1.24715966	-3.92313161	-6.28274053
H	-2.15800418	-2.62319051	-5.55227273
H	-0.41559138	-2.52697503	-5.64062873
C	-1.20345487	-3.15475201	5.52959083
H	-2.11689587	-2.60114021	5.59314795
H	-1.17560758	-3.88760589	6.30872479
H	-0.37384719	-2.48767583	5.63757451
O	-4.04350271	4.73131685	-0.01902319
C	-5.42467681	4.36198317	0.01014448
H	-5.62341810	3.79412860	0.89498519
H	-6.02992288	5.24435285	0.00916844
H	-5.65415495	3.77111358	-0.85189549

4-aminopyridinium chloride

%mem=80GB
%nprocshared=8
opt freq 6-31++g(d,p) m062x pop=chelpg scf=tight

Title Card Required

0 1

C	-1.18934100	-1.13335900	0.00323300
C	0.19820700	-1.19575100	-0.00491800
C	0.92966200	-0.00000300	-0.00685500
C	0.19812300	1.19583100	-0.00486400
C	-1.18928700	1.13339200	0.00323500
N	-1.89772400	-0.00007100	0.00811800
H	-1.76614200	-2.05545500	0.00646100
H	0.70409400	-2.15649300	-0.01282100
H	0.70396900	2.15661300	-0.01275700
H	-1.76627600	2.05535900	0.00666900

N	2.30901900	-0.00006000	-0.06022600
H	2.78059200	0.84687600	0.21849200
H	2.78050600	-0.84664500	0.21972700
H	-2.89770023	-0.00008857	0.01501215
Cl	-5.05931936	-0.38360154	0.04959499

1-methylimidazolium chloride

%mem=80GB
%nprocshared=8
opt freq 6-31++g(d,p) m062x pop=chelpg scf=tight

Title Card Required

0 1

N	-0.33084000	0.41759400	0.00023000
C	0.44243300	-0.63095000	0.00025100
C	0.45002900	1.53908700	0.00000800
H	0.08896700	-1.64074200	0.00041600
C	1.73307200	1.12987900	-0.00011000
H	0.03109200	2.52128900	-0.00006500
H	2.64795800	1.68123600	-0.00024300
C	2.87421700	-1.12569300	-0.00016100
H	3.46775400	-0.94595800	-0.88591000
H	3.46908300	-0.94445900	0.88438400
H	2.53922800	-2.15236600	0.00098900
N	1.71044000	-0.25108900	0.00006600
Cl	-3.14764100	-0.32208900	-0.00010000
H	-1.38989300	0.31704500	0.00013400

Triphenylmethanol

%mem=80GB
%nprocshared=8
opt freq 6-31++g(d,p) m062x pop=chelpg scf=tight

Title Card Required

0 1

O	-0.04533300	-0.04830400	2.09211300
H	-0.54676600	0.67156400	2.43877800
C	-0.02959600	0.01297400	0.67797000
C	-1.42009600	0.50308900	0.22849400
C	-1.63185100	1.65561300	-0.51387500

C	-2.53579800	-0.22526200	0.65429400
C	-2.92306400	2.06645600	-0.83852500
H	-0.80222600	2.24876600	-0.84556400
C	-3.81694600	0.18195400	0.33514700
H	-2.39199800	-1.11733800	1.23632700
C	-4.01630500	1.33390200	-0.41906300
H	-3.06186500	2.96307400	-1.41635700
H	-4.65957200	-0.39619800	0.67108500
H	-5.01278300	1.65194000	-0.66979700
C	1.11447500	0.94013600	0.21665600
C	1.53006800	0.96282000	-1.11462800
C	1.76230500	1.77449600	1.11787200
C	2.54584900	1.80500300	-1.53193500
H	1.06422400	0.31623400	-1.83478000
C	2.78509800	2.62034200	0.70196400
H	1.49106000	1.75966200	2.15526600
C	3.17976000	2.64250700	-0.62318200
H	2.84706100	1.80209100	-2.56470400
H	3.27253200	3.25420800	1.42174300
H	3.97279300	3.29378200	-0.94533000
C	0.26290300	-1.42175800	0.20524000
C	1.12897200	-2.21696600	0.95286400
C	-0.26608000	-1.94028100	-0.96951900
C	1.45396000	-3.49616900	0.53371800
H	1.54225100	-1.83483500	1.86653800
C	0.06294800	-3.22210300	-1.39341900
H	-0.94659100	-1.35587300	-1.56130000
C	0.92316100	-4.00535400	-0.64380300
H	2.12159500	-4.09564800	1.12728800
H	-0.36110300	-3.60399800	-2.30544600
H	1.17547100	-4.99940100	-0.96827700

checkCIF (basic structural check) running

Checking for embedded fcf data in CIF ...
Found embedded fcf data in CIF. Extracting fcf data from uploaded CIF, please wait . . . .

checkCIF/PLATON (basic structural check)

Structure factors have been supplied for datablock(s) AndersonG_Im3Ph_Cl_1_0m

THIS REPORT IS FOR GUIDANCE ONLY. IF USED AS PART OF A REVIEW PROCEDURE FOR PUBLICATION, IT SHOULD NOT REPLACE THE EXPERTISE OF AN EXPERIENCED CRYSTALLOGRAPHIC REFEREE.

No syntax errors found.
Please wait while processing ....

CIF dictionary
Interpreting this report

Structure factor report

Datablock: AndersonG_Im3Ph_Cl_1_0m

```
Bond precision:      C-C = 0.0015 A              Wavelength=0.71073
Cell:        a=11.9138(5)      b=12.9386(5)     c=14.5793(6)
             alpha=86.1777(16) beta=77.9486(17) gamma=69.0609(15)
Temperature: 150 K
```

	Calculated	Reported
Volume	2052.62(15)	2052.62(15)
Space group	P -1	P -1
Hall group	-P 1	-P 1
Moiety formula	4(C23 H21 N2), 4(Cl), 7(H2 O)	4(C23 H21 N2), 4(Cl), 7(H2 O)
Sum formula	C92 H98 Cl4 N8 O7	C92 H98 Cl4 N8 O7
Mr	1569.58	1569.58
Dx,g cm-3	1.270	1.270
Z	1	1
Mu (mm-1)	0.205	0.205
F000	830.0	830.0
F000'	830.90	
h,k,lmax	18,19,22	18,19,22
Nref	15698	15670
Tmin,Tmax	0.897,0.978	0.715,0.747
Tmin'	0.897	

```
Correction method= # Reported T Limits: Tmin=0.715
Tmax=0.747 AbsCorr = MULTI-SCAN
Data completeness= 0.998        Theta(max)= 33.179
R(reflections)= 0.0409( 12936)      wR2(reflections)= 0.1169( 15670)
S = 1.015             Npar= 555
```

The following ALERTS were generated. Each ALERT has the format
test-name_ALERT_alert-type_alert-level.
Click on the hyperlinks for more details of the test.

Alert level C

```
PLAT260_ALERT_2_C Large Average Ueq of Residue Including        O6      0.120 Check
PLAT910_ALERT_3_C Missing # of FCF Reflection(s) Below Theta(Min).        7 Note
```

```
PLAT911_ALERT_3_C Missing FCF Refl Between Thmin & STh/L=    0.600          12 Report
```

Alert level G

```
PLAT002_ALERT_2_G Number of Distance or Angle Restraints on AtSite          19 Note
PLAT003_ALERT_2_G Number of Uiso or Uij Restrained non-H Atoms ...           4 Report
PLAT007_ALERT_5_G Number of Unrefined Donor-H Atoms ..............           6 Report
PLAT172_ALERT_4_G The CIF-Embedded .res File Contains DFIX Records           3 Report
PLAT178_ALERT_4_G The CIF-Embedded .res File Contains SIMU Records           2 Report
PLAT300_ALERT_4_G Atom Site Occupancy of Cl2        Constrained at         0.5 Check
```

And 18 other PLAT300 Alerts

```
PLAT300_ALERT_4_G Atom Site Occupancy of Cl3        Constrained at         0.5 Check
PLAT300_ALERT_4_G Atom Site Occupancy of H1B        Constrained at         0.5 Check
PLAT300_ALERT_4_G Atom Site Occupancy of H1C        Constrained at         0.5 Check
PLAT300_ALERT_4_G Atom Site Occupancy of O2         Constrained at         0.5 Check
PLAT300_ALERT_4_G Atom Site Occupancy of H2A        Constrained at         0.5 Check
PLAT300_ALERT_4_G Atom Site Occupancy of H2B        Constrained at         0.5 Check
PLAT300_ALERT_4_G Atom Site Occupancy of O3         Constrained at         0.5 Check
PLAT300_ALERT_4_G Atom Site Occupancy of H3A        Constrained at         0.5 Check
PLAT300_ALERT_4_G Atom Site Occupancy of H3B        Constrained at         0.5 Check
PLAT300_ALERT_4_G Atom Site Occupancy of O4         Constrained at         0.5 Check
PLAT300_ALERT_4_G Atom Site Occupancy of H4A        Constrained at         0.5 Check
PLAT300_ALERT_4_G Atom Site Occupancy of H4B        Constrained at         0.5 Check
PLAT300_ALERT_4_G Atom Site Occupancy of O5         Constrained at         0.5 Check
PLAT300_ALERT_4_G Atom Site Occupancy of H5D        Constrained at         0.5 Check
PLAT300_ALERT_4_G Atom Site Occupancy of H5E        Constrained at         0.5 Check
PLAT300_ALERT_4_G Atom Site Occupancy of O6         Constrained at         0.5 Check
PLAT300_ALERT_4_G Atom Site Occupancy of H6A        Constrained at         0.5 Check
PLAT300_ALERT_4_G Atom Site Occupancy of H6B        Constrained at         0.5 Check

PLAT302_ALERT_4_G Anion/Solvent/Minor-Residue Disorder (Resd  4 )         100% Note
```

And 6 other PLAT302 Alerts

```
PLAT302_ALERT_4_G Anion/Solvent/Minor-Residue Disorder (Resd  5 )         100% Note
PLAT302_ALERT_4_G Anion/Solvent/Minor-Residue Disorder (Resd  7 )         100% Note
PLAT302_ALERT_4_G Anion/Solvent/Minor-Residue Disorder (Resd  8 )         100% Note
PLAT302_ALERT_4_G Anion/Solvent/Minor-Residue Disorder (Resd  9 )         100% Note
PLAT302_ALERT_4_G Anion/Solvent/Minor-Residue Disorder (Resd 10 )         100% Note
PLAT302_ALERT_4_G Anion/Solvent/Minor-Residue Disorder (Resd 11 )         100% Note

PLAT304_ALERT_4_G Non-Integer Number of Atoms in ..... (Resd  4 )         0.50 Check
```

And 6 other PLAT304 Alerts

```
PLAT304_ALERT_4_G Non-Integer Number of Atoms in ..... (Resd  5 )         0.50 Check
PLAT304_ALERT_4_G Non-Integer Number of Atoms in ..... (Resd  7 )         1.50 Check
PLAT304_ALERT_4_G Non-Integer Number of Atoms in ..... (Resd  8 )         1.50 Check
PLAT304_ALERT_4_G Non-Integer Number of Atoms in ..... (Resd  9 )         1.50 Check
PLAT304_ALERT_4_G Non-Integer Number of Atoms in ..... (Resd 10 )         1.50 Check
PLAT304_ALERT_4_G Non-Integer Number of Atoms in ..... (Resd 11 )         1.50 Check

PLAT789_ALERT_4_G Atoms with Negative _atom_site_disorder_group  #          11 Check
PLAT860_ALERT_3_G Number of Least-Squares Restraints .............          34 Note
PLAT912_ALERT_4_G Missing # of FCF Reflections Above STh/L=  0.600           8 Note
PLAT913_ALERT_3_G Missing # of Very Strong Reflections in FCF ....           3 Note
PLAT933_ALERT_2_G Number of OMIT Records in Embedded .res File ...           2 Note
PLAT978_ALERT_2_G Number C-C Bonds with Positive Residual Density.          21 Info
PLAT992_ALERT_5_G Repd & Actual _reflns_number_gt Values Differ by           3 Check
```

```
 0 ALERT level A = Most likely a serious problem - resolve or explain
 0 ALERT level B = A potentially serious problem, consider carefully
 3 ALERT level C = Check. Ensure it is not caused by an omission or oversight
45 ALERT level G = General information/check it is not something unexpected

 0 ALERT type 1 CIF construction/syntax error, inconsistent or missing data
 5 ALERT type 2 Indicator that the structure model may be wrong or deficient
 4 ALERT type 3 Indicator that the structure quality may be low
37 ALERT type 4 Improvement, methodology, query or suggestion
 2 ALERT type 5 Informative message, check
```

It is advisable to attempt to resolve as many as possible of the alerts in all categories. Often the minor

alerts point to easily fixed oversights, errors and omissions in your CIF or refinement strategy, so attention to these fine details can be worthwhile. In order to resolve some of the more serious problems it may be necessary to carry out additional measurements or structure refinements. However, the purpose of your study may justify the reported deviations and the more serious of these should normally be commented upon in the discussion or experimental section of a paper or in the "special_details" fields of the CIF. checkCIF was carefully designed to identify outliers and unusual parameters, but every test has its limitations and alerts that are not important in a particular case may appear. Conversely, the absence of alerts does not guarantee there are no aspects of the results needing attention. It is up to the individual to critically assess their own results and, if necessary, seek expert advice.

Publication of your CIF in IUCr journals

A basic structural check has been run on your CIF. These basic checks will be run on all CIFs submitted for publication in IUCr journals (*Acta Crystallographica*, *Journal of Applied Crystallography*, *Journal of Synchrotron Radiation*); however, if you intend to submit to *Acta Crystallographica Section C* or *E* or *IUCrData*, you should make sure that full publication checks are run on the final version of your CIF prior to submission.

Publication of your CIF in other journals

Please refer to the *Notes for Authors* of the relevant journal for any special instructions relating to CIF submission.

PLATON version of 04/06/2020; check.def file version of 02/06/2020

Datablock AndersonG_Im3Ph_Cl_1_0m - ellipsoid plot

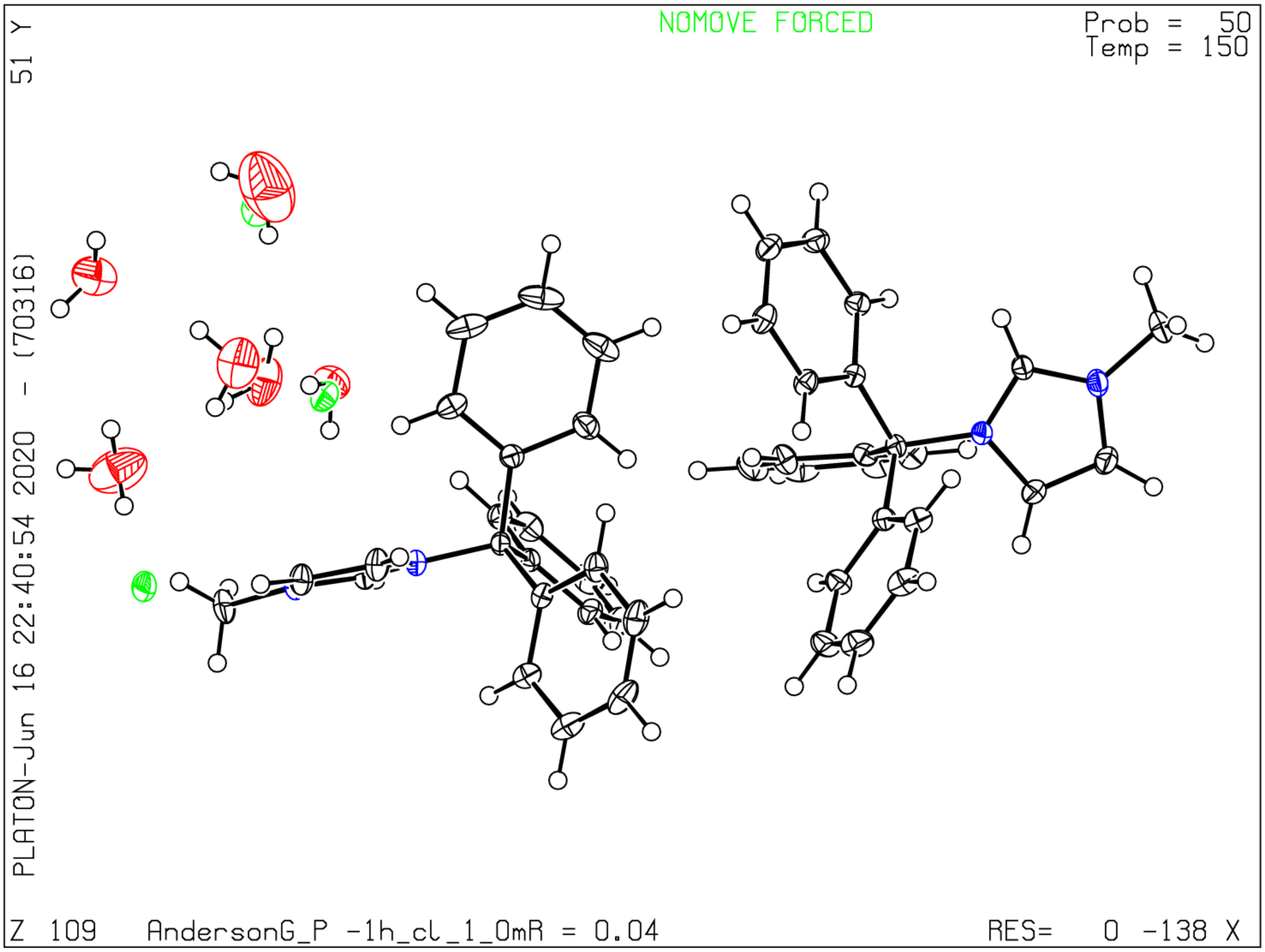

Download CIF editor (publCIF) from the IUCr
Download CIF editor (enCIFer) from the CCDC
Test a new CIF entry

checkCIF (basic structural check) running

Checking for embedded fcf data in CIF ...
Found embedded fcf data in CIF. Extracting fcf data from uploaded CIF, please wait . . . .

checkCIF/PLATON (basic structural check)

Structure factors have been supplied for datablock(s) Ampy3Ph_Cl_Anderson_100520_0m

THIS REPORT IS FOR GUIDANCE ONLY. IF USED AS PART OF A REVIEW PROCEDURE FOR PUBLICATION, IT SHOULD NOT REPLACE THE EXPERTISE OF AN EXPERIENCED CRYSTALLOGRAPHIC REFEREE.

No syntax errors found.
Please wait while processing ....

CIF dictionary
Interpreting this report

Structure factor report

Datablock: Ampy3Ph_Cl_Anderson_100520_0m

Bond precision: C-C = 0.0021 A Wavelength=0.71073

Cell: a=17.6483(10) b=14.0704(6) c=38.934(2)
alpha=90 beta=90 gamma=90

Temperature: 150 K

	Calculated	Reported
Volume	9668.0(8)	9668.0(9)
Space group	P b c a	P b c a
Hall group	-P 2ac 2ab	-P 2ac 2ab
Moiety formula	2(C24 H21 N2), C5 H7 N2, C H4 O, 3(Cl), H2 O	2(C24 H21 N2), C5 H7 N2, C H4 O, 3(Cl), H2 O
Sum formula	C54 H55 Cl3 N6 O2	C54 H55 Cl3 N6 O2
Mr	926.39	926.39
Dx,g cm-3	1.273	1.273
Z	8	8
Mu (mm-1)	0.238	0.238
F000	3904.0	3904.0
F000'	3908.83	
h,k,lmax	27,21,59	23,21,59
Nref	18459	15376
Tmin,Tmax	0.936,0.951	0.663,0.747
Tmin'	0.877	

Correction method= # Reported T Limits: Tmin=0.663 Tmax=0.747
AbsCorr = MULTI-SCAN

Data completeness= 0.833 Theta(max)= 33.159

R(reflections)= 0.0476(11162) wR2(reflections)= 0.1247(15376)

S = 1.018 Npar= 604

The following ALERTS were generated. Each ALERT has the format
test-name_ALERT_alert-type_alert-level.
Click on the hyperlinks for more details of the test.

Alert level C

```
PLAT906_ALERT_3_C Large K Value in the Analysis of Variance ......      5.410 Check
PLAT910_ALERT_3_C Missing # of FCF Reflection(s) Below Theta(Min).          6 Note
PLAT911_ALERT_3_C Missing FCF Refl Between Thmin & STh/L=    0.600          5 Report
PLAT913_ALERT_3_C Missing # of Very Strong Reflections in FCF ....          5 Note
```

Alert level G

```
PLAT002_ALERT_2_G Number of Distance or Angle Restraints on AtSite          3 Note
PLAT007_ALERT_5_G Number of Unrefined Donor-H Atoms .............          8 Report
PLAT172_ALERT_4_G The CIF-Embedded .res File Contains DFIX Records          1 Report
PLAT302_ALERT_4_G Anion/Solvent/Minor-Residue Disorder (Resd  7  )       100% Note
PLAT302_ALERT_4_G Anion/Solvent/Minor-Residue Disorder (Resd  8  )       100% Note
PLAT304_ALERT_4_G Non-Integer Number of Atoms in ..... (Resd  7  )       0.89 Check
PLAT304_ALERT_4_G Non-Integer Number of Atoms in ..... (Resd  8  )       0.11 Check
PLAT432_ALERT_2_G Short Inter X...Y Contact  Cl3B     ..C54              2.65 Ang.
                                            3/2-x,1/2+y,z  =      8_765 Check
PLAT860_ALERT_3_G Number of Least-Squares Restraints ............          2 Note
PLAT912_ALERT_4_G Missing # of FCF Reflections Above STh/L=  0.600       2591 Note
PLAT950_ALERT_5_G Calculated (ThMax) and CIF-Reported Hmax Differ           4 Units
PLAT956_ALERT_1_G Calculated (ThMax) and Actual (FCF) Hmax Differ           4 Units
PLAT978_ALERT_2_G Number C-C Bonds with Positive Residual Density.        20 Info
```

```
  0 ALERT level A = Most likely a serious problem - resolve or explain
  0 ALERT level B = A potentially serious problem, consider carefully
  4 ALERT level C = Check. Ensure it is not caused by an omission or oversight
 13 ALERT level G = General information/check it is not something unexpected

  1 ALERT type 1 CIF construction/syntax error, inconsistent or missing data
  3 ALERT type 2 Indicator that the structure model may be wrong or deficient
  5 ALERT type 3 Indicator that the structure quality may be low
  6 ALERT type 4 Improvement, methodology, query or suggestion
  2 ALERT type 5 Informative message, check
```

It is advisable to attempt to resolve as many as possible of the alerts in all categories. Often the minor alerts point to easily fixed oversights, errors and omissions in your CIF or refinement strategy, so attention to these fine details can be worthwhile. In order to resolve some of the more serious problems it may be necessary to carry out additional measurements or structure refinements. However, the purpose of your study may justify the reported deviations and the more serious of these should normally be commented upon in the discussion or experimental section of a paper or in the "special_details" fields of the CIF. checkCIF was carefully designed to identify outliers and unusual parameters, but every test has its limitations and alerts that are not important in a particular case may appear. Conversely, the absence of alerts does not guarantee there are no aspects of the results needing attention. It is up to the individual to critically assess their own results and, if necessary, seek expert advice.

Publication of your CIF in IUCr journals

A basic structural check has been run on your CIF. These basic checks will be run on all CIFs submitted for publication in IUCr journals (*Acta Crystallographica*, *Journal of Applied Crystallography*, *Journal of Synchrotron Radiation*); however, if you intend to submit to *Acta Crystallographica Section C* or *E* or *IUCrData*, you should make sure that full publication checks are run on the final version of your CIF prior to submission.

Publication of your CIF in other journals

Please refer to the *Notes for Authors* of the relevant journal for any special instructions relating to CIF submission.

PLATON version of 18/09/2020; check.def file version of 20/08/2020

Datablock Ampy3Ph_Cl_Anderson_100520_0m - ellipsoid plot

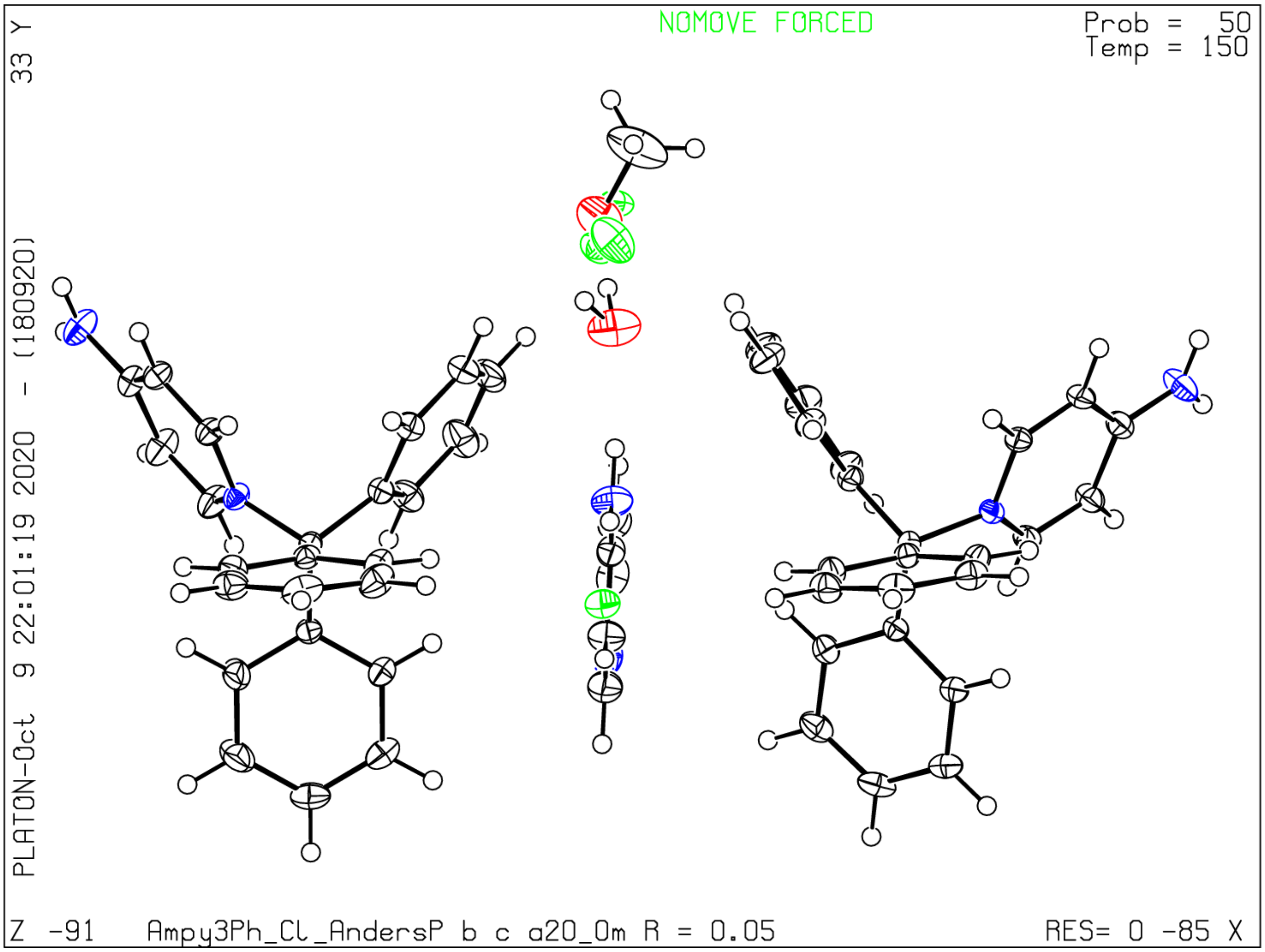

[Download CIF editor (publCIF) from the IUCr](#)
[Download CIF editor (enCIFer) from the CCDC](#)
[Test a new CIF entry](#)

checkCIF (basic structural check) running

Checking for embedded fcf data in CIF ...
Found embedded fcf data in CIF. Extracting fcf data from uploaded CIF, please wait ....

checkCIF/PLATON (basic structural check)

Structure factors have been supplied for datablock(s) AndersonG_ImPh2PhCl_Cl_1_0m

THIS REPORT IS FOR GUIDANCE ONLY. IF USED AS PART OF A REVIEW PROCEDURE FOR PUBLICATION, IT SHOULD NOT REPLACE THE EXPERTISE OF AN EXPERIENCED CRYSTALLOGRAPHIC REFEREE.

```
No syntax errors found.
Please wait while processing ....
```

CIF dictionary
Interpreting this report

Structure factor report

Datablock: AndersonG_ImPh2PhCl_Cl_1_0m

```
Bond precision:     C-C = 0.0077 A              Wavelength=1.54178
Cell:        a=8.4708(13)    b=19.356(4)     c=36.259(8)
             alpha=90        beta=90         gamma=90
Temperature:150 K
```

	Calculated	Reported
Volume	5945(2)	5945(2)
Space group	P 21 21 21	P 21 21 21
Hall group	P 2ac 2ab	P 2ac 2ab
Moiety formula	C19 H15 Cl O	?
Sum formula	C19 H15 Cl O	C19 H15 Cl O
Mr	294.76	294.76
Dx,g cm-3	1.317	1.317
Z	16	16
Mu (mm-1)	2.224	2.224
F000	2464.0	2464.0
F000'	2475.90	
h,k,lmax	10,24,46	10,24,45
Nref	12947[7203]	11591
Tmin,Tmax	0.808,0.935	0.571,0.754
Tmin'	0.586	

```
Correction method= # Reported T Limits: Tmin=0.571
Tmax=0.754 AbsCorr = MULTI-SCAN
Data completeness= 1.61/0.90    Theta(max)= 79.752
R(reflections)= 0.0574( 9934)      wR2(reflections)= 0.1564( 11591)
S = 1.034             Npar= 762
```

The following ALERTS were generated. Each ALERT has the format
test-name_ALERT_alert-type_alert-level.
Click on the hyperlinks for more details of the test.

Alert level C

```
PLAT340_ALERT_3_C Low Bond Precision on  C-C Bonds ...............     0.0077 Ang.
PLAT911_ALERT_3_C Missing FCF Refl Between Thmin & STh/L=    0.600       117 Report
```

```
PLAT915_ALERT_3_C No Flack x Check Done: Low Friedel Pair Coverage         84 %
```

Alert level G

```
PLAT007_ALERT_5_G Number of Unrefined Donor-H Atoms ..............          4 Report
PLAT033_ALERT_4_G Flack x Value Deviates > 3.0 * sigma from Zero .      0.098 Note
PLAT912_ALERT_4_G Missing # of FCF Reflections Above STh/L=  0.600        331 Note
PLAT913_ALERT_3_G Missing # of Very Strong Reflections in FCF ....          1 Note
PLAT933_ALERT_2_G Number of OMIT Records in Embedded .res File ...          5 Note
PLAT941_ALERT_3_G Average HKL Measurement Multiplicity ...........        4.2 Low
PLAT965_ALERT_2_G The SHELXL WEIGHT Optimisation has not Converged     Please Check
PLAT978_ALERT_2_G Number C-C Bonds with Positive Residual Density.          1 Info
PLAT992_ALERT_5_G Repd & Actual _reflns_number_gt Values Differ by          1 Check
```

```
   0 ALERT level A = Most likely a serious problem - resolve or explain
   0 ALERT level B = A potentially serious problem, consider carefully
   3 ALERT level C = Check. Ensure it is not caused by an omission or oversight
   9 ALERT level G = General information/check it is not something unexpected

   0 ALERT type 1 CIF construction/syntax error, inconsistent or missing data
   3 ALERT type 2 Indicator that the structure model may be wrong or deficient
   5 ALERT type 3 Indicator that the structure quality may be low
   2 ALERT type 4 Improvement, methodology, query or suggestion
   2 ALERT type 5 Informative message, check
```

It is advisable to attempt to resolve as many as possible of the alerts in all categories. Often the minor alerts point to easily fixed oversights, errors and omissions in your CIF or refinement strategy, so attention to these fine details can be worthwhile. In order to resolve some of the more serious problems it may be necessary to carry out additional measurements or structure refinements. However, the purpose of your study may justify the reported deviations and the more serious of these should normally be commented upon in the discussion or experimental section of a paper or in the "special_details" fields of the CIF. checkCIF was carefully designed to identify outliers and unusual parameters, but every test has its limitations and alerts that are not important in a particular case may appear. Conversely, the absence of alerts does not guarantee there are no aspects of the results needing attention. It is up to the individual to critically assess their own results and, if necessary, seek expert advice.

Publication of your CIF in IUCr journals

A basic structural check has been run on your CIF. These basic checks will be run on all CIFs submitted for publication in IUCr journals (*Acta Crystallographica*, *Journal of Applied Crystallography*, *Journal of Synchrotron Radiation*); however, if you intend to submit to *Acta Crystallographica Section C* or *E* or *IUCrData*, you should make sure that full publication checks are run on the final version of your CIF prior to submission.

Publication of your CIF in other journals

Please refer to the *Notes for Authors* of the relevant journal for any special instructions relating to CIF submission.

PLATON version of 04/06/2020; check.def file version of 02/06/2020

Datablock AndersonG_ImPh2PhCl_Cl_1_0m - ellipsoid plot

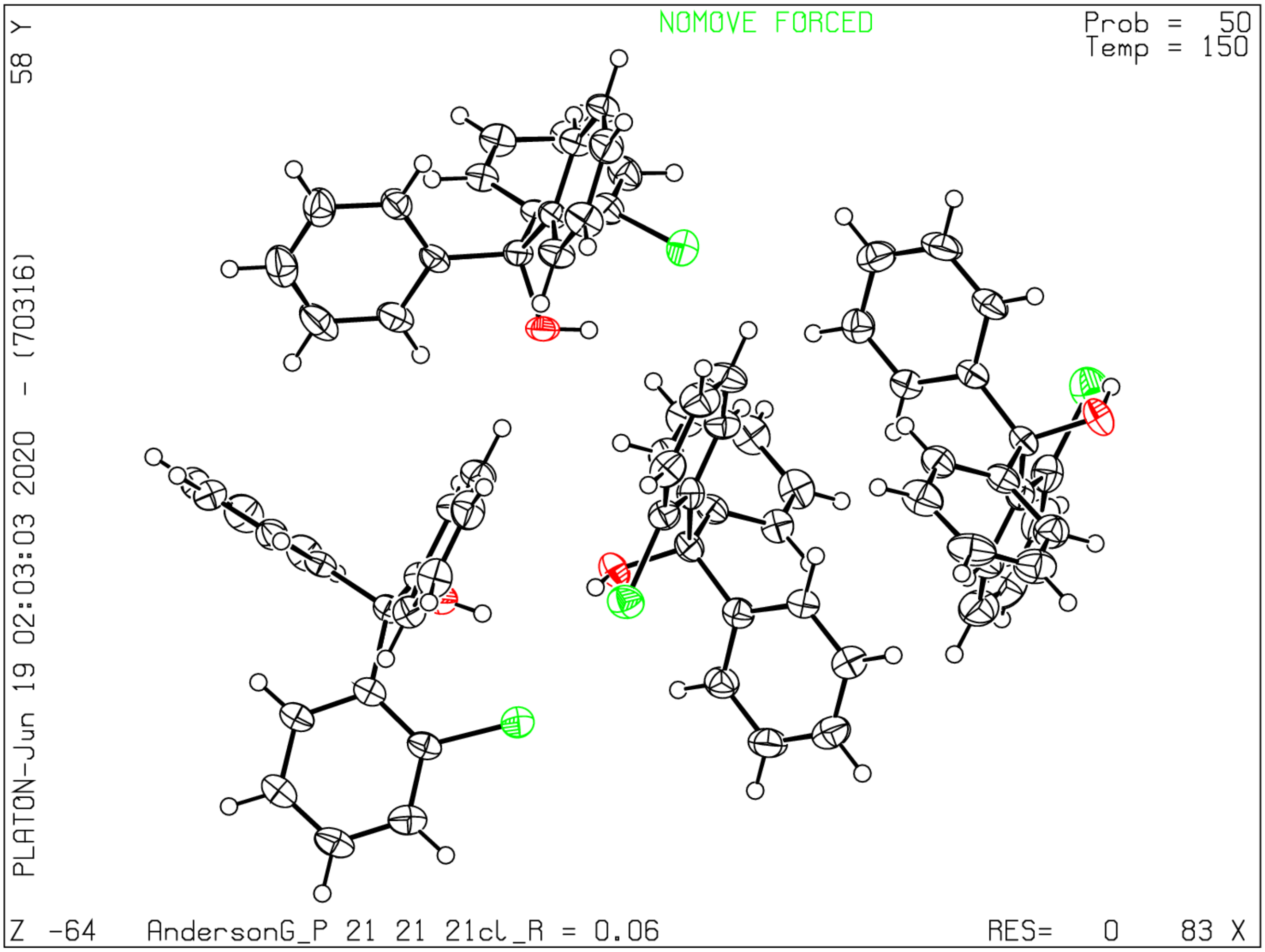

Download CIF editor (publCIF) from the IUCr
Download CIF editor (enCIFer) from the CCDC
Test a new CIF entry
